# A Green Lindqvist-Type POM/TEMPO/H_2_O_2_ Ternary Catalytic System for the Selective Oxidation of Hantzsch 1,4-Dihydropyridines (1,4-DHPs) to Pyridines

**DOI:** 10.3390/molecules31193529

**Published:** 2026-10-03

**Authors:** Qiang Zeng, Xiaoxiao Yan, Shaohong Hou, Yutong Zhang, Jiuzhong Huang, Gaorong Wu, Dan Miao, Feng Jiang, Weiming Chen

**Affiliations:** School of Pharmacy, Gannan Medical University, Ganzhou 341000, China; 10070974127@163.com (Q.Z.); yanxx2025@gmu.edu.cn (X.Y.); 19914798511@163.com (S.H.); 15083720505@163.com (Y.Z.); huang9zhong@126.com (J.H.); gaorongwu09@163.com (G.W.); miaodan2015@163.com (D.M.)

**Keywords:** ternary catalytic system, Lindqvist-type POM, TEMPO, aromatization, 1,4-DHPs

## Abstract

Pyridine derivatives are prepared via the dehydrogenative aromatization of 1,4-dihydropyridines (1,4-DHPs). Among these methods, stoichiometric catalysis with high waste and electrochemical oxidation with less productivity are limited in their use. Notably, methyl groups and nitrogen atoms may impose limitations on oxidation. We revealed the efficient strategy via recyclable Lindqvist-type polyoxometalate (POM) (*n*-Bu_4_N)_2_[Mo_6_O_19_] and 2,2,6,6-tetramethylpiperidin-1-oxyl (TEMPO) as co-catalysts using the green and economical oxidant hydrogen peroxide. The method applied to the aromatization of a large amount of 1,4-dihydropyridines (1,4-DHPs) incorporating multiple oxidation-prone groups to pyridines with a high conversation rate and selectivity. The Lindqvist-type POM/TEMPO/H_2_O_2_ ternary catalytic system was verified on a larger scale; meanwhile, after being recycled for six times, the Lindqvist-type POM catalyst could be used for the oxidation without obvious loss in reaction time and isolated yield (up to 90%). The mechanism of the ternary catalytic system was proposed by the control experiments.

## 1. Introduction

The aromatization reaction is a class of common and important oxidation processes [1]. In recent years, significant research efforts have been devoted to investigating various aromatization reactions, including the oxidation of cyclohexadienes [2], dihydropyridines [3], indoline [4], pyrimidines [5], and dihydroquinoline [6], among others. Thus, the investigation of aromatization reactions remains highly relevant and necessary.

A considerable challenge in oxidation chemistry is achieving high selectivity in the presence of multiple functional groups, such as double bonds, amines, pyridines, thiophenes, phenolic hydroxyl groups, and benzyl groups. The requirement for protective groups in oxidation reactions is problematic, as it increases both the complexity and the number of synthetic steps, thereby conflicting with key green chemistry principles such as atom economy and waste reduction. For instance, 1,4-dihydropyridines (1,4-DHPs) serve as the core structure of many calcium channel antagonists, which are widely used as antihypertensive drugs [7]. This heterocyclic system is prone to oxidation, leading to aromatization, which contains functional groups such as pyridyl nitrogen, methyl, and the amino group. Current methods often involve the use of precious metals or other harsh conditions, which omit consideration of polyfunctional systems and have drawbacks such as difficulty in recycling and stringent reaction requirements [8].

Polyoxometalates (POMs), characterized by a unique structure in which a single central metal atom is supported by an inorganic ring, constitute a broad class of structurally well-defined anionic metal–oxygen clusters [9]. These compounds exhibit enhanced thermal and redox stability compared to conventional organometallic complexes [10]. In recent years, polyoxometalates have been a hot topic in the field of metal ion catalysts and have been used for oxidation and other reactions, including the conversion of aldehydes to carboxylic acids [11], alcohols to carboxylic acids [12], alcohols to esters [13], amines to imines [14], amines to *N*-formamides [15], C–H bonds to ketones [16], aldehydes to formamides [17], alcohols to *N*-formamides, epoxidation of olefins [18], and oxidative esterification of alcohols [19]. These catalytic systems completely avoid the need for complicated or sensitive organic ligands, and produce only water as a byproduct when oxygen or hydrogen peroxide is used as the oxidant. Moreover, owing to their high stability and low solubility in both aqueous and organic media, the catalysts exhibit excellent reusability.

For the aromatization of 1,4-DHPs [20], compared to other metal catalysts such as Ni(NO_3_)_3_ [21], KMnO_4_ [22], V_2_O_5_ [23], Na_2_S_2_O_6_/CuI [24], NaI [25], Co(OAc)_2_ [26], and CrO_2_ [27], the polyoxometalates offer potential advantages such as mild reaction conditions, environmental friendliness, and ease of recovery.

In this study, we prepared a Lindqvist-type molybdenum-based polyoxometalate (*n*-Bu_4_N)_2_[Mo_6_O_19_] (CCDC number: 2584233), which played a crucial role in aromatization reactions by modulating the catalytic efficiency of the primary catalyst [28,29,30,31,32,33]. Notably, this compound itself exhibits negligible intrinsic activity. Instead, it functions as a modifier that enhances catalytic performance without participating directly in the reaction. A key result is that even in the absence of the polyoxometalate, the primary catalyst maintains a baseline level of activity, though with moderately reduced efficiency. Building on this discovery, we have conducted extensive research that has led to significant advances in understanding and applying this catalytic system.

Herein, we report the catalyst (*n*-Bu_4_N)_2_[Mo_6_O_19_] in combination with hydrogen peroxide as a green oxidant. Preliminary experiments showed that adding catalytic TEMPO [34] significantly improved yield while reducing generation of nitrogen oxide byproducts. Consequently, we optimized the reaction conditions to establish a practical protocol with low oxidant loading, high efficiency, and a high conversion rate. This catalyst selection offers advantages in terms of mild conditions, environmental compatibility, and ease of use.

## 2. Results and Discussion

Based on our previous work, the oxidation of diethyl 2,6-dimethylpyridine-3,5-dicarboxylate (**1a**) was initially studied using the Lindqvist-type catalyst (*n*-Bu_4_N)_2_[Mo_6_O_19_] with hydrogen peroxide as the oxidant in acetonitrile at 80 °C on a 0.5 mmol scale. As presented in Table 1, the reaction proceeded rapidly, completing within one hour, but afforded a low isolated yield of only about 60% (Entry 1), indicating limited synthetic efficiency. The suspicion that the byproducts were nitrogen oxide impurities and polymers, arising from the many oxidizable nitrogen and methyl groups, prompted us to verify the roles of the catalyst and oxidant. This was done by examining the reaction outcome when each component was employed individually. As shown in Entry 2, the use of the catalyst alone resulted in trace conversion of the starting material after 24 h. Conversely, when hydrogen peroxide was employed alone (Entry 3), the reaction time was significantly prolonged, albeit without the generation of significant impurities. Subsequent screening of the oxidation conditions, including optimization of the oxidant amount, identified the combination of both components as the optimal reaction conditions.

The reaction with 2,2,6,6-tetramethylpiperidin-1-oxyl TEMPO yielded a surprising outcome, showing dramatically less impurities as well as shorter reaction time and affording an excellent isolated yield of 95% (Entry 4). Further experimental screenings (Entries 5–7) revealed that TEMPO enhances the oxidation selectivity. It is thus concluded that TEMPO is indispensable, and the ternary co-catalysis system comprising the catalyst, oxidant, and TEMPO is essential for optimal performance.

As shown in Table 2, we further examined the reaction conditions by varying the amount of TEMPO. The reaction efficiency remained even with reduced loading (Entries 1 and 2), suggesting that TEMPO acts as a co-catalyst in this system. Subsequently, the types of catalyst (10% NaClO, Fe(NO_3_)_3_, and Na_2_MoO_4_) and solvents (DCM, MeOH, DCE) were investigated. It was found that more impurities appeared in the reaction system or the reaction time was prolonged when these catalysts were used (Entries 3–6). Furthermore, the influence of oxidant loading was examined across a range of 0.2 equiv. to 1.2 equiv.; the reaction was found to proceed slowly or incompletely when the oxidant was insufficient (Entries 7–9). This limitation prompted an investigation into the addition protocol, postulating that sequential addition could enhance performance. Experimental results substantiated this postulate, demonstrating the superior efficacy of the batch addition method for hydrogen peroxide (Entry 9).

To avoid high-temperature oxidant deactivation, we next optimized the reaction temperature. While reducing the temperature from 80 °C to 60 °C improved the outcome (Entry 10), a further decrease caused system turbidity and poor efficiency, proving that it was not the optimal choice (Entries 11 and 12). This identified optimum at 60 °C was rationalized by considering the decomposition temperature of hydrogen peroxide, which was particularly relevant given the positive results from its batch addition. The solvent was systematically evaluated under the optimized reaction conditions. Screening revealed that solvents including HFIP, MeOH, EtOAc, and THF led to variable reaction times and unsatisfactory isolated yields (Entries 13–16). Acetonitrile was identified as the superior solvent, delivering the highest yield and thus establishing itself as the optimal choice for this transformation.

We finally tested a series of TEMPO analogs (TEMPOL, 4-hydroxy-2,2,6,6-tetramethyl-1-piperidinyloxy; TEMP, 2,2,6,6-tetramethylpiperidine; TEMPOH, 1-hydroxy-2,2,6,6-tetramethylpiperidine). Only the structurally similar radical species TEMPOL provided a satisfactory yield (Entry 17). All other analogues proved to be ineffective, prolonging the reaction time and generating significant impurities with low yields by TLC (Entries 18–19). Therefore, a structural and functional resemblance to TEMPO is essential for an effective co-catalyst in this reaction.

**Scheme 1:**
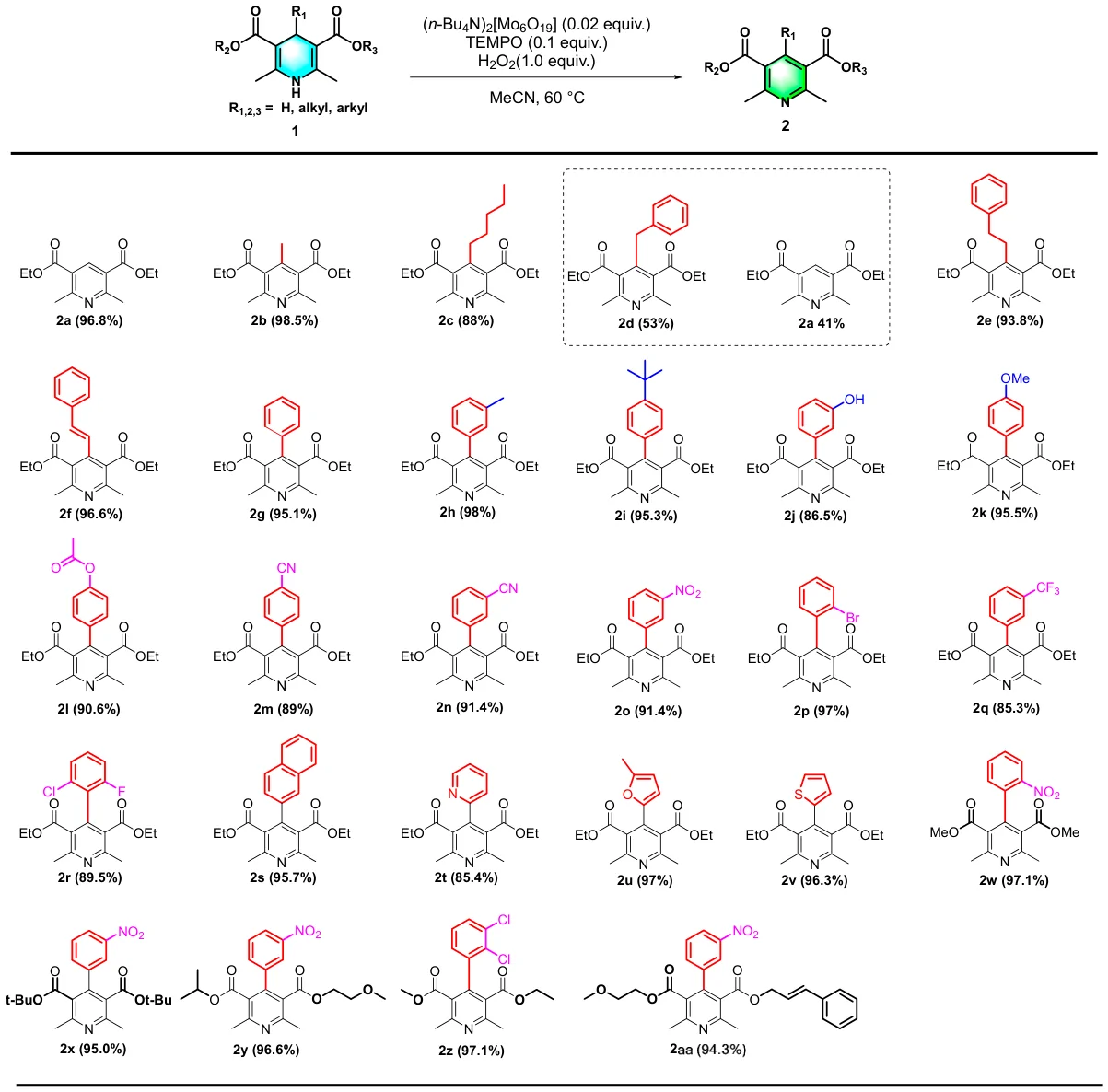
Reaction applicability for substituted 1,4-DHPs ^[a]^. Notes: ^[a]^ Reaction conditions: 1,4-DHPs (0.5 mmol, 1 equiv.), catalyst (*n*-Bu_4_N)_2_[Mo_6_O_19_] (0.02 equiv.), TEMPO (0.1 equiv.) and 30% H_2_O_2_ (0.4~0.5 equiv.*3) in MeCN (2 mL) were stirred at 60 °C. The details are summarized in the Supplementary Information (SI).

The substrate scope of the (*n*-Bu_4_N)_2_[Mo_6_O_19_]/H_2_O_2_/TEMPO catalytic system was explored with various substituted 1,4-DHPs (Scheme 1). Notably, excellent isolated yields (over 88%) were obtained for substrates featuring alkyl groups (**2b**, **2c**).

The reaction pathway was highly dependent on the 4-position substituent. A benzyl group induced a divergent pathway, yielding a nearly 1:1 mixture of the direct aromatization product (**2d**) and the debenzylated derivative (**2a**), which indicated that the reaction may be a radical pathway. This contrasts with phenethyl and styryl groups, which proceeded cleanly to the desired products in over 93% yield within a short time (**2e**, **2f**), suggesting a sensitivity to the benzylic position’s stability.

A pronounced electronic effect was observed for aryl substituents. EDGs on the aryl ring accelerated the reaction and ensured high yields (**2h**–**2l**). EWGs, in contrast, substantially decelerated the process despite ultimately achieving similar high yields (~90%), indicating an impact on the reaction rate but not the feasibility (**2m**–**2r**).

Furthermore, heterocyclic substituents were viable but led to slower reaction kinetics (**2t**–**2v**). Importantly, the reaction demonstrated broad tolerance toward variations in the ester groups, as different esters were obtained in high yields without compromising efficiency (**2w**–**2aa**).

Notably, when the substrate contains functional groups such as benzyl groups (**2b**, **2c**, **2d**, **2e**, **2h**), double bond (**2f**), pyridine (**2t**), thiophene (**2v**), and the phenolic hydroxyl group (**2j**), the reaction demonstrated excellent regioselectivity under reaction conditions, affording the oxidized products good yields.

**Figure 1:**
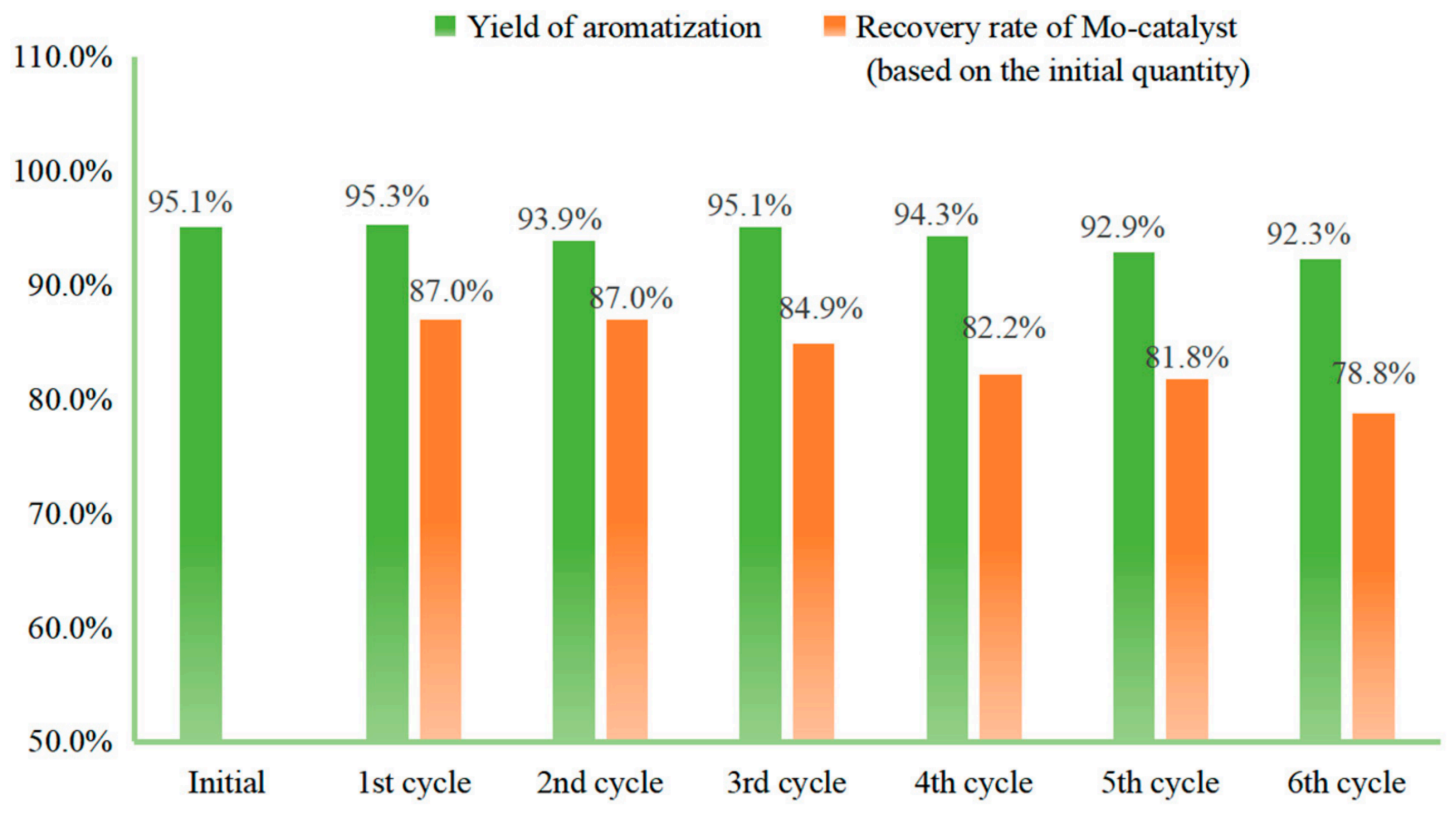
The recycled results of the Lindqvist-type catalyst.

To evaluate the stability and practical applicability of the catalyst, recycling experiments were carried out following the oxidation reaction on a 10 g scale. After separation and purification, 9.4 g of the aromatized product was obtained, corresponding to an isolated yield of 95.1%. The catalyst recovered according to the method in the Supplementary Information from this scale was reused under identical conditions over six cycles (Figure 1), affording consistent oxidation yields of over 90%, which were comparable to those achieved with the fresh catalyst. Meanwhile, the recovery rate of the catalyst remained at around 80% with a slight decrease during the six cycles. High-resolution mass spectrometry (HRMS), FT-IR spectra, and X-ray diffraction analysis (see the Supplementary Information file) further confirmed that the structure of the recovered catalyst remained unchanged, indicating no alteration in its properties during the reaction.

**Scheme 2:**
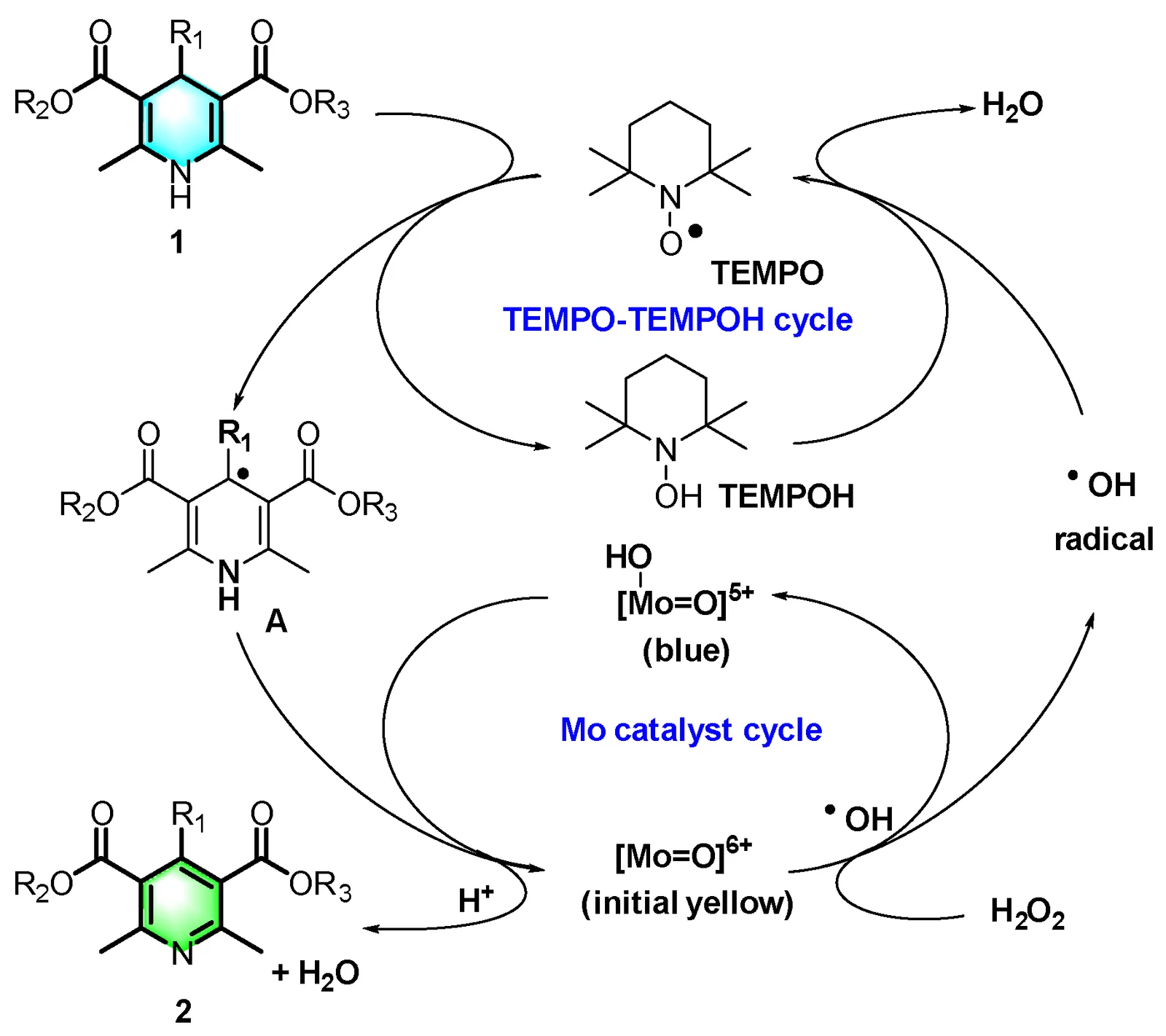
Proposed reaction mechanism.

Based on the literature precedents and obtained results, we propose that the molybdenum-based catalyst promotes the decomposition of hydrogen peroxide to generate two molecules of hydroxyl radicals (Scheme 2). One hydroxyl radical undergoes single-electron transfer (SET) with TEMPOH to form the TEMPO radical, while the molybdenum-based catalyst combines with another hydroxyl radical to generate a [Mo-OH] species. Subsequently, the TEMPO radical mediates the radical oxidation of the substrate **1**, yielding a radical intermediate **A**. Finally, this radical intermediate further undergoes single-electron transfer (SET) with the [Mo-OH] species, affording the target pyridines **2** and simultaneously regenerating the initial molybdenum-based catalyst (*n*-Bu_4_N)_2_[Mo_6_O_19_]. During the reaction, the change in color from yellow to blue and back to yellow confirmed the possibility of the catalyst cycle.

## 3. Materials and Methods

### 3.1. Chemicals and Instruments

Unless otherwise noted, all commercially available materials and solvents were used directly without further purification. The substances **1a**, **1b**, **1w**, **1y**, **1z**, and **1aa** were purchased from Shanghai Aladdin Biochemical Technology Co., Ltd. (Shanghai, China).

All ^1^H NMR and ^13^C NMR data were recorded with a Bruker spectrometer (Billerica, MA, USA, 400 MHz for ^1^H NMR and 101 MHz for ^13^C NMR) using TMS as internal standard and reported relative to residual solvent signals as follows: chemical shift (δ ppm), multiplicity, coupling constant (Hz), and integration. The multiplicities are denoted as follows: s, singlet; d, doublet; t, triplet; q, quartet; m, multiplet; br, broad. Column chromatography was performed using 200–300 mesh silica gel. The mass spectra were determined on a Waters Xevo TQ-XS. HPLC-MS spectra were recorded on Agilent 1290II-6560 (Agilent, Milford, MA, USA). Melting point was tested by a WRX-4 microscopic melting point apparatus from Shanghai Yice Instrument and Equipment Co., Ltd. (Shanghai, China).

### 3.2. Preparation of Lindqvist-Type Catalyst (n-Bu_4_N)_2_[Mo_6_O_19_]

Na_2_MoO_4_·2H_2_O (5.0 g, 20.7 mmol) was added into purified water (15 mL) under stirring at room temperature. Next, the solution was acidified with 8% HCl (3.8 mL, 31 mmol), maintaining the pH of 4 and stirring at room temperature for 3 min. While stirring thoroughly, tetrabutylammonium bromide (3.27 g, 10.1 mmol) was added. After a white precipitate formed in the reaction mixture, it was heated to 70 °C and maintained for 40 min. During this process, the white precipitate gradually turned into a yellow precipitate. After the reaction was complete, vacuum filtration was performed, followed by washing with water (40 mL) to obtain the crude product (4.34 g).

The crude product was dissolved in acetone (50 mL) and heated to 65 °C while stirring. It was then cooled to room temperature, filtered to obtain the product, and dried in an oven for 12 h. The final product was obtained as a yellow solid (3.96 g, 83.5% yield). The X-ray crystal was obtained according to the method in the Supplementary Information, as well as the structural confirmation.

### 3.3. Preparation of 1,4-DHPs

The substrates were purchased (see the Supplementary Information file) or prepared according to the following procedure.

Aldehyde (1–2 g, 1.0 equiv.) was added into ethanol (5 V) while stirring at room temperature; ethyl acetoacetate (2.2 equiv.) and 3 mL of 30% ammonia solution (1.6 equiv.) were added sequentially. After adding the 30% ammonia solution, the mixture turned yellow and clear immediately. Then the mixture was heated to 80 °C for refluxing and stirred for 3 h. When the yellow color gradually deepened, the reaction was monitored by TLC (petroleum ether/ethyl acetate = 5:1, 254 nm).

After the reaction was reduced to room temperature, it was extracted with dichloromethane or ethyl acetate and water. The resulting organic phase was washed with water and sodium chloride solution, dried over anhydrous Na_2_SO_4_, and concentrated to obtain the crude product. Then the purification was conducted by column chromatography to obtain the product.

### 3.4. Aromatization of 1,4-Dihydropyridines

Unless otherwise mentioned, the general procedure was as follows.

1,4-Dihydropyridines (0.5 mmol, 1.0 equiv.) was added into the mixture of the catalyst (*n*-Bu_4_N)_2_[Mo_6_O_19_] (0.02 equiv.) in acetonitrile (2 mL for 0.5 mmol 1,4-dihydropyridine). Then, 30% H_2_O_2_ (0.4~0.5 equiv.*3 in three batches, with a 20 min interval during each addition period) was added into the reaction mixture. After the addition was finished, the reaction was heated to 60 °C and monitored by TLC (from petroleum ether to petroleum ether/ethyl acetate = 5:1, 254 nm).

Ethyl acetate was used for extraction and concentrated to obtain the crude product. Then the purification was performed by column chromatography to afford the aromatization product.

## 4. Conclusions

We developed a selective oxidation of Hantzsch 1,4-dihydropyridines (1,4-DHPs) to prepare pyridines via a Lindqvist-type catalyst (*n*-Bu_4_N)_2_[Mo_6_O_19_] and TEMPO as the co-catalyst. The reaction under the ternary POM/H_2_O_2_/TEMPO catalytic system exhibited excellent conversion and high regioselectivity for both electron-rich and electron-deficient 1,4-DHPs, affording poly-substituted pyridines in high yields of 85.3–98.5% (except for **2d** due to the radical cleavage) through a radical oxidation mechanism; furthermore, the catalyst maintained significant activity and could continue to catalyze the reaction effectively after six cycles. The convenient preparation of the pyridines with the stability and compatibility of the aromatization demonstrates their potential in medicinal chemistry.

## Figures and Tables

**Table 1:** Screenings of the ternary system for aromatization of **1a** ^[a]^.

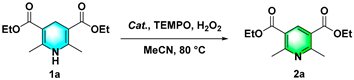					
Entry	Cat. (n-Bu_4_N)_2_[Mo_6_O_19_]	H_2_O_2_	TEMPO	Time (h)	Yield of 2a (%) ^[b]^
1	0.02	1.2	-	1	60
2	0.02	-	-	24	N.D. ^[c]^
3	-	1.2	-	10	85
4	0.02	1.2	1.0	1.5	95
5	-	1.2	1.0	5	90
6	-	-	1.0	24	86 ^[d]^
7	0.02	-	1.0	20	91

Notes: ^[a]^ The transformation of **1a** (0.5 mmol) was performed under the standard conditions using as a certain equivalent amount of Lindqvist-type catalyst (n-Bu_4_N)_2_[Mo_6_O_19_], H_2_O_2_ as oxidant and TEMPO as additive in MeCN at 80 °C. ^[b]^ Isolated yield. ^[c]^ Not detected with trace conversion rate by TLC. ^[d]^ About 90% of conversion rate was observed by TLC.

**Table 2:** Optimization of the reaction conditions ^[a]^.

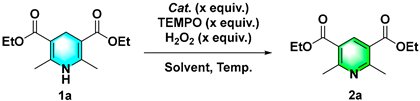							
Entry	Catalyst	Oxidant	TEMPO (Equiv.)	Solvent	Temp. (°C)	Time (h)	Yield (%) ^[b]^
**1**	(n-Bu_4_N)_2_[Mo_6_O_19_]	H_2_O_2_	0.5	MeCN	80	1.5	92.7
**2**	(n-Bu_4_N)_2_[Mo_6_O_19_]	H_2_O_2_	0.1	MeCN	80	1.5	93.5
**3**	-	10% NaClO	0.1	DCM	r.t.	5	42.7 ^[c]^
**4**	Fe(NO_3_)_3_	Air	0.1	DCE	60	24	trace ^[d]^
**5**	Fe(NO_3_)_3_	H_2_O_2_	0.1	DCE	60	16	<20.0 ^[d]^
**6**	Na_2_MoO_4_	H_2_O_2_	0.1	MeCN	80	15	<20.0 ^[d]^
**7**	(n-Bu_4_N)_2_[Mo_6_O_19_]	H_2_O_2_ ^[e]^	0.1	MeCN	80	1	82.3
**8**	(n-Bu_4_N)_2_[Mo_6_O_19_]	H_2_O_2_ ^[f]^	0.1	MeCN	80	1	84.1
**9**	(n-Bu_4_N)_2_[Mo_6_O_19_]	H_2_O_2_ ^[g]^	0.1	MeCN	80	1	96.6
**10**	(n-Bu_4_N)_2_[Mo_6_O_19_]	H_2_O_2_	0.1	MeCN	60	1	96.8
**11**	(n-Bu_4_N)_2_[Mo_6_O_19_]	H_2_O_2_	0.1	MeCN	40	1	87.1
**12**	(n-Bu_4_N)_2_[Mo_6_O_19_]	H_2_O_2_	0.1	MeCN	r.t.	18	84.7
**13**	(n-Bu_4_N)_2_[Mo_6_O_19_]	H_2_O_2_	0.1	HFIP	60	1	85.5
**14**	(n-Bu_4_N)_2_[Mo_6_O_19_]	H_2_O_2_	0.1	MeOH	60	1	64.5
**15**	(n-Bu_4_N)_2_[Mo_6_O_19_]	H_2_O_2_	0.1	EtOAc	60	2	83.2
**16**	(n-Bu_4_N)_2_[Mo_6_O_19_]	H_2_O_2_	0.1	THF	60	2	82.3
**17**	(n-Bu_4_N)_2_[Mo_6_O_19_]	H_2_O_2_	TEMPOL	MeCN	60	1	94.3
**18**	(n-Bu_4_N)_2_[Mo_6_O_19_]	H_2_O_2_	TEMP	MeCN	60	2	66.4
**19**	(n-Bu_4_N)_2_[Mo_6_O_19_]	H_2_O_2_	TEMPOH	MeCN	60	2	74.4

Notes: ^[a]^ Unless otherwise mentioned, the reaction was performed under the following general conditions. **1a** (0.5 mmol, 1 equiv.) was mixed with (*n*-Bu_4_N)_2_[Mo_6_O_19_] (0.02 equiv.) and H_2_O_2_ (1.5 equiv.) in MeCN (2 mL). The reaction was heated or stirred at r.t. for a certain period of time and monitored by TLC. ^[b]^ Isolated yield. ^[c]^ The conversion rate was about 60% by TLC. ^[d]^ The conversion rate was below 20% by TLC. ^[e]^ 0.8 equiv. of H_2_O_2_; ^[f]^ 1.0 equiv. of H_2_O_2_; ^[g]^ 1.2 equiv. H_2_O_2_ was added to the reaction system in three batches, with a 20 min interval during the additions.

## Data Availability

The original contributions presented in this study are included in the article/Supplementary Material. Further inquiries can be directed to the corresponding authors.

## Supplementary Materials

The following supporting information can be downloaded at: https://www.mdpi.com/article/10.3390/molecules31193529/s1, Optimization of the reaction conditions, structural characterization data and NMR spectra are available in the Supporting Information.

## Abbreviations

The following abbreviations are used in this manuscript.

TEMPO	2,2,6,6-tetramethylpiperidin-1-oxyl
1,4-DHPs	1,4-dihydropyridines
POM	polyoxometalate
MeCN	acetonitrile
HFIP	hexafluoroisopropanol
TEMPOL	4-hydroxy-2,2,6,6-tetramethyl-1-piperidinyloxy
TEMP	2,2,6,6-tetramethylpiperidine
TEMPOH	1-hydroxy-2,2,6,6-tetramethylpiperidine
