# Supplementary material for: A Green Lindqvist-Type POM/TEMPO/H2O2 Ternary Catalytic System for the Selective Oxidation of Hantzsch 1,4-Dihydropyridines (1,4-DHPs) to Pyridines
Source: Molecules. 2026 Oct 3;31(19):3529. doi: 10.3390/molecules31193529 (PMC13667240; doi:10.3390/molecules31193529)
Supplement: Supplementary file 1 [file molecules-31-03529-s001.zip › molecules-4491634-supplementary.pdf]

Supplementary Information

A green Lindqvist-type POM/TEMPO/H_2O_2 ternary catalytic system for the selective aromatization of Hantzsch 1,4-dihydropyridines (1,4-DHPs) to pyridines

Qiang Zeng†, Xiaoxiao Yan†, Shaohong Hou, Yutong Zhang, Jiuzhong Huang, Gaorong Wu, Dan Miao, Feng Jiang,* and Weiming Chen*

School of Pharmacy, Gannan Medical University, Ganzhou 341000, Jiangxi, China. E-mail: 8870974124@163.com (Q. Z.)； yanxx2025@gmu.edu.cn (X.Y.)

* Correspondence: jiangfenghz@163.com (F. J.); wader_chen@163.com (W. C).

† These authors contributed equally to this work.

Supplementary Information (SI) available: experimental conditions, supplementary table and NMR spectra.

Contents

S1 General information……………………………………………………………………………………………...3

S2. Information of Lindqvist-type catalyst (*n*-$Bu_4N)_2[Mo_6O_{19}]$ ……………………………………………………3

S3. Reactions screening experiments…………………………………………………………………………………………4

S4. Scale-up experiments and catalyst recovered experiments……………………………………………………………..5

S5. NMR spectra of substrates (1,4-DHPs) and products (pyridines)…………………………………………………………6

S6. References………...64

S1. General information

All commercially available materials and solvents were used directly without further purification unless otherwise noted. ^{1}H NMR and ^{13}C NMR data were recorded with a Bruker spectrometer (400 MHz for ^{1}H NMR and 101 MHz for ^{13}C NMR) using TMS as internal standard and reported relative to residual solvent signals as follows: chemical shift (δ ppm), multiplicity, coupling constant (Hz), and integration. The multiplicities are denoted as follows: s, singlet; d, doublet; t, triplet; q, quartet; m, multiplet; br, broad. The mass spectra were determined on a Waters Xevo TQ-XS. HPLC-MS spectra were recorded on Agilent 1290II-6560. Melting point was tested by a WRX-4 microscopic melting point apparatus from *Shanghai Yice Instrument and Equipment Co., Ltd*.. Column chromatography was performed using 200-300 mesh silica gel.

The substances **1a**, **1b**, **1w**, **1y**, **1z**, and **1aa** were purchased from *Shanghai Aladdin Biochemical Technology Co., Ltd.*.

S2. Information of Lindqvist-type catalyst $(n\text{-}Bu_4N)_2[Mo_6O_{19}]$

Single crystals of the catalyst were obtained by evaporation spontaneously in CH_3CN. A suitable crystal was selected and measured on a SuperNova, Dual, Cu at zero, AtlasS2 diffractometer. The crystal was kept at 250 K during data collection. Using Olex2, the structure was solved with the SHELXT structure solution program using Intrinsic Phasing and refined with the SHELXL refinement package using Least Squares minimisation.

Crystal Data for $C_{32}H_{72}Mo_6N_2O_{19}$ (M =1364.55 g/mol): monoclinic, space group C2/c (no. 15), a = 16.2042(8) A, b = 17.2492(8) A, c = 17.6639(9) A, β = 101.565(5)°, V = 4837.0(4) A^3, Z = 4, T = 250.00(10) K, μ(Mo Kα) = 1.577 mm-1, $Dcalc$ = 1.874 g/cm^3, 12658 reflections measured (3.908° ≤ 2Θ ≤ 58.86°), 5726 unique (R_{int} = 0.0292, R_{sigma} = 0.0417) which were used in all calculations. The final R_1 was 0.0356 (I > 2σ(I)) and wR_2 was 0.0851 (all data).

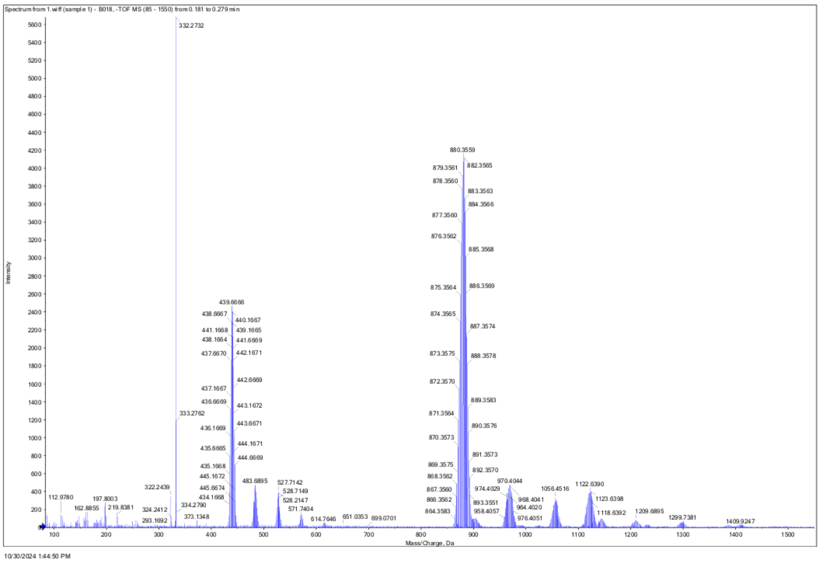

Figure S1. TOF MS of $(n\text{-}Bu_4N)_2[Mo_6O_{19}]$, ($CH_3CN$, m/z = 100-1500): 1122.6390 (calc. 1121.6223, $[Bu_4N][Mo_6O_{19}]^-$), 880.3539 (calc. 879.3375, $[Mo_6O_{19}]^{2-}$), and 439.6666 (calc. 439.6688, 1/2 $[Mo_6O_{19}]^{2-}$).

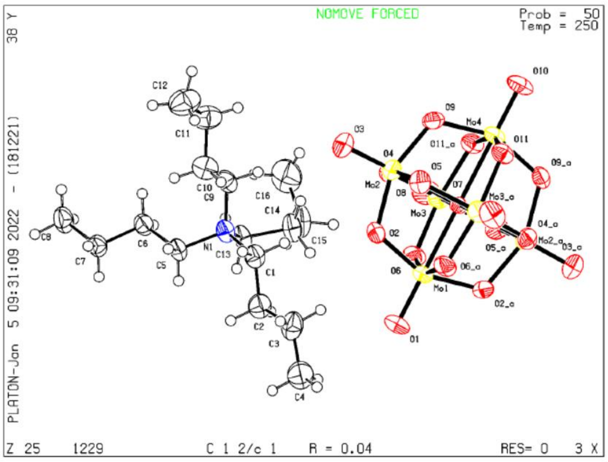

Figure S2. ORTEP plot of $(n\text{-}Bu_4N)_2[Mo_6O_{19}]$

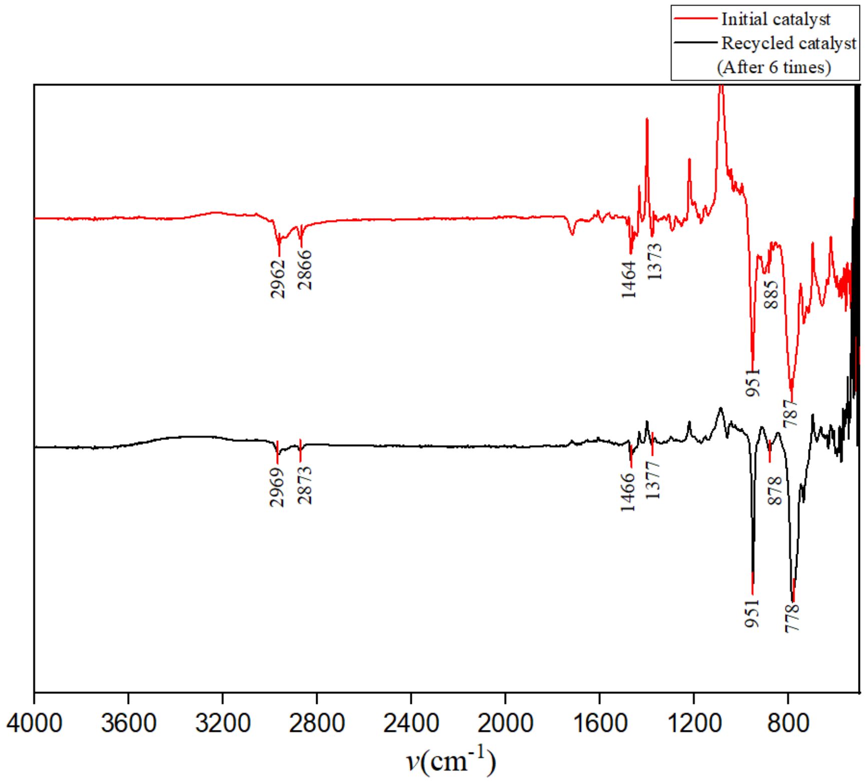

Figure S3. FT-IR spectra of (n-$Bu_4N)_2[Mo_6O_{19}]$

S3. Reactions screening experiments

As the amount of TEMPO was gradually decreased, the yield was not change significantly, so 0.1 equiv. of TEMPO was adopted for the aromatization.

Entry	TEMPO (equiv.)	Time (h)	Yield (%)
**1**	0.5	<1	92.7
**2**	0.1	<1	93.5

Compared with the catalyst (n-$Bu_4N)_2[Mo_6O_{19}]$, other catalytic oxidants indicated lower yields. For example, when the oxidant was 10% NaClO with NaCl as additive, the isolated yield was only 42.7%. When the catalyst was $Fe(NO_3)_3$ was used in air atmosphere, only trace product were observed by TLC. When the oxidant was hydrogen peroxide, regardless of the catalyst $Fe(NO_3)_3$ or Na_2MoO_4, only about one-fifth of the product was observed by TLC.

Entry	Catalyst	Oxidant	TEMPO (equiv.)	Solvent	Temp.(°C)	Time (h)	Yield (%)
**1**	-	10% NaClO	0.1	DCM	r.t.	5	42.7
**2**	$Fe(NO_3)_3$	Air	0.1	DCE	60	24	trace
**3**	$Fe(NO_3)_3$	H_2O_2	0.1	DCE	60	16	<20
**4**	Na_2MoO_4	H_2O_2	0.1	MeCN	80	15	<20

The yield was gradually increased when the amount of hydrogen peroxide was increased from 0.8 equiv. to 1.2 equiv..

Entry	Oxidant	Time (h)	Yield (%)
**1**	H_2O_2 (0.8 equiv.)	1	82.3
**2**	H_2O_2 (1.0 equiv.)	1	84.1
**3**	H_2O_2 (1.2 equiv.)	1	96.6

When the temperature was reduced to room temperature, the solubility of the substrate and the yield was decreased, respectively.

Entry	Temp. (°C)	Time (h)	Yield (%)
**1**	60	1	96.8
**2**	40	1	87.1
**3**	r.t.	18	84.7

When the reaction solvent was changed to HFIP, MeOH, EtOAc, and THF, all the yields were unsatisfactory.

Entry	Solvent	Time (h)	Yield (%)
**1**	HFIP	1	85.5
**2**	MeOH	1	64.5

**3**	EtOAc	2	83.2
**4**	THF	2	82.3

Except for TEMPOL, when TEMPO was changed to TEMP or TEMPOH, the yields were decreased.

Entry	TEMPO (equiv.)	Time (h)	Yield (%)
**1**	TEMPOL	1	94.3
**2**	TEMP	2	66.4
**3**	TEMPOH	2	74.4

S4. Scale-up experiment and recovered-catalyst experiments

Scale-up experiment was performed on a 10 g scale of diethyl 2,6-dimethyl-1,4-dihydropyridine-3,5-dicarboxylate **1a**.

Procedure: The substrate (about 10 g, 1.0 equiv.) was added into the mixture of catalyst $(n\text{-}Bu_4N)_2[Mo_6O_{19}]$ (0.02 equiv.) in acetonitrile (40 mL). 30% H_2O_2 (1.5 equiv. in three batches, with a 20-30 minutes interval between each**.**) was added into the reaction. After the addition was finished, the reaction was heated to 60 °C for 1.5 hours and monitored by TLC till the starting material was consumed completely.

After the aromatization was finished, the reaction solution was concentrated, extracted with ethyl acetate, and filtered to recover the catalyst. The filtrate was washed with water and sodium chloride solution, the organic phase was concentrated. Then the product was obtained by column chromatography. After recovered for 6 times, the isolated yields of product and the recycled rate of the catalyst were listed in the following table.

	Isolated yield	Recycled rate of the catalyst (based on the initial quantity)
Initial exp.	95.1%	-
1st cycle	95.3%	87.0%
2nd cycle	93.9%	87.0%
3rd cycle	95.1%	84.9%
4th cycle	94.3%	82.2%
5th cycle	92.9%	81.8%
6th cycle	92.3%	78.8%

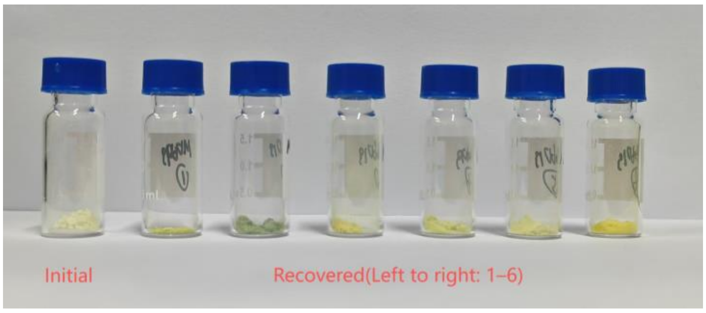

Figure S4. Appearances of the catalyst $(n\text{-}Bu_4N)_2[Mo_6O_{19}]$ (initial and recovered).

S5. NMR spectra of substrates (1,4-DHPs) and products (pyridines)

S5.1 NMR spectra of the substrates

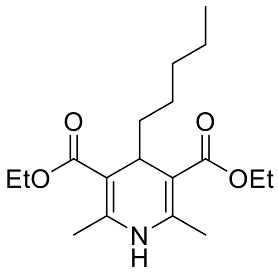

Compound **1c** (yellow solid, yield 53%). ^{1}H NMR (400 MHz, $CDCl_3$) δ 5.58 (s, 1H), 4.17 (dd, *J* = 17.3, 7.1 Hz, 4H), 3.91 (t, *J* = 5.8 Hz, 1H), 2.27 (s, 6H), 1.29 (t, *J* = 7.1 Hz, 10H), 1.21 – 1.16 (m, 4H), 0.83 (t, *J* = 6.9 Hz, 3H). ^{13}C NMR (101 MHz, $CDCl_3$) δ168.35, 144.85, 103.40, 59.68, 36.98, 32.97, 32.21, 24.68, 22.86, 19.53, 14.51, 14.22. M.p. 71.8-73.2 ℃ (lit. [1] 73-74 °C).

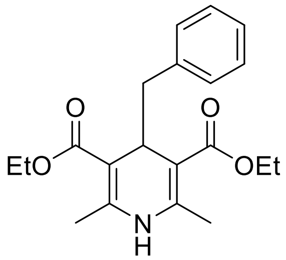

Compound **1d** (white solid, yield 64%). ^{1}H NMR (400 MHz, $CDCl_3$) δ 7.19 – 7.11 (m, 3H), 7.01 (d, *J* = 6.2 Hz, 2H), 5.68 (s, 1H), 4.18 (t, *J* = 5.6 Hz, 1H), 4.03 (dd, *J* = 14.1, 7.1 Hz, 4H), 2.56 (d, *J* = 5.6 Hz, 2H), 2.17 (s, 6H), 1.22 (t, *J* = 7.1 Hz, 6H). ^{13}C NMR (101 MHz, $CDCl_3$) δ 167.98, 145.63, 139.39, 130.18, 127.37, 125.69, 101.84, 59.66, 35.56, 19.26, 14.43. M.p. 114.2-115.8 ℃ (lit.[2] 114-116 °C).

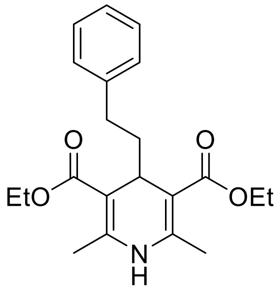

Compound **1e** (white solid, yield 51%). ^{1}H NMR (400 MHz, $CDCl_3$) δ 7.30 (d, J = 4.6 Hz, 1H), 7.27 (d, J = 6.3 Hz, 2H), 7.18 (d, J = 7.3 Hz, 2H), 5.66 (s, 1H), 4.24 (dd, J = 12.2, 6.6 Hz, 4H), 4.11 (t, J = 5.8 Hz, 1H), 2.63 – 2.55 (m, 2H), 2.35 (s, 6H), 1.75 – 1.67 (m, 2H), 1.34 (t, J = 7.1 Hz, 6H). ^{13}C NMR (101 MHz, $CDCl_3$) δ 167.99, 144.99, 143.14, 128.25, 128.15, 125.41, 102.98, 59.69, 38.40, 33.23, 31.34, 19.56, 14.43. M.p. 116.0-116.3 ℃ (lit. [3] 116 °C).

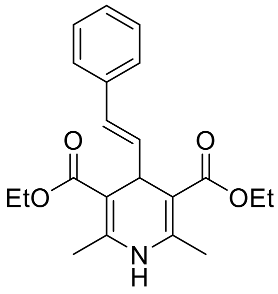

Compound **1f** (yellow solid, yield 47%). ^{1}H NMR (400 MHz, $CDCl_3$) δ 7.34 (d, J = 6.9 Hz, 2H), 7.29 (t, J = 3.7 Hz, 2H), 7.19 (d, J = 7.2 Hz, 1H), 6.24 (s, 1H), 6.18 (d, J = 6.4 Hz, 1H), 5.68 (s, 1H), 4.65 (d, J = 6.3 Hz, 1H), 4.21 (dd, J = 13.9, 7.0 Hz, 4H), 2.35 (s, 6H), 1.32 (t, J = 7.1 Hz, 6H). ^{13}C NMR (101 MHz, $CDCl_3$) δ 167.60, 144.83, 137.81, 131.80, 128.38, 128.08, 126.87, 126.25, 101.57, 59.79, 36.53, 19.58, 14.45. M.p. 143.6-143.8 ℃ (lit. [4] 144-146 °C).

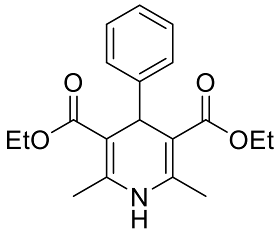

Compound **1g** (white solid, yield 70%). ^{1}H NMR (400 MHz, $CDCl_3$) δ 7.28 (d, J = 6.8 Hz, 2H), 7.20 (t, J = 7.4 Hz, 2H), 7.12 (t, J = 7.3 Hz, 1H), 5.63 (s, 1H), 4.98 (s, 1H), 4.08 (dd, J = 7.1, 4.9 Hz, 4H), 2.33 (s, 6H), 1.22 (t, J = 7.1 Hz, 6H). ^{13}C NMR (101 MHz, $CDCl_3$) δ 167.68, 147.79, 143.91, 128.02, 127.84, 126.11, 104.15, 59.75, 39.64, 19.59, 14.27. M.p. 152.2-152.4 °C (lit. [5] 150-152 °C).

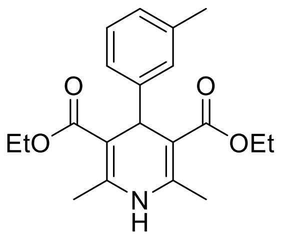

Compound **1h** (yellow solid, yield 66%). ^{1}H NMR (400 MHz, $CDCl_3$) δ7.08 (d, J = 6.7 Hz, 3H), 6.93 (d, J = 6.4 Hz, 1H), 5.79 (s, 1H), 4.96 (s, 1H), 4.09 (dd, J = 9.3, 7.1 Hz, 4H), 2.31 (s, 6H), 2.28 (s, 3H), 1.23 (t, J = 7.1 Hz, 6H). ^{13}C NMR (101 MHz, $CDCl_3$) δ 167.78, 147.73, 143.94, 137.17, 128.86, 127.76, 126.93, 125.11, 104.17, 59.75, 39.60, 21.61, 19.59, 14.30. M.p. 134. 6-135. 2 °C (lit. [6] 135 °C).

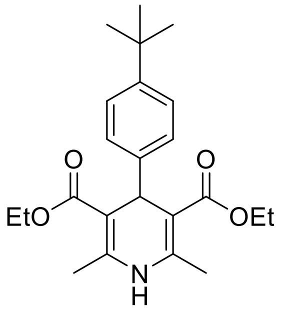

Compound **1i** (yellow solid, yield 65%). ^{1}H NMR (400 MHz, $CDCl_3$) δ 7.19 (d, J = 3.3 Hz, 4H), 5.67 (s, 1H), 4.96 (s, 1H), 4.09 (dd, J = 7.1, 5.7 Hz, 4H), 2.32 (s, 6H), 1.26 (s, 9H), 1.22 (t, J = 7.1 Hz, 6H). ^{13}C NMR (101 MHz, $CDCl_3$) δ 167.75, 148.60, 144.63, 143.72, 127.47, 124.73, 104.30, 59.72, 38.92, 34.31, 31.41, 19.64, 14.28. M.p. 96.3-97.2 °C (lit. [7] 95 °C).

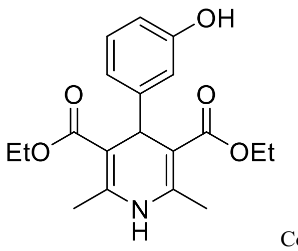

Compound **1j** (white solid, yield 47%). ^{1}H NMR (400 MHz, DMSO-d_6) δ 9.11 (s, 1H), 8.77 (s, 1H), 6.96 (t, J = 7.8 Hz, 1H), 6.58 (d, J = 7.1 Hz, 2H), 6.48 (d, J = 7.7 Hz, 1H), 4.80 (s, 1H), 3.99 (dd, J = 7.1, 5.3 Hz, 4H), 2.24 (s, 6H), 1.14 (t, J = 7.1 Hz, 6H). ^{13}C NMR (101 MHz, DMSO-d_6) δ 167.53, 157.38, 149.88, 145.60, 129.08, 118.50, 114.81, 113.28, 102.24, 59.47, 40.31, 18.68, 14.65. M.p. 186.3-186.7 °C (lit. [8] 186 °C).

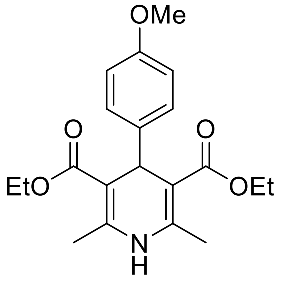

Compound **1k** (yellow solid, yield 63%). ^{1}H NMR (400 MHz, $CDCl_3$) δ 7.19 (d, J = 8.7 Hz, 2H), 6.74 (d, J = 8.7 Hz, 2H), 6.09 (s, 1H), 4.93 (s, 1H), 4.08 (dd, J = 7.1, 5.8 Hz, 4H), 3.73 (s, 3H), 2.28 (s, 6H), 1.22 (t, J = 7.1 Hz, 6H). ^{13}C NMR (101 MHz, $CDCl_3$) δ 167.73, 157.87, 143.61, 140.35, 128.98, 113.18, 104.38, 59.73, 55.15, 38.74, 19.61, 14.30. M.p. 152.9-153.7 °C (lit. [9] 152-154 °C).

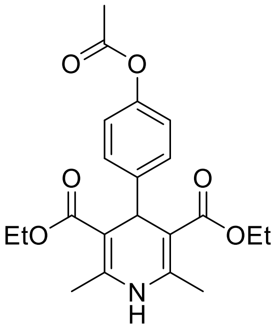

Compound **1l** (yellow solid, yield 50%). ^{1}H NMR (400 MHz, $CDCl_3$) δ 7.27 (d, *J* = 7.7 Hz, 2H), 6.91 (d, *J* = 8.4 Hz, 2H), 5.85 (s, 1H), 4.99 (s, 1H), 4.13 – 4.04 (m, 4H), 2.30 (s, 6H), 2.27 (s, 3H), 1.22 (t, *J* = 7.1 Hz, 6H). ^{13}C NMR (101 MHz, $CDCl_3$) δ 167.30, 148.77, 144.38, 131.53, 128.37, 124.99, 122.93, 103.70, 59.88, 39.90, 19.57, 14.12. M.p. 153.8-155.7 ℃.

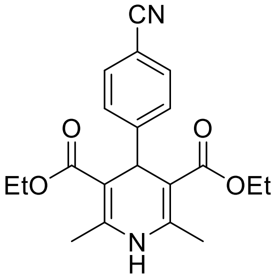

Compound **1m** (yellow solid, yield 53%). ^{1}H NMR (400 MHz, $CDCl_3$) δ 7.50 (d, *J* = 8.4 Hz, 2H), 7.38 (d, *J* = 8.3 Hz, 2H), 5.84 (s, 1H), 5.02 (s, 1H), 4.07 (dd, *J* = 7.1, 4.7 Hz, 4H), 2.33 (s, 6H), 1.20 (t, *J* = 7.1 Hz, 6H). ^{13}C NMR (101 MHz, CDCl3*)* δ 167.17, 153.11, 144.65, 131.84, 128.89, 119.34, 109.70, 103.21, 59.97, 40.26, 19.60, 14.27. M.p. 160.7-162.1 ℃ (lit. [10] 162-166 °C).

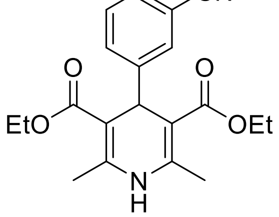

Compound **1n** (yellow solid, yield 50%). ^{1}H NMR (400 MHz, $CDCl_3$) δ 7.54 (d, *J* = 8.6 Hz, 2H), 7.42 (d, *J* = 7.8 Hz, 1H), 7.30 (t, *J* = 7.5 Hz, 1H), 5.86 (s, 1H), 4.99 (s, 1H), 4.08 (t, *J* = 7.1 Hz, 4H), 2.34 (s, 6H), 1.21 (t, *J* = 7.1 Hz, 6H). ^{13}C NMR (101 MHz, $CDCl_3$) δ 167.14, 149.24, 144.56, 132.86, 131.94, 129.87, 128.52, 119.46, 111.81, 103.39, 59.97, 39.81, 19.66, 14.28. M.p. 145.9-147.2 ℃ (lit. [11] 144-146 °C).

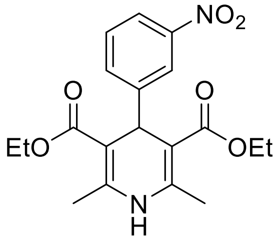

Compound **1o** (yellow solid, yield 46%). ^{1}H NMR (400 MHz, $CDCl_3$) δ 8.12 (s, 1H), 8.00 (d, *J* = 8.2 Hz, 1H), 7.63 (d, *J* = 6.3 Hz, 1H), 7.37 (t, *J* = 7.9 Hz, 1H), 5.85 (s, 1H), 5.08 (s, 1H), 4.09 (d, *J* = 7.1 Hz, 4H), 2.36 (s, 6H), 1.21 (t, *J* = 7.1 Hz, 6H). ^{13}C NMR (101 MHz, $CDCl_3$) δ 167.11, 149.91, 148.16, 144.68, 134.54, 128.59, 123.14, 121.35, 103.37, 60.01, 39.97, 19.67, 14.24. M.p. 159.1-160.5 ℃ (lit. [12] 162-164 °C).

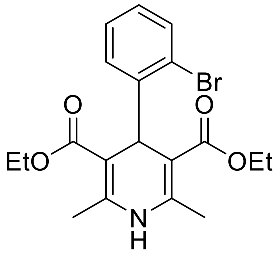

Compound **1p** (yellow solid, yield 56%). ^{1}H NMR (400 MHz, $CDCl_3$) δ 7.42 (d, *J* = 6.6 Hz, 1H), 7.38 (d, *J* = 7.8 Hz, 1H), 7.16 (t, *J* = 6.8 Hz, 1H), 7.01 – 6.90 (m, 1H), 5.64 (s, 1H), 5.35 (s, 1H), 4.10 (dd, *J* = 7.1, 3.9 Hz, 4H), 2.30 (s, 6H), 1.20 (t, *J* = 7.1 Hz, 6H). ^{13}C NMR (101 MHz, $CDCl_3$) δ 167.72, 147.47, 143.64, 132.74, 131.71, 127.62, 127.48, 122.71, 104.29, 59.79, 39.82, 19.62, 14.49. M.p. 135.9-136.6 ℃ (lit. [13] 131-134 °C).

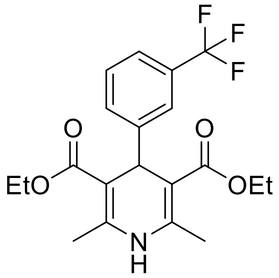

Compound **1q** (yellow solid, yield 52%). ^{1}H NMR (400 MHz, $CDCl_3$) δ 7.52 (s, 1H), 7.46 (d, *J* = 7.6 Hz, 1H), 7.38 (d, *J* = 7.7 Hz, 1H), 7.31 (t, J = 7.7 Hz, 1H), 5.82 (s, 1H), 5.03 (s, 1H), 4.07 (dd, *J* = 13.4, 6.9 Hz, 4H), 2.33 (s, 6H), 1.20 (t, *J* = 7.1 Hz, 6H). ^{13}C NMR (101 MHz, $CDCl_3$) δ 167.29, 148.76, 144.35, 131.53, 129.82 (d, *J* = 32.0 Hz), 128.37, 125.76, 125.01 (d, *J* = 4.0 Hz), 122.95 (d, *J* = 4.0 Hz), 103.72, 59.88, 39.90, 19.58, 14.12. M.p. 126.8-127.6 °C (lit. [14] 124-128 °C).

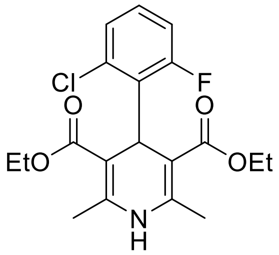

Compound **1r** (yellow solid, yield 55%). ^{1}H NMR (400 MHz, $CDCl_3$) δ 7.09 (d, *J* = 7.8 Hz, 1H), 7.03 (d, *J* = 5.5 Hz, 1H), 6.86 (ddd, J = 10.4, 8.0, 1.5 Hz, 1H), 5.78 (s, 1H), 5.65 (s, 1H), 4.03 (q, *J* = 7.1 Hz, 4H), 2.26 (s, 6H), 1.15 (t, *J* = 7.1 Hz, 6H). ^{13}C NMR (101 MHz, $CDCl_3$) δ 167.67, 162.64 (d, *J* = 251.3 Hz), 145.34, 135.54, 127.50 (d, *J* = 10.3 Hz), 125.81 (d, *J* = 3.3 Hz), 113.99 (d, *J* = 24.4 Hz), 100.14, 59.65, 33.72, 19.62, 14.15. M.p. 134.4-135.7 °C.

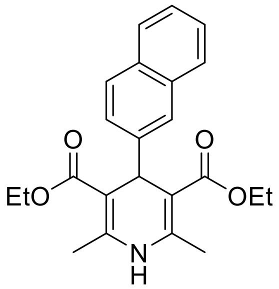

Compound **1s** (yellow solid, yield 62%). ^{1}H NMR (400 MHz, $CDCl_3$) δ 7.75 (d, *J* = 6.8 Hz, 2H), 7.71 – 7.64 (m, 2H), 7.48 (d, *J* = 8.5 Hz, 1H), 7.39 (t, *J* = 5.4 Hz, 2H), 5.62 (s, 1H), 5.16 (s, 1H), 4.15 – 3.99 (m, 4H), 2.37 (s, 6H), 1.21 (t, *J* = 7.1 Hz, 6H). ^{13}C NMR (101 MHz, $CDCl_3$) δ 167.65, 145.22, 143.92, 133.36, 132.31, 127.87, 127.49, 127.43, 127.14, 126.30, 125.54, 125.11, 104.09, 59.78, 39.91, 19.69, 14.30. M.p. 138.6-140.8 °C (lit. [15] 137-139 °C).

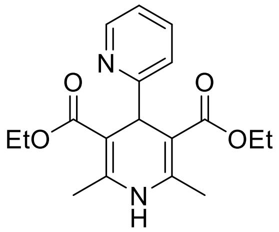

Compound **1t** (brown solid, yield 46%). ^{1}H NMR (400 MHz, $CDCl_3$) δ 9.07 (s, 1H), 8.48 (d, *J* = 4.6 Hz, 1H), 7.59 (t, *J* = 7.6 Hz, 1H), 7.41 (d, *J* = 8.8 Hz, 1H), 7.18 – 7.08 (m, 1H), 5.18 (s, 1H), 4.05 (q, *J* = 7.2 Hz, 4H), 2.23 (s, 6H), 1.20 (d, *J* = 7.1 Hz, 6H). ^{13}C NMR (101 MHz, $CDCl_3$) δ 167.78, 165.30, 148.05, 146.70, 135.73, 124.54, 121.91, 101.57, 59.52, 42.92, 18.63, 14.30. M.p. 192.0-193.0 °C (lit. [16] 193-195 °C).

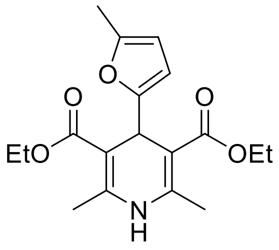

Compound **1u** (yellow solid, yield 42%). ^{1}H NMR (400 MHz, $CDCl_3$) δ 5.77 (s, 2H), 5.70 (s, 1H), 5.12 (s, 1H), 4.25 – 4.09 (m, 4H), 2.32 (s, 6H), 2.18 (s, 3H), 1.27 (t, *J* = 7.1 Hz, 6H). ^{13}C NMR (101 MHz, $CDCl_3$) δ 167.65, 157.00, 150.28, 144.91, 105.85, 105.02, 100.90, 59.75, 33.34, 19.51, 14.36, 13.74. M.p. 140.9-142.8 °C (lit. [17] 142 °C).

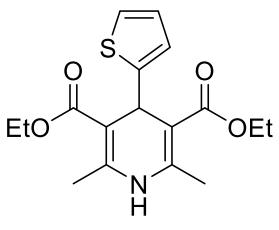

Compound **1v** (yellow solid, yield 44%). ^{1}H NMR (400 MHz, $CDCl_3$) δ 7.05 (d, *J* = 5.1 Hz, 1H), 6.87 – 6.83 (m, 1H), 6.79 (d, *J* = 4.5 Hz, 1H), 5.89 (s, 1H), 5.34 (s, 1H), 4.27 – 4.05 (m, 4H), 2.33 (s, 6H), 1.27 (t, *J* = 7.1 Hz, 6H). ^{13}C NMR (101 MHz, $CDCl_3$) δ 167.34, 151.55, 144.45, 126.35, 123.18, 123.15, 103.63, 59.93, 34.39, 19.57, 14.34. M.p. 171.8-172.4 °C (lit. [18] 171-173 °C).

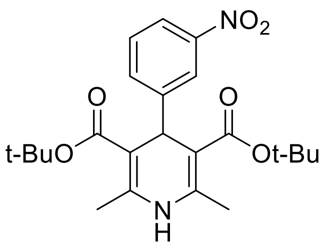

Compound **1x** (yellow solid, yield 45%). ^{1}H NMR (400 MHz, $CDCl_3$) δ 7.54 (d, *J* = 9.4 Hz, 2H), 7.42 (d, *J* = 7.3 Hz, 1H), 7.30 (t, *J* = 7.6 Hz, 1H), 5.57 (s, 1H), 4.92 (s, 1H), 2.30 (s, 6H), 1.39 (s, 18H). ^{13}C NMR (101 MHz, $CDCl_3$) δ 166.60, 149.43, 143.67, 132.81, 131.89, 129.70, 128.41, 119.51, 111.62, 104.48, 80.09, 40.30, 28.30, 19.55. M.p. 73.1-74.9 °C (lit. [14] 70-72 °C).

S5.2 NMR spectra of products

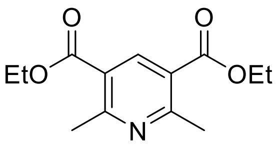

Compound **2a** (reaction time 1 h, white solid) in 96.8% yield**.** ^{1}H NMR (400 MHz, $CDCl_3$) δ 8.68 (s, 1H), 4.40 (q, *J* = 7.1 Hz, 4H), 2.85 (s, 6H), 1.41 (t, *J* = 7.1 Hz, 6H). ^{13}C NMR (101 MHz, $CDCl_3$) δ 165.98, 162.25, 140.94, 123.07, 61.43, 25.00, 14.29. M.p. 67.8-69.1 °C (lit. [19] 68-72 °C).

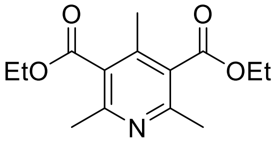

Compound **2b** (reaction time 4 h, yellow solid) in 98.5% yield**.** ^{1}H NMR (400 MHz, $CDCl_3$) δ 4.34 (q, *J* = 7.1 Hz, 4H), 2.45 (s, 6H), 2.21 (s, 3H), 1.32 (t, *J* = 7.1 Hz, 6H). ^{13}C NMR (101 MHz, $CDCl_3$) δ 168.32, 154.86, 141.99, 127.52, 61.53, 22.88, 16.90, 14.12. M.p. 144.8-146.9 °C (lit. [20] 140-145 °C).

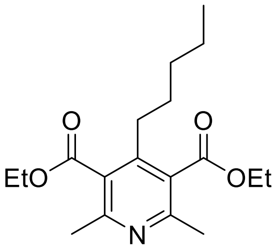

Compound **2c** (reaction time 4 h, colorless oil) in 88% yield**.** ^{1}H NMR (400 MHz, $CDCl_3$) δ 4.38 (q, *J* = 7.1 Hz, 4H), 2.58 – 2.51 (m, 2H), 2.48 (s, 6H), 1.52 (s, 2H), 1.37 (t, *J* = 7.1 Hz, 6H), 1.27 (s, 4H), 0.90 – 0.80 (m, 3H). ^{13}C NMR (101 MHz, $CDCl_3$) δ 168.54, 154.99, 146.56, 127.18, 61.55, 32.13, 31.41, 30.56, 22.93, 22.26, 14.15, 13.89.

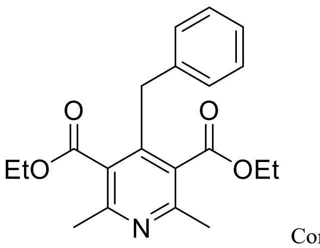

Compound **2d** (reaction time 6 h, colorless oil) in 53% yield along with **1a** (41%)**.** ^{1}H NMR (400 MHz, $CDCl_3$) δ 7.22 (d, *J* = 7.7 Hz, 3H), 7.07 (d, *J* = 7.1 Hz, 2H), 4.18 (q, *J* = 7.1 Hz, 4H), 4.04 (s, 2H), 2.52 (s, 6H), 1.17 (t, *J* = 7.1 Hz, 6H). ^{13}C NMR (101 MHz, $CDCl_3$) δ 168.31, 155.31, 144.62, 137.98, 129.09, 128.29, 127.87, 126.49, 61.58, 36.22, 23.03, 13.87.

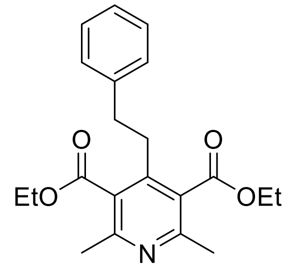

Compound **2e** (reaction time 3 h, white solid) in 93.8% yield. ^{1}H NMR (400 MHz, $CDCl_3$) δ 7.30 (t, *J* = 8.0 Hz, 2H), 7.22 (d, *J* = 7.4 Hz, 1H), 7.17 (d, *J* = 8.4 Hz, 2H), 4.43 (q, *J* = 7.8, 7.1 Hz, 4H), 2.86 (s, 4H), 2.54 (s, 6H), 1.39 (t, *J* = 7.2 Hz, 6H). ^{13}C NMR (101 MHz, $CDCl_3$) δ 168.43, 155.32, 145.52, 141.11, 128.59, 128.15, 127.27, 126.31, 61.77, 37.06, 33.85, 23.03, 14.23. M.p. 126.4-127.7 °C (lit. [21] 128 °C).

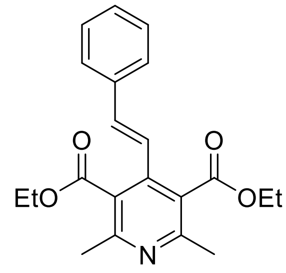

Compound **2f** (reaction time 1.5 h, colorless oil) in 96.6% yield. ^{1}H NMR (400 MHz, $CDCl_3$) δ 7.41 (d, *J* = 6.5 Hz, 2H), 7.39 – 7.28 (m, 3H), 7.10 (d, *J* = 15.6 Hz, 1H), 6.80 (d, *J* = 16.5 Hz, 1H), 4.33 (q, *J* = 7.1, 6.6 Hz, 4H), 2.57 (s, 6H), 1.32 – 1.24 (m, 6H). ^{13}C NMR (101 MHz, $CDCl_3$) δ 168.32, 155.58, 142.20, 136.62, 136.07, 128.81, 126.84, 125.67, 122.72, 61.72, 23.00, 14.20.

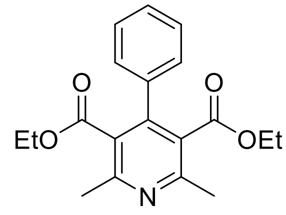

Compound **2g** (reaction time 3.5 h, white solid) in 95.1% yield. ^{1}H NMR (400 MHz, $CDCl_3$) δ 7.40 – 7.31 (m, 3H), 7.24 (d, *J* = 3.6 Hz, 2H), 4.00 (q, *J* = 7.1 Hz, 4H), 2.61 (s, 6H), 0.96 – 0.84 (m, 6H). ^{13}C NMR (101 MHz, $CDCl_3$) δ 167.87, 155.41, 146.13, 136.57, 128.42, 128.07, 126.92, 22.91, 13.54. M.p. 61.6-61.9 °C (lit. [22] 61-63 °C).

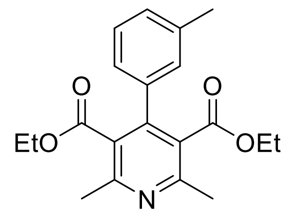

Compound **2h** (reaction time 3 h, colorless oil) in 98% yield. ^{1}H NMR (400 MHz, $CDCl_3$) δ 7.23 (t, *J* = 7.5 Hz, 1H), 7.16 (d, *J* = 7.5 Hz, 1H), 7.04 (d, *J* = 8.0 Hz, 2H), 4.01 (q, *J* = 7.1 Hz, 4H), 2.59 (s, 6H), 2.33 (s, 3H), 0.91 (t, J = 7.1 Hz, 6H). ^{13}C NMR (101 MHz, $CDCl_3$) δ 167.94, 155.32, 146.32, 137.74, 136.44, 129.14, 128.64, 128.05, 126.95, 125.17, 61.32, 22.89, 21.31, 13.57.

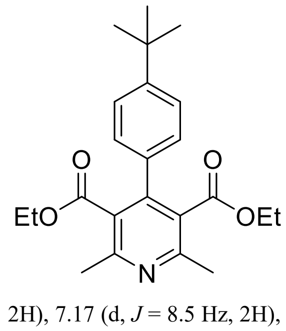

Compound **2i** (reaction time 4 h, white solid) in 95.3% yield. ^{1}H NMR (400 MHz, $CDCl_3$) δ 7.37 (d, *J* = 8.6 Hz, 2H), 7.17 (d, *J* = 8.5 Hz, 2H), 4.00 (q, *J* = 7.2 Hz, 4H), 2.60 (s, 6H), 1.31 (s, 9H), 0.83 (t, *J* = 7.1 Hz, 6H). ^{13}C NMR (101 MHz, $CDCl_3$) δ 168.01, 155.35, 151.57, 146.32, 133.65, 127.75, 127.02, 124.98, 61.25, 34.63, 31.28, 22.88, 13.40. M.p. 99.1-100.8 °C (lit. [23] 97-98 °C).

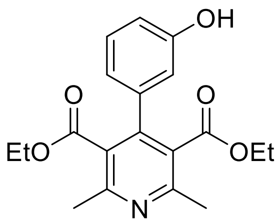

Compound **2j** (reaction time 4 h, colorless oil) in 86.5% yield. ^{1}H NMR (400 MHz, $CDCl_3$) δ 7.22 (d, *J* = 7.9 Hz, 1H), 6.85 (d, *J* = 5.7 Hz, 1H), 6.80 (d, *J* = 8.5 Hz, 1H), 6.72 (s, 1H), 5.41 (s, 1H), 4.05 (q, *J* = 7.1 Hz, 4H), 2.60 (s, 6H), 0.95 (t, *J* = 7.6 Hz, 6H). ^{13}C NMR (101 MHz, $CDCl_3$) δ 167.72, 156.25, 155.40, 146.39, 137.62, 129.61, 127.17, 119.88, 115.89, 114.93, 61.58, 22.40, 13.57.

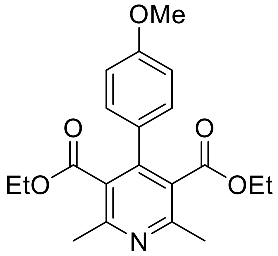

Compound **2k** (reaction time 2 h, white solid) in 95.5% yield. ^{1}H NMR (400 MHz, $CDCl_3$) δ 7.18 (d, *J* = 8.7 Hz, 2H), 6.89 (d, *J* = 8.7 Hz, 2H), 4.04 (q, *J* = 7.1 Hz, 4H), 3.81 (s, 3H), 2.58 (s, 6H), 0.98 (t, *J* = 7.1 Hz, 6H). ^{13}C NMR (101 MHz, $CDCl_3$) δ 168.09, 159.80, 155.16, 145.76, 129.41, 128.69, 127.25, 113.57, 55.31, 22.86, 13.74. M.p. 48.7-49.4 °C (lit. [24] 48-50 °C).

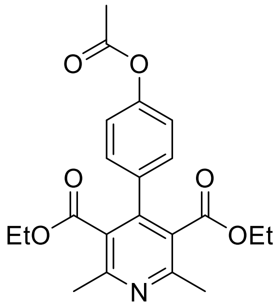

Compound **2l** (reaction time 1 h, yellow solid) in 90.6% yield. ^{1}H NMR (400 MHz, $CDCl_3$) δ 7.24 (d, J = 8.6 Hz, 2H), 7.07 (d, *J* = 8.6 Hz, 2H), 3.99 (q, *J* = 7.1 Hz, 4H), 2.57 (s, 6H), 2.27 (s, 3H), 0.91 (t, *J* = 7.1 Hz, 6H). ^{13}C NMR (101 MHz, $CDCl_3$) δ 169.02, 167.74, 155.57, 150.90, 145.24, 134.10, 129.21, 126.91, 121.42, 61.51, 22.89, 21.09, 13.54. M.p. 57.8-58.3 °C.

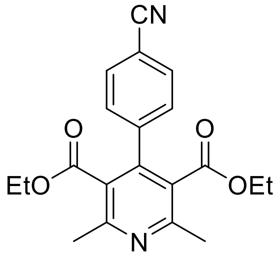

Compound **2m** (reaction time 4.5 h, white solid) in 89% yield. ^{1}H NMR (400 MHz, $CDCl_3$) δ 7.68 (d, J = 8.4 Hz, 2H), 7.38 (d, *J* = 8.4 Hz, 2H), 4.01 (q, *J* = 7.1 Hz, 4H), 2.61 (s, 6H), 0.94 (t, *J* = 7.1 Hz, 6H). ^{13}C NMR (101 MHz, $CDCl_3$) δ 167.19, 156.11, 144.24, 141.46, 129.11, 126.25, 118.27, 112.47, 61.64, 23.07. M.p. 103.6-104.4 °C (lit. [25] 101-103 °C).

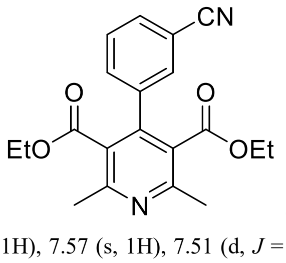

Compound **2n** (reaction time 5 h, white solid) in 91.4% yield. ^{1}H NMR (400 MHz, $CDCl_3$) δ 7.69 (d, *J* = 4.4 Hz, 1H), 7.57 (s, 1H), 7.51 (d, *J* = 6.6 Hz, 2H), 4.10 – 3.99 (m, 4H), 2.62 (s, 6H), 0.97 (t, *J* = 7.1 Hz, 6H). ^{13}C NMR (101 MHz, $CDCl_3$) δ 167.19, 156.05, 143.61, 137.93, 132.71, 132.02, 131.71, 129.04, 126.50, 118.05, 112.41, 61.62, 23.09, 13.72. M.p. 60.4-61.1 °C.

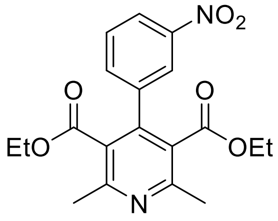

Compound **2o** (reaction time 3 h, white solid) in 91.4% yield**.** ^{1}H NMR (400 MHz, $CDCl_3$) δ 8.25 (d, J = 9.2 Hz, 1H), 8.18 (s, 1H), 7.58 (d, J = 7.5 Hz, 2H), 4.14 – 3.96 (m, 4H), 2.62 (s, 6H), 0.98 (t, J = 7.1 Hz, 6H). ^{13}C NMR (101 MHz, $CDCl_3$) δ 167.19, 156.10, 147.82, 143.52, 138.12, 134.39, 129.25, 126.59, 123.39, 123.35, 23.10, 13.73. M.p. 60.2-63.5 °C (lit. [26] 61-63 °C).

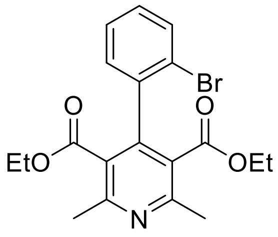

Compound **2p** (reaction time 2 h, colorless oil) in 97% yield**.** ^{1}H NMR (400 MHz, $CDCl_3$) δ 7.59 (d, J = 9.2 Hz, 1H), 7.34 – 7.28 (m, 1H), 7.25 – 7.20 (m, 1H), 7.17 (d, J = 5.7 Hz, 1H), 4.00 (d, J = 7.1, 1.6 Hz, 4H), 2.65 (s, 6H), 0.92 (t, J = 7.1 Hz, 6H). ^{13}C NMR (101 MHz, $CDCl_3$) δ 167.03, 156.48, 145.80, 137.72, 132.26, 130.40, 129.75, 126.66, 126.39, 122.72, 61.33, 23.41, 13.52.

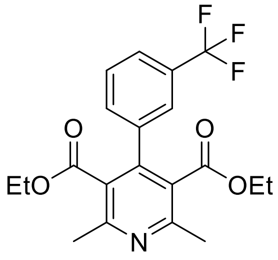

Compound **2q** (reaction time 8 h, white solid) in 85.3% yield**.** ^{1}H NMR (400 MHz, $CDCl_3$) δ 7.66 (d, J = 7.8 Hz, 1H), 7.56 (s, 1H), 7.52 (t, J = 7.7 Hz, 1H), 7.45 (d, J = 7.7 Hz, 1H), 4.01 (q, J = 8.5, 7.5 Hz, 4H), 2.62 (s, 6H), 0.93 (t, J = 7.2 Hz, 6H). ^{13}C NMR (101 MHz, $CDCl_3$) δ 167.41, 155.88, 144.54, 137.42, 131.60, 130.66 (q, J = 32.5 Hz), 128.71, 126.72, 125.17, 122.42, 61.54, 23.00, 13.50. M.p. 61.0-61.3 °C.

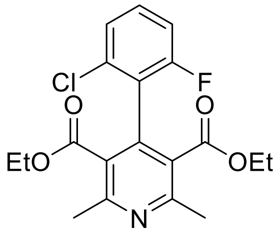

Compound **2r** (reaction time 4 h, white solid) in 89.5% yield**.** ^{1}H NMR (400 MHz, $CDCl_3$) δ 7.31 (td, J = 8.2, 5.8 Hz, 1H), 7.23 (d, J = 8.1 Hz, 1H), 7.08 – 7.00 (m, 1H), 4.06 (q, J = 7.4, 6.9 Hz, 4H), 2.67 (s, 6H), 0.97 (t, J = 7.1 Hz, 6H). ^{13}C NMR (101 MHz, $CDCl_3$) δ 166.67, 159.96 (d, J = 250.6 Hz), 157.15, 138.83, 134.37 (d, J = 4.1 Hz), 130.44 (d, J = 8.9 Hz), 126.62, 124.78 (d, J = 3.4 Hz), 124.55 (d, J = 20.0 Hz), 113.60 (d, J = 22.1 Hz), 61.41, 23.60, 13.57. M.p. 74.9-75.6 °C.

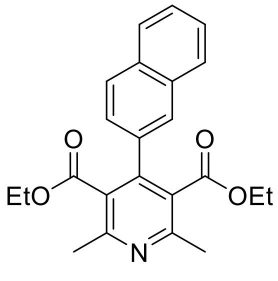

Compound **2s** (reaction time 5 h, yellow solid) in 95.7% yield**.** ^{1}H NMR (400 MHz, $CDCl_3$) δ 7.84 (d, J = 8.4 Hz, 3H), 7.73 (s, 1H), 7.55 – 7.48 (m, 2H), 7.38 (d, J = 8.4 Hz, 1H), 3.94 (q, J = 7.1 Hz, 4H), 2.63 (s, 6H), 0.77 (t, J = 7.1 Hz, 6H). ^{13}C NMR (101 MHz, $CDCl_3$) δ 167.90, 155.48, 146.12, 133.95, 132.88, 132.73, 128.16, 127.80, 127.72, 127.39, 127.18, 126.66, 126.61, 125.89, 61.37, 22.96, 13.53. M.p. 59.5-61.1 °C (lit. [27] 61-62 °C).

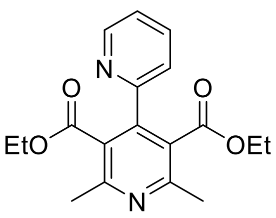

Compound **2t** (reaction time 10 h, white solid) in 85.4% yield. ^{1}H NMR (400 MHz, CDCl$_3$) δ 8.62 (d, *J* = 5.0 Hz, 1H), 7.72 (t, *J* = 7.7 Hz, 1H), 7.36 (d, *J* = 6.9 Hz, 1H), 7.32 – 7.26 (m, 1H), 4.03 (q, *J* = 7.1 Hz, 4H), 2.63 (s, 6H), 0.94 (t, *J* = 7.1 Hz, 6H). ^{13}C NMR (101 MHz, CDCl$_3$) δ 167.77, 156.51, 155.57, 149.37, 145.52, 136.25, 126.13, 123.20, 123.05, 61.37, 23.11, 13.68. M.p. 91.3-93.2 ℃ (lit. [28] 90-92 °C).

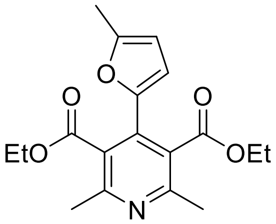

Compound **2u** (reaction time 8.5 h, white solid) in 97% yield. ^{1}H NMR (400 MHz, CDCl$_3$) δ 6.52 (d, *J* = 3.4 Hz, 1H), 6.07 (d, *J* = 2.2 Hz, 1H), 4.29 (q, *J* = 7.2 Hz, 4H), 2.56 (s, 6H), 2.30 (s, 3H), 1.25 (t, *J* = 7.1 Hz, 6H). ^{13}C NMR (101 MHz, CDCl$_3$) δ 168.48, 155.44, 154.18, 146.14, 133.60, 124.18, 113.19, 108.37, 61.69, 22.72, 13.98, 13.61. M.p. 44.7-44.9 ℃.

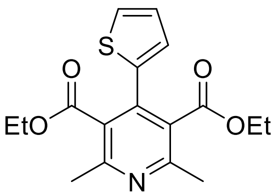

Compound **2v** (reaction time 9 h, white solid) in 96.3% yield. ^{1}H NMR (400 MHz, CDCl$_3$) δ 7.40 (d, *J* = 4.8 Hz, 1H), 7.04 (d, *J* = 4.7 Hz, 2H), 4.12 (q, *J* = 7.1 Hz, 4H), 2.58 (s, 6H), 1.06 (t, *J* = 7.1 Hz, 6H). ^{13}C NMR (101 MHz, CDCl$_3$) δ 167.74, 155.37, 138.85, 135.89, 128.60, 127.63, 127.44, 127.12, 22.88, 13.74. M.p. 77.0-77.3 ℃ (lit. [29] 78-79 °C).

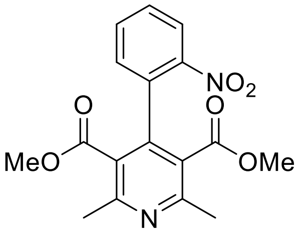

Compound **2w** (reaction time 6 h, white solid) in 97.1% yield. ^{1}H NMR (400 MHz, CDCl$_3$) δ 8.20 (d, *J* = 8.1 Hz, 1H), 7.63 (t, *J* = 7.5 Hz, 1H), 7.60 – 7.54 (m, 1H), 7.20 (d, *J* = 7.5 Hz, 1H), 3.50 (s, 6H), 2.64 (s, 6H). ^{13}C NMR (101 MHz, CDCl$_3$) δ 167.28, 157.10, 147.58, 145.11, 132.97, 131.99, 130.62, 129.60, 124.73, 124.35, 52.22, 23.72. M.p. 95.3-96.7 ℃ (lit. [30] 96 °C).

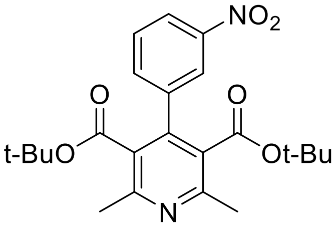

Compound **2x** (reaction time 5 h, white solid) in 95.0% yield. ^{1}H NMR (400 MHz, CDCl$_3$) δ 7.70 (s, 1H), 7.61 (s, 1H), 7.53 (d, *J* = 6.0 Hz, 2H), 2.59(s,6H),1.23(s,18H). ^{13}C NMR (101 MHz, CDCl$_3$) δ 166.38, 154.94, 142.43, 137.90, 133.28, 132.29, 131.83, 128.91, 127.61, 118.07, 112.25, 82.89, 27.64, 22.81. HRMS-ESI: calculated: 429.2026, Observed [M+H]: 429.2032. M.p. 97.4-101.7 ℃.

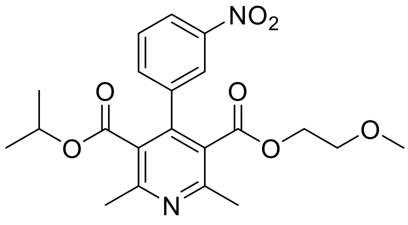

Compound **2y** (reaction time 9 h, colorless oil) in 96.6% yield. ^{1}H NMR (400 MHz, $CDCl_3$) δ 8.26 (d, *J* = 7.3 Hz, 1H), 8.19 (s, 1H), 7.65 – 7.54 (m, 2H), 4.99 – 4.87 (m, 1H), 4.12 (s, 2H), 3.33 (s, 2H), 3.23 (d, *J* = 2.3 Hz, 3H), 2.63 (d, *J* = 5.3 Hz, 6H), 1.01 (s, 6H). ^{13}C NMR (101 MHz, $CDCl_3$) δ 167.20, 166.65, 156.08, 156.01, 147.85, 143.44, 137.96, 134.46, 129.22, 126.92, 126.25, 123.52, 123.35, 69.80, 69.62, 64.36, 58.75, 23.13, 23.01, 21.30.

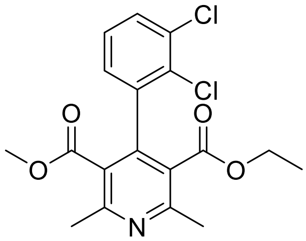

Compound **2z** (reaction time 5 h, colorless oil) in 97.1% yield. ^{1}H NMR (400 MHz, $CDCl_3$) δ 7.48 (d, *J* = 8.2 Hz, 1H), 7.22 (t, *J* = 7.9 Hz, 1H), 7.07 (d, *J* = 7.6 Hz, 1H), 4.02 (q, *J* = 7.2 Hz, 2H), 3.56 (s, 3H), 2.66 (s, 3H), 2.64 (s, 3H), 0.94 (t, *J* = 7.0 Hz, 3H). ^{13}C NMR (101 MHz, $CDCl_3$) δ 167.38, 166.82, 156.98, 156.76, 144.19, 137.77, 133.12, 131.48, 130.38, 128.31, 126.81, 126.22, 126.03, 61.44, 52.27, 23.48, 13.50.

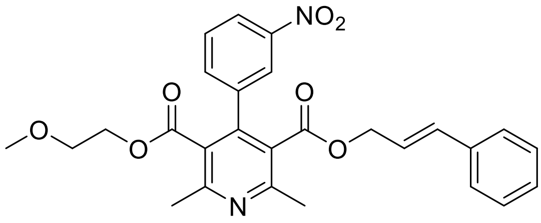

Compound **2aa** (reaction time 5 h, colorless oil) in 94.3% yield. ^{1}H NMR (400 MHz, $CDCl_3$) δ 8.16 (s, 1H), 7.99 (d, *J* = 8.3 Hz, 1H), 7.57 (d, J = 5.1 Hz, 1H), 7.41 (t, *J* = 7.9 Hz, 1H), 7.37 – 7.28 (m, 3H), 7.25 (d, *J* = 8.6 Hz, 2H), 6.45 (d, *J* = 15.8 Hz, 1H), 5.91 – 5.79 (m, 1H), 4.63 (d, *J* = 6.9 Hz, 2H), 4.11 (s, 2H), 3.32 (t, J = 4.6 Hz, 2H), 3.21 (s, 3H), 2.65 (s, 6H). ^{13}C NMR (101 MHz, $CDCl_3$) δ 167.14, 166.98, 156.38, 156.28, 147.77, 143.76, 137.67, 135.92, 135.65, 134.31, 129.13, 128.70, 128.49, 126.59, 126.28, 123.39, 121.09, 69.78, 66.23, 64.37, 58.74, 23.17.

Attachments: spectra of dihydropyridine and products.

Compound **1c**

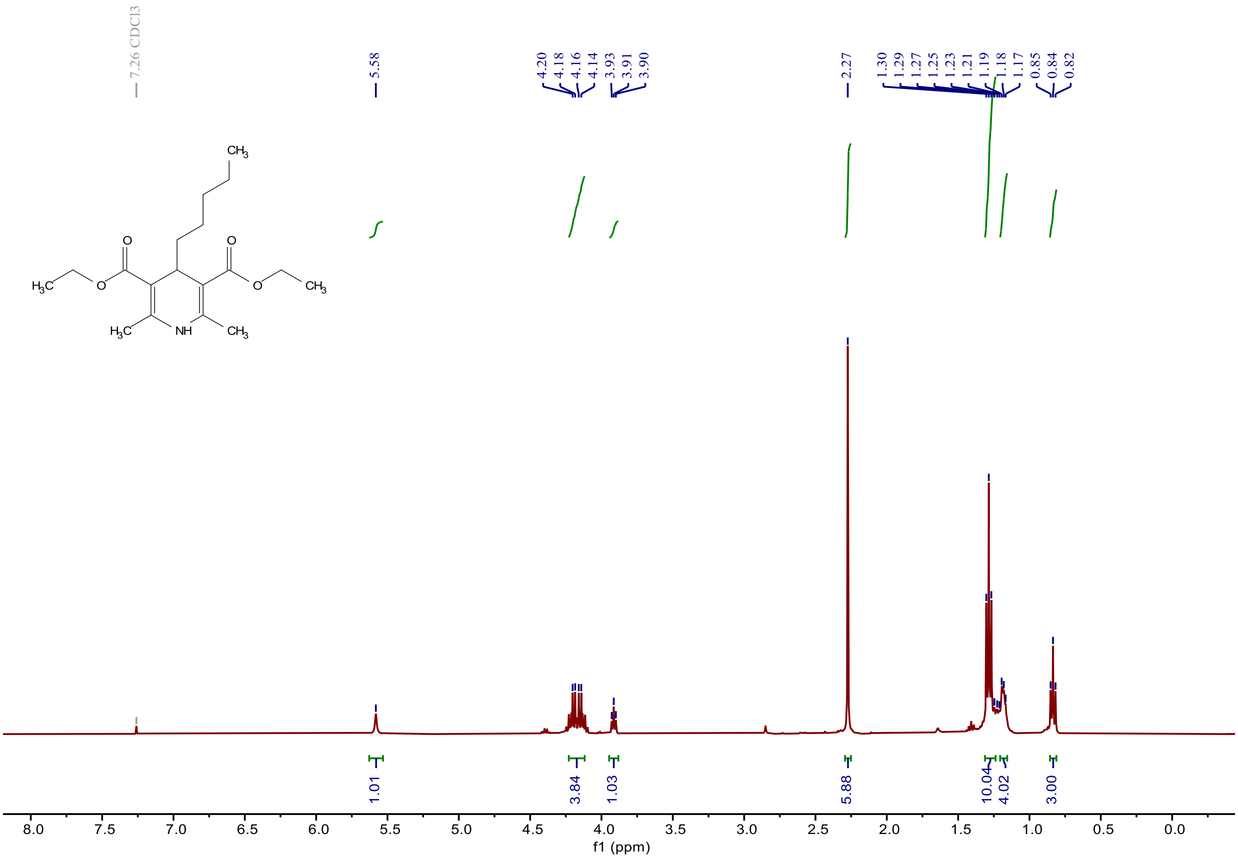

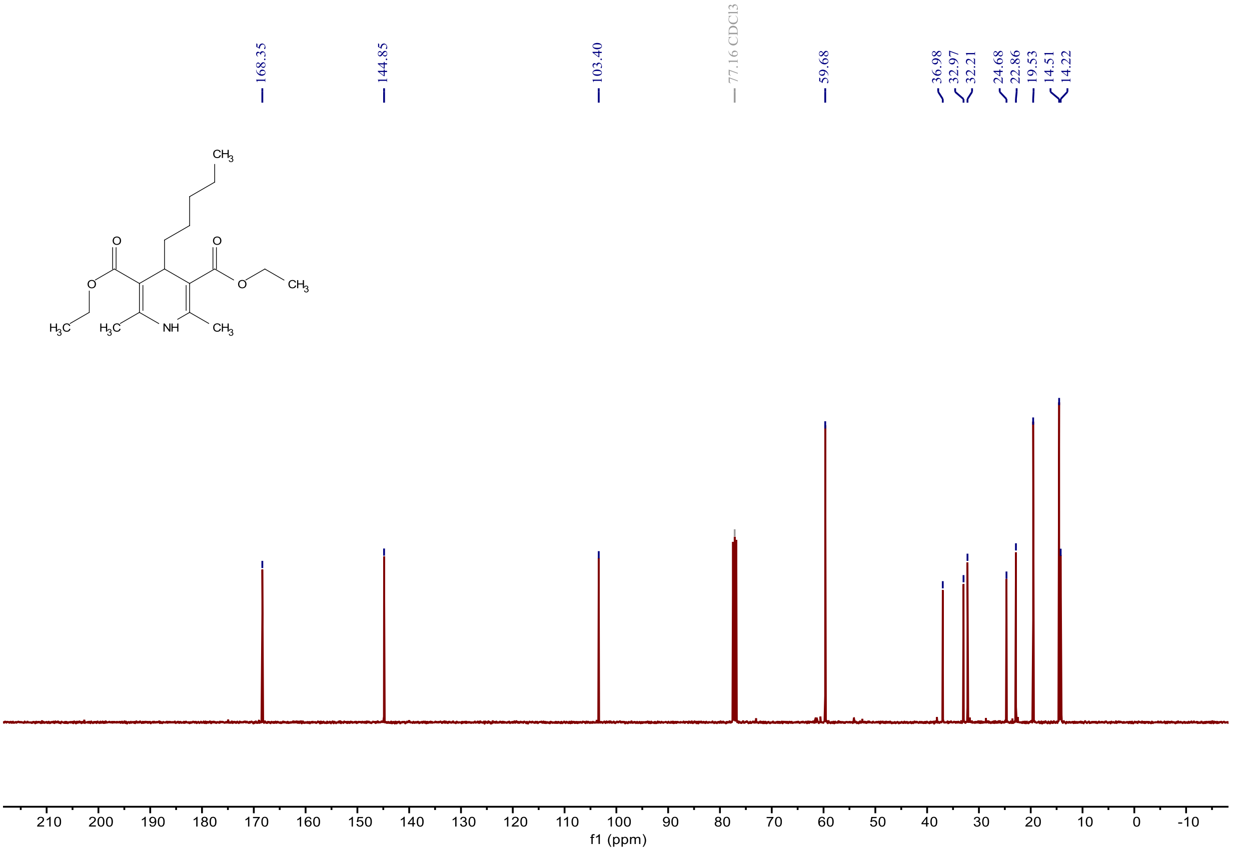

Compound **1d**

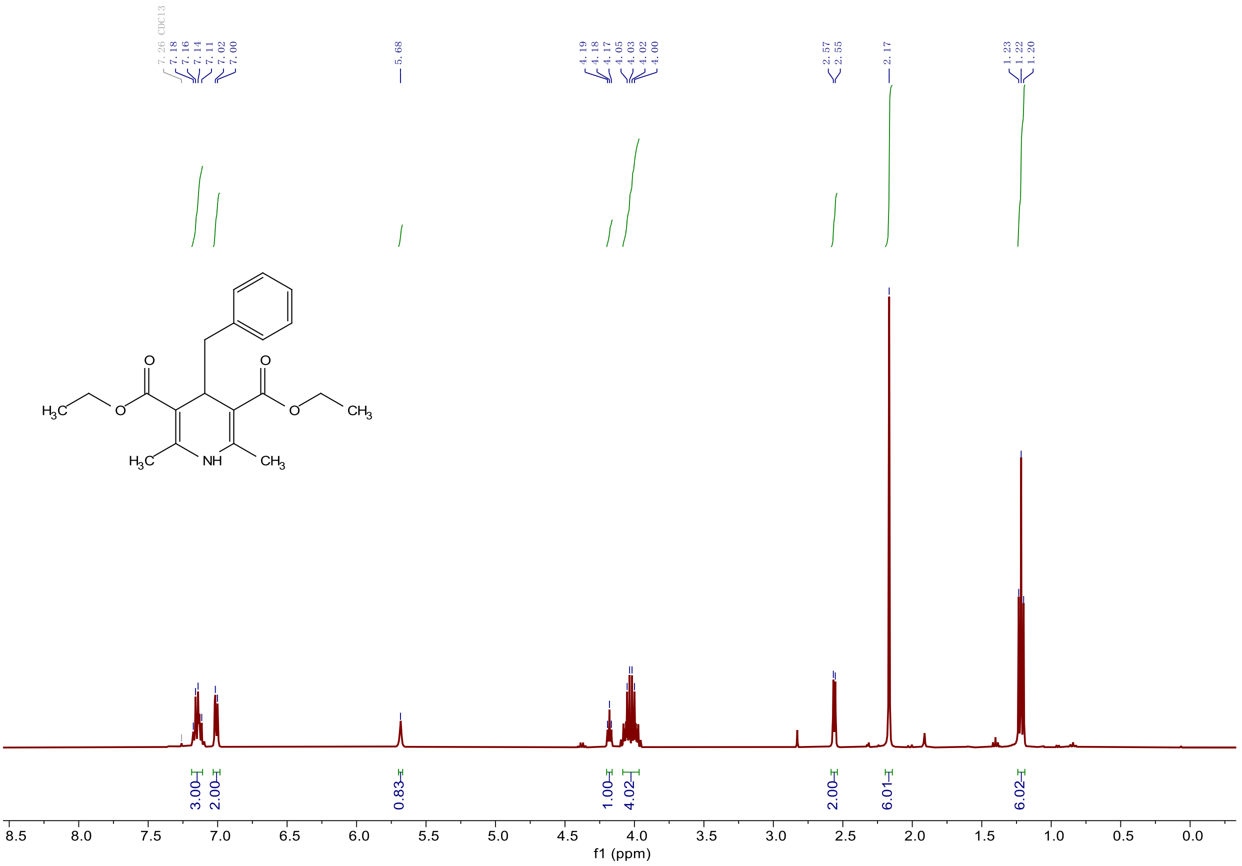
7.26 CDCl3
7.18
7.16
7.14
7.11
7.02
7.00
5.68
4.19
4.18
4.17
4.05
4.03
4.02
4.00
2.57
2.55
2.17
1.23
1.22
1.20
O
O
H3C
O
O
CH3
H3C
NH
CH3
3.00
2.00
0.83
1.00
4.02
2.00
6.01
6.02
f1 (ppm)

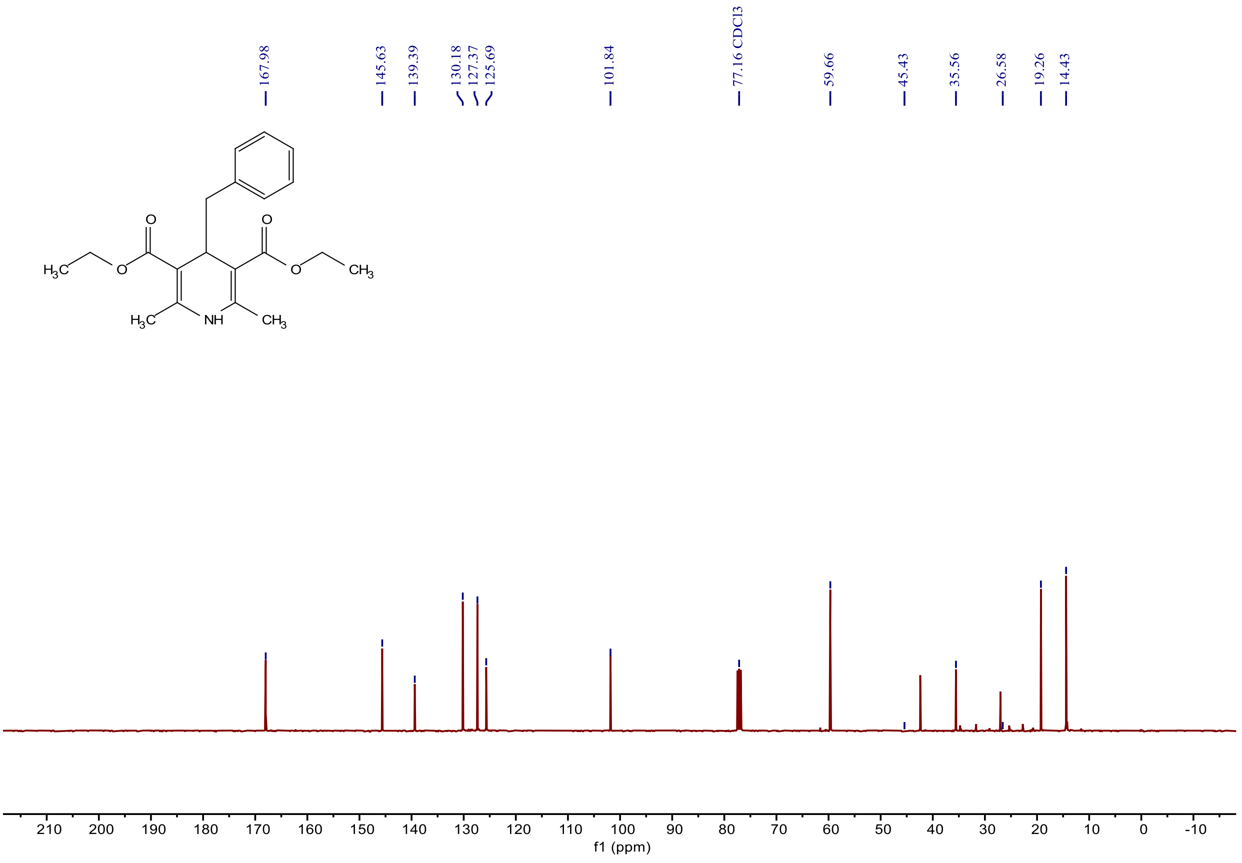
167.98
145.63
139.39
130.18
127.37
125.69
101.84
77.16 CDCl3
59.66
45.43
35.56
26.58
19.26
14.43
O
O
H3C
O
O
CH3
H3C
NH
CH3
f1 (ppm)

Compound **1e**

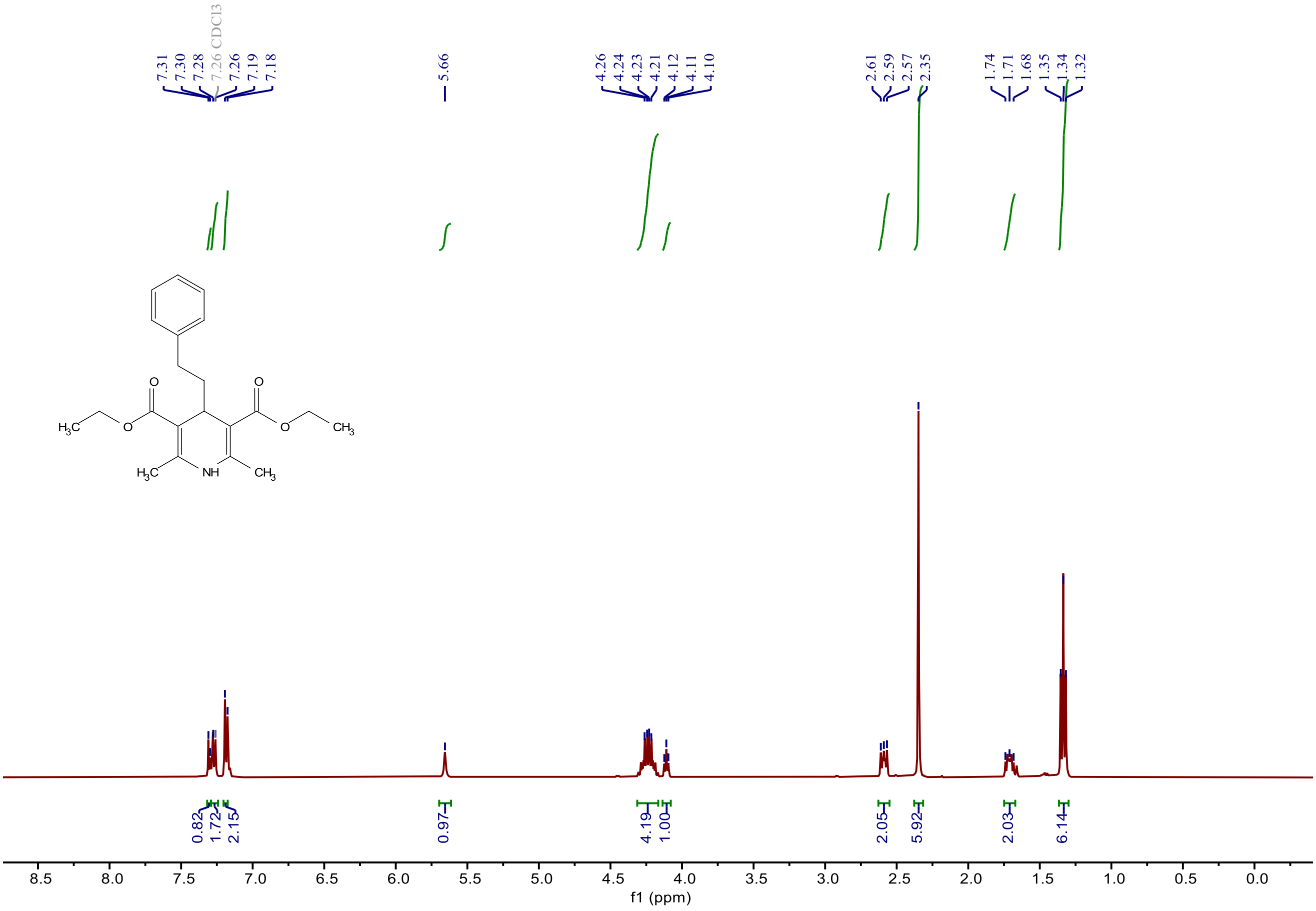
7.31
7.30
7.28
7.26 CDCl3
7.26
7.19
7.18
5.66
4.26
4.24
4.23
4.21
4.12
4.11
4.10
2.61
2.59
2.57
2.35
1.74
1.71
1.68
1.35
1.34
1.32
0.82
1.72
2.15
0.97
4.19
1.00
2.05
5.92
2.03
6.14
f1 (ppm)

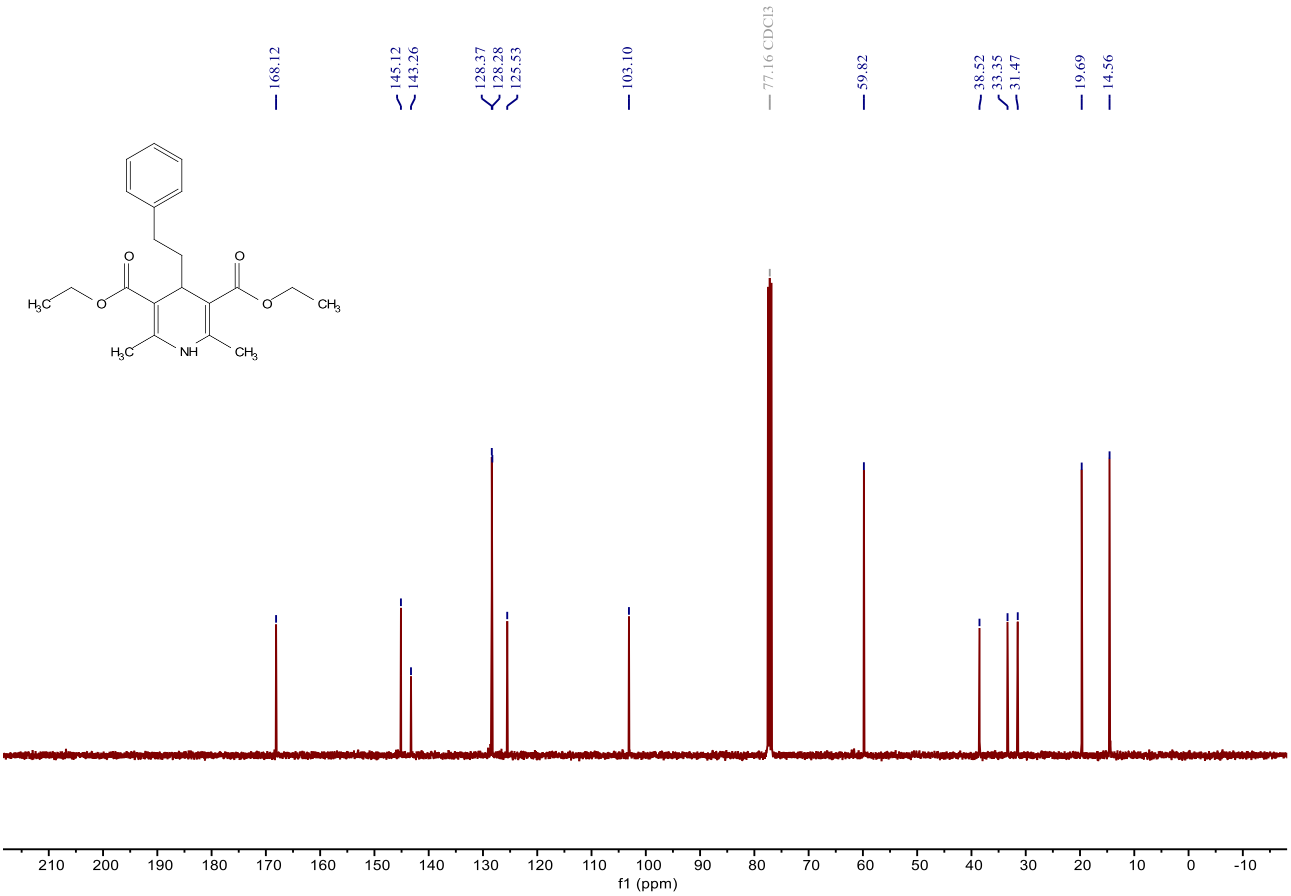
168.12
145.12
143.26
128.37
128.28
125.53
103.10
77.16 CDCl3
59.82
38.52
33.35
31.47
19.69
14.56
f1 (ppm)

Compound **1f**

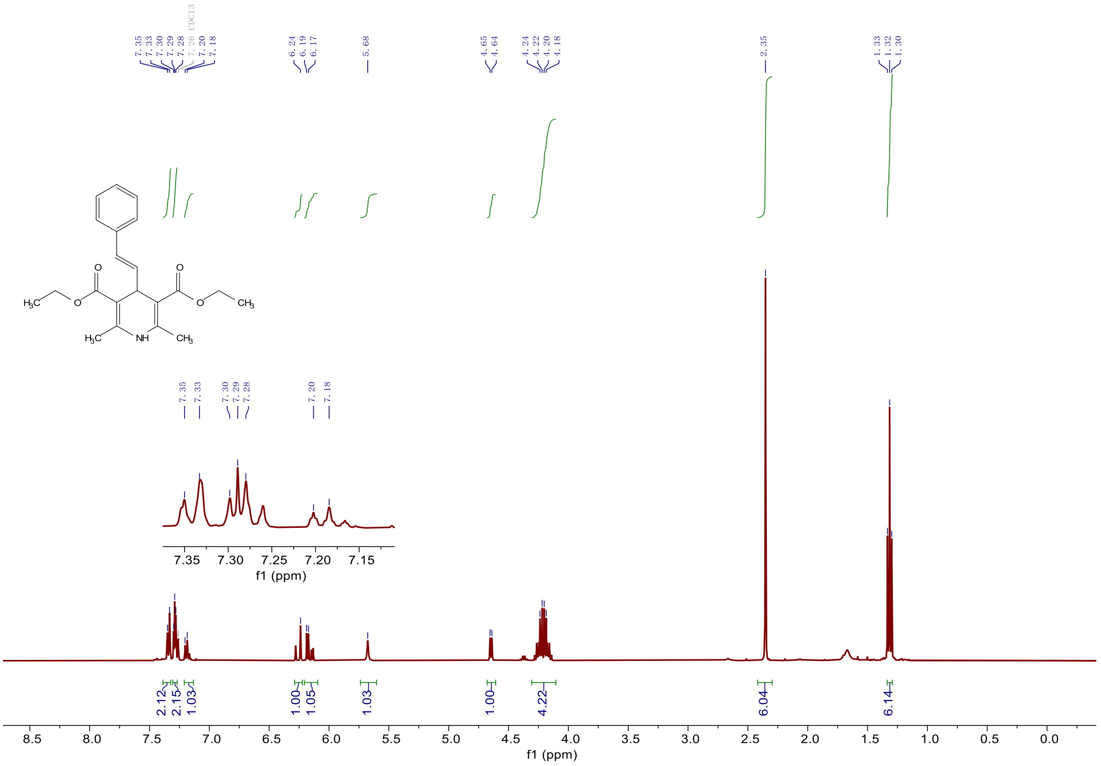

7.35
7.33
7.30
7.29
7.28
7.26 CDCl3
7.20
7.18
6.24
6.19
6.17
5.68
4.65
4.64
4.24
4.22
4.20
4.18
2.35
1.33
1.32
1.30
2.12
2.15
1.03
1.00
1.05
1.03
1.00
4.22
6.04
6.14
f1 (ppm)

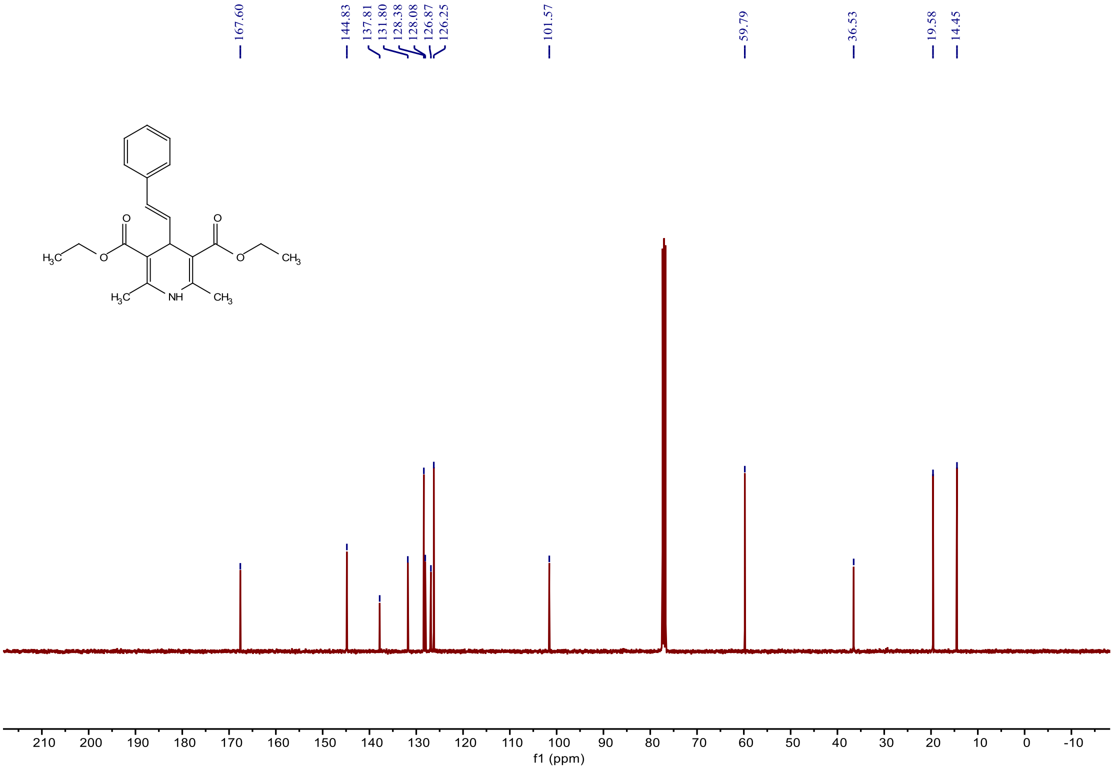

167.60
144.83
137.81
131.80
128.38
128.08
126.87
126.25
101.57
59.79
36.53
19.58
14.45
f1 (ppm)

Compound **1g**

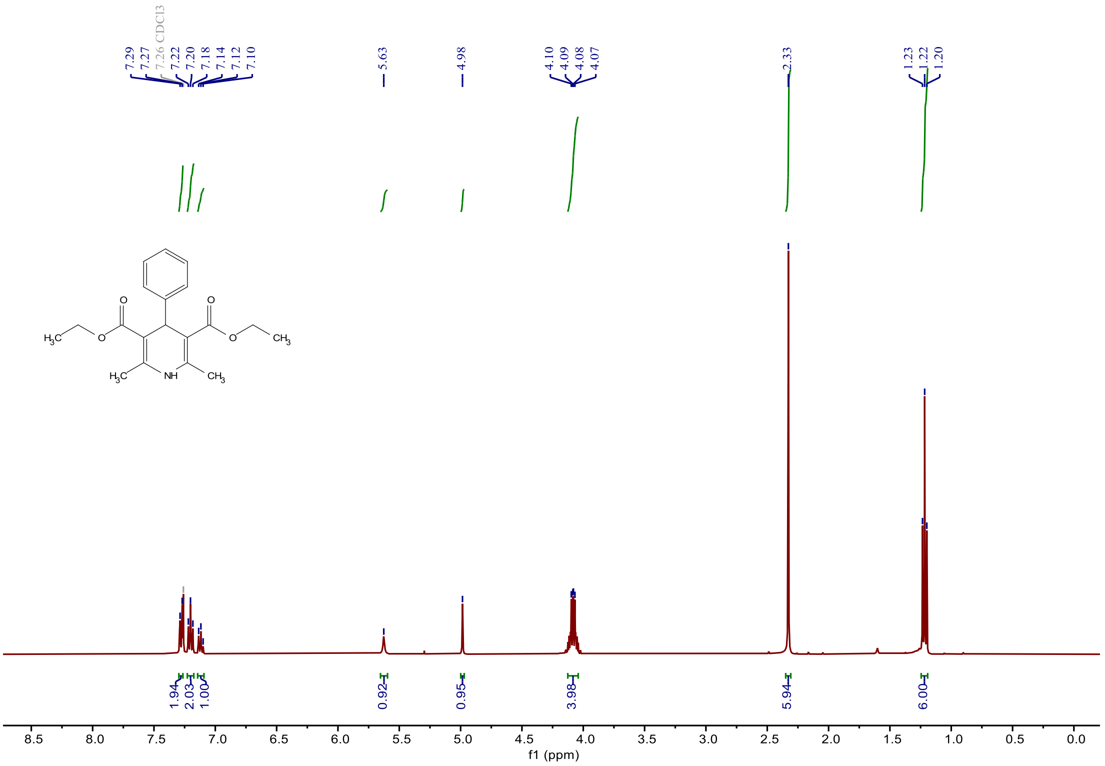

7.29 7.27 7.26 CDCl3 7.22 7.20 7.18 7.14 7.12 7.10
5.63
4.98
4.10 4.09 4.08 4.07
2.33
1.23 1.22 1.20
H3C O O NH CH3
1.94 2.03 1.00
0.92
0.95
3.98
5.94
6.00
f1 (ppm)

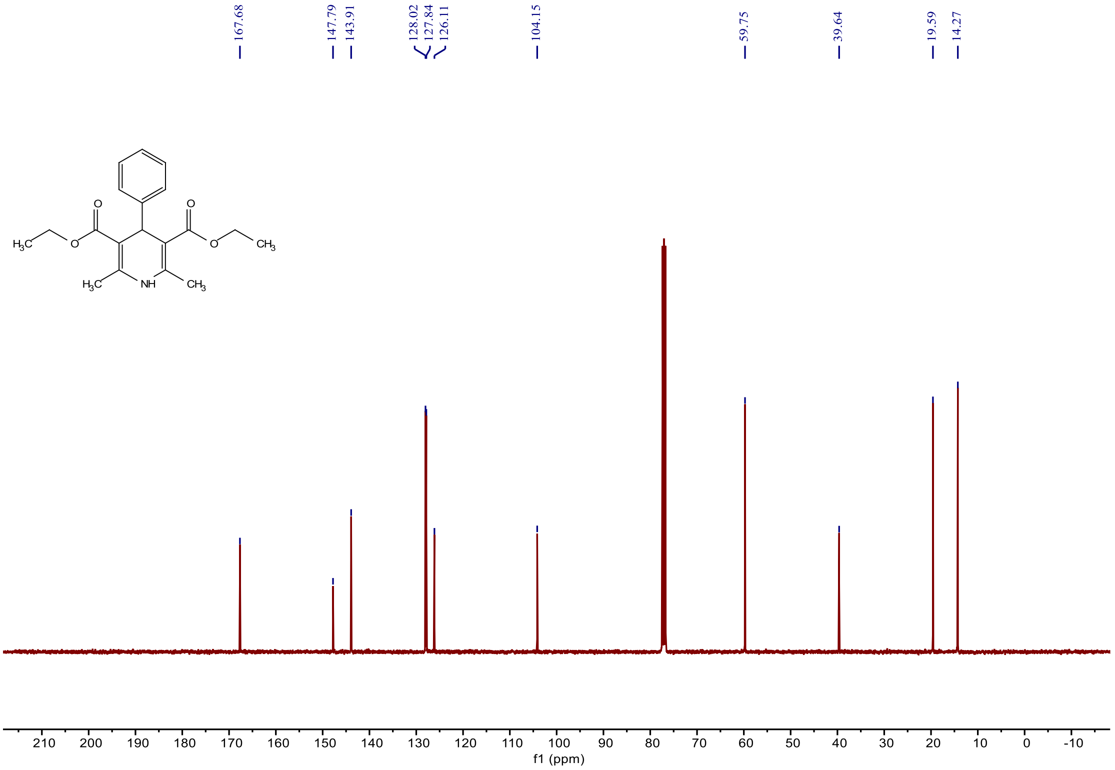

167.68
147.79 143.91
128.02 127.84 126.11
104.15
59.75
39.64
19.59 14.27
H3C O O NH CH3
f1 (ppm)

Compound **1h**

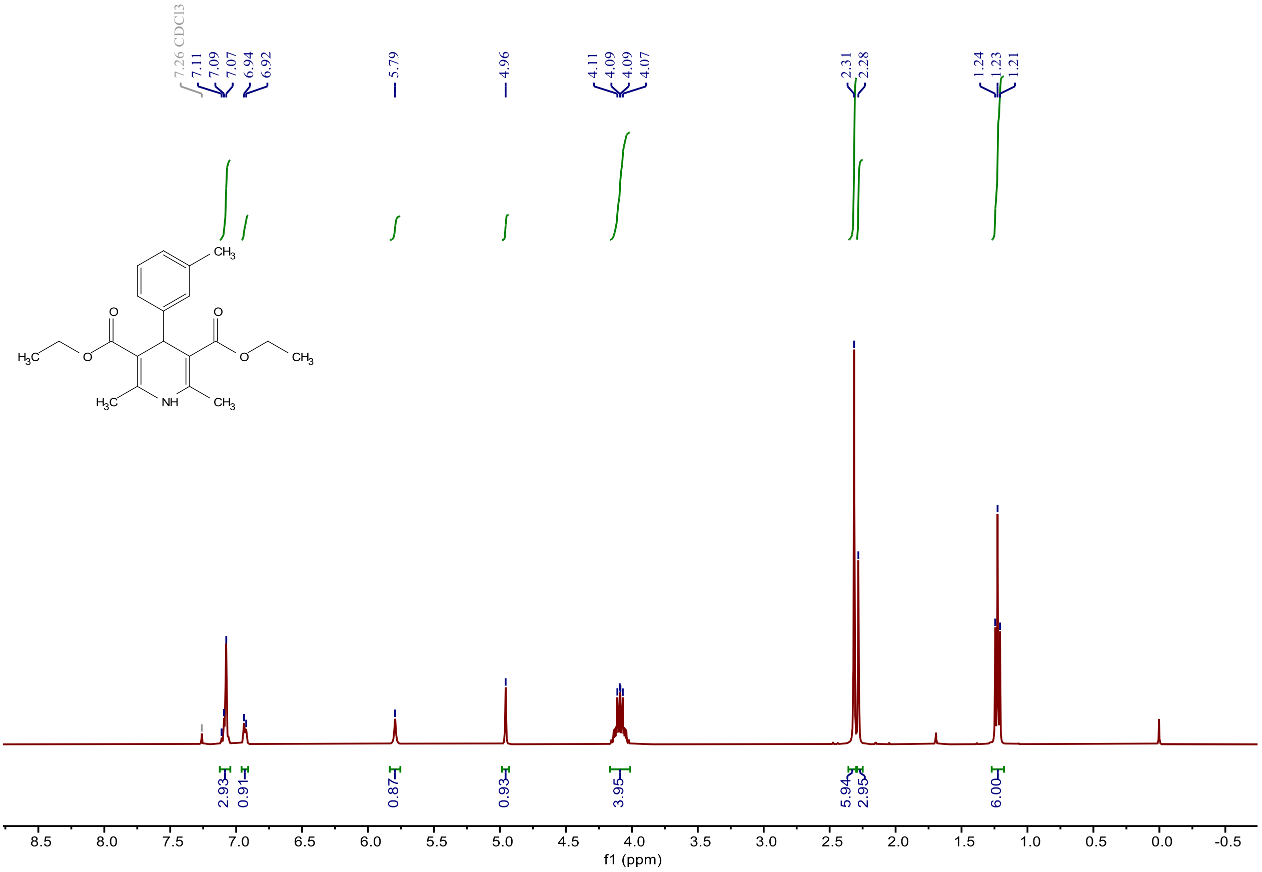
7.26 CDCl3
7.11
7.09
7.07
6.94
6.92
5.79
4.96
4.11
4.09
4.09
4.07
2.31
2.28
1.24
1.23
1.21
2.93
0.91
0.87
0.93
3.95
5.94
2.95
6.00
f1 (ppm)

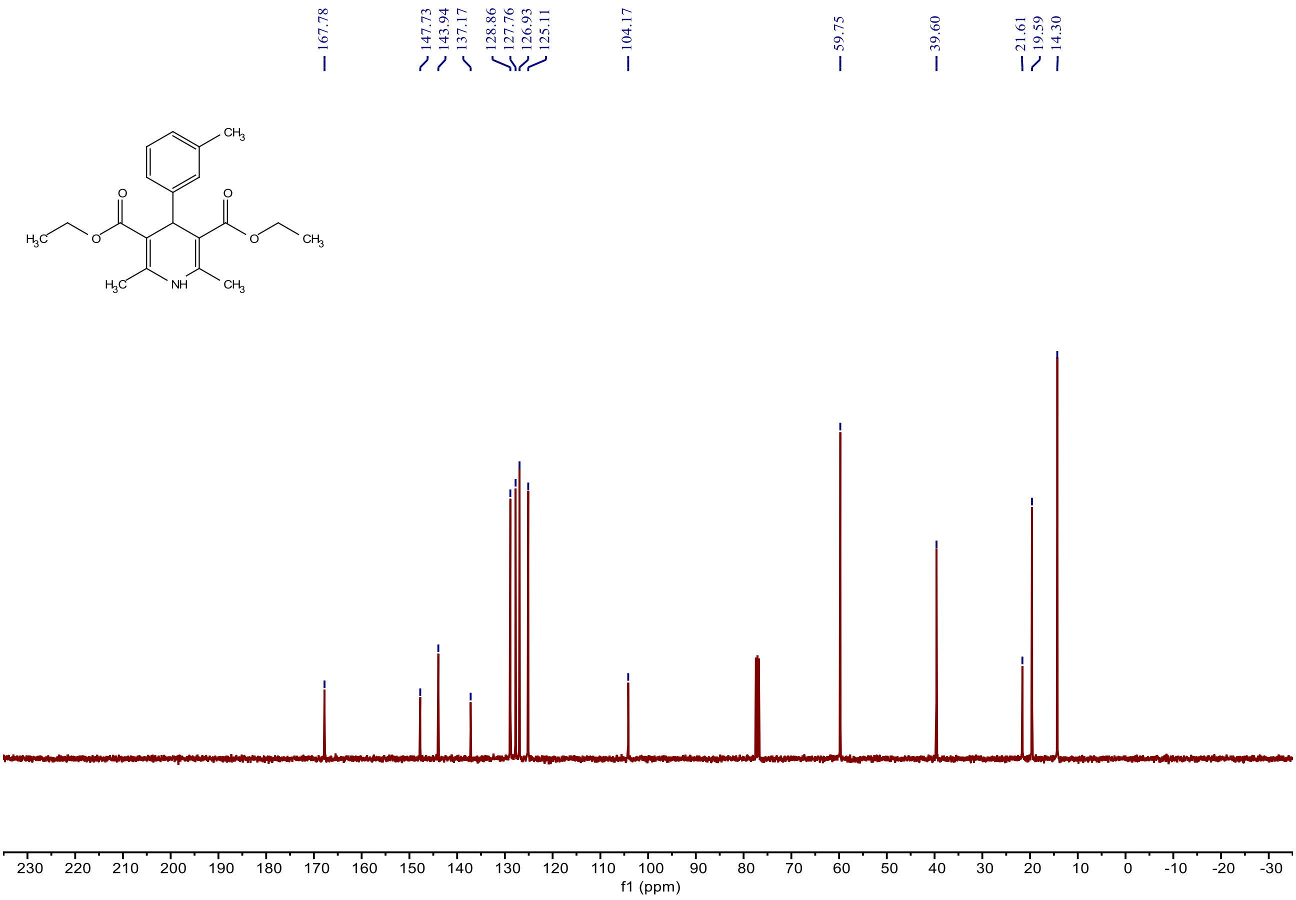
167.78
147.73
143.94
137.17
128.86
127.76
126.93
125.11
104.17
59.75
39.60
21.61
19.59
14.30
f1 (ppm)

Compound **1i**

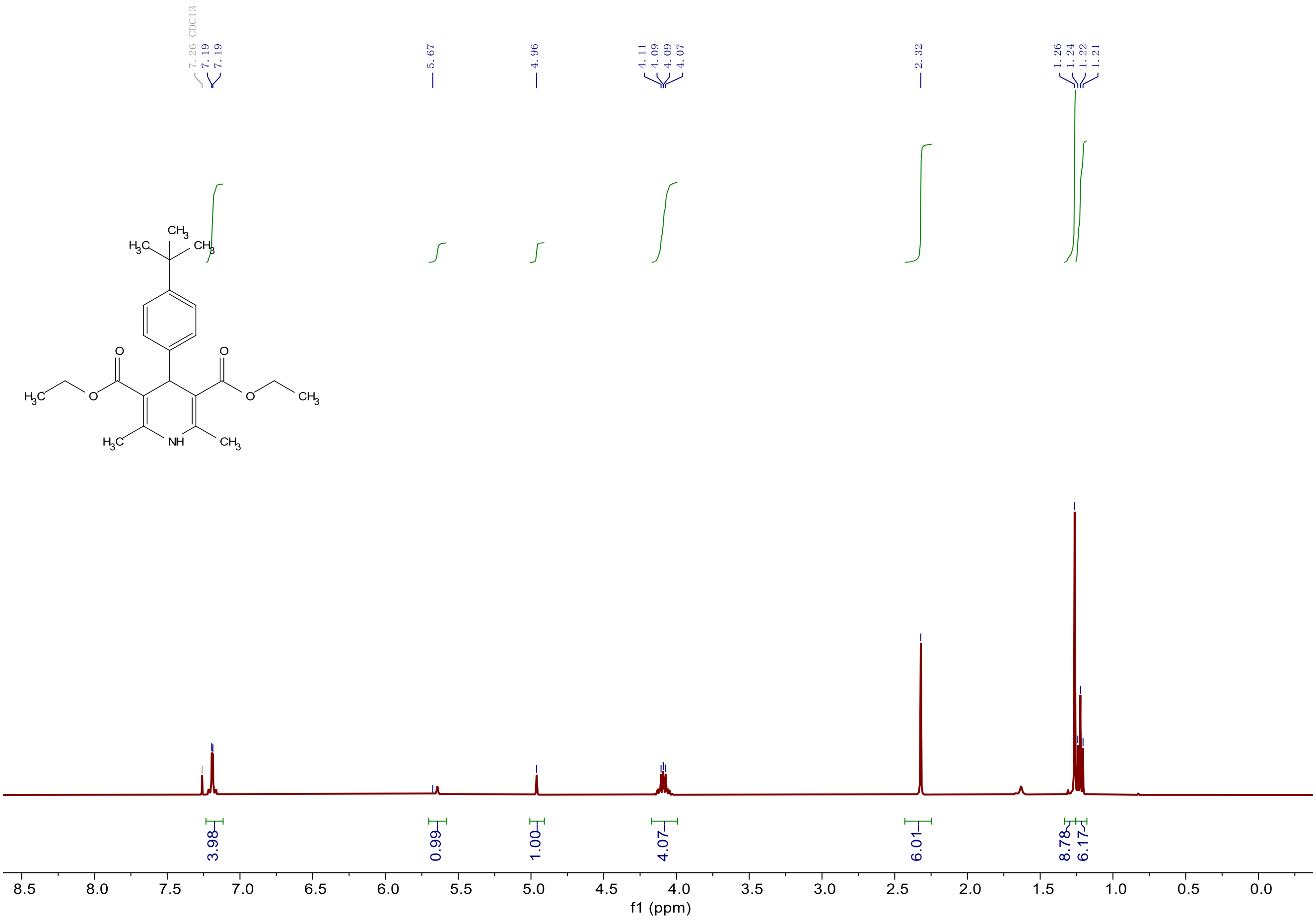

7.26 CDCl3
7.19
7.19
5.67
4.96
4.11
4.09
4.09
4.07
2.32
1.26
1.24
1.22
1.21
CH3
H3C
CH3
O
O
H3C
O
O
CH3
H3C
NH
CH3
3.98
0.99
1.00
4.07
6.01
8.78
6.17
8.5
8.0
7.5
7.0
6.5
6.0
5.5
5.0
4.5
4.0
3.5
3.0
2.5
2.0
1.5
1.0
0.5
0.0
f1 (ppm)

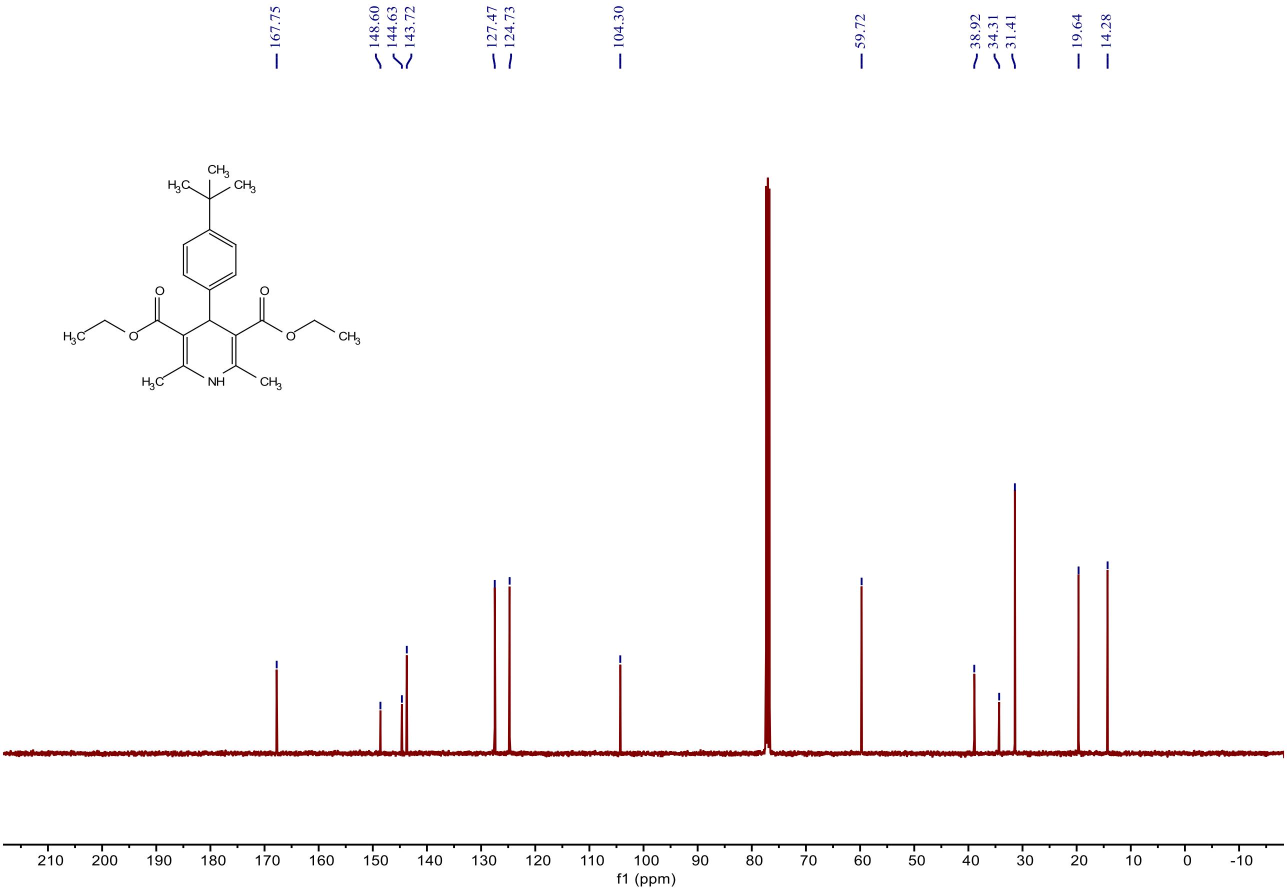

167.75
148.60
144.63
143.72
127.47
124.73
104.30
59.72
38.92
34.31
31.41
19.64
14.28
CH3
H3C
CH3
O
O
H3C
O
O
CH3
H3C
NH
CH3
210
200
190
180
170
160
150
140
130
120
110
100
90
80
70
60
50
40
30
20
10
0
-10
f1 (ppm)

Compound **1j**

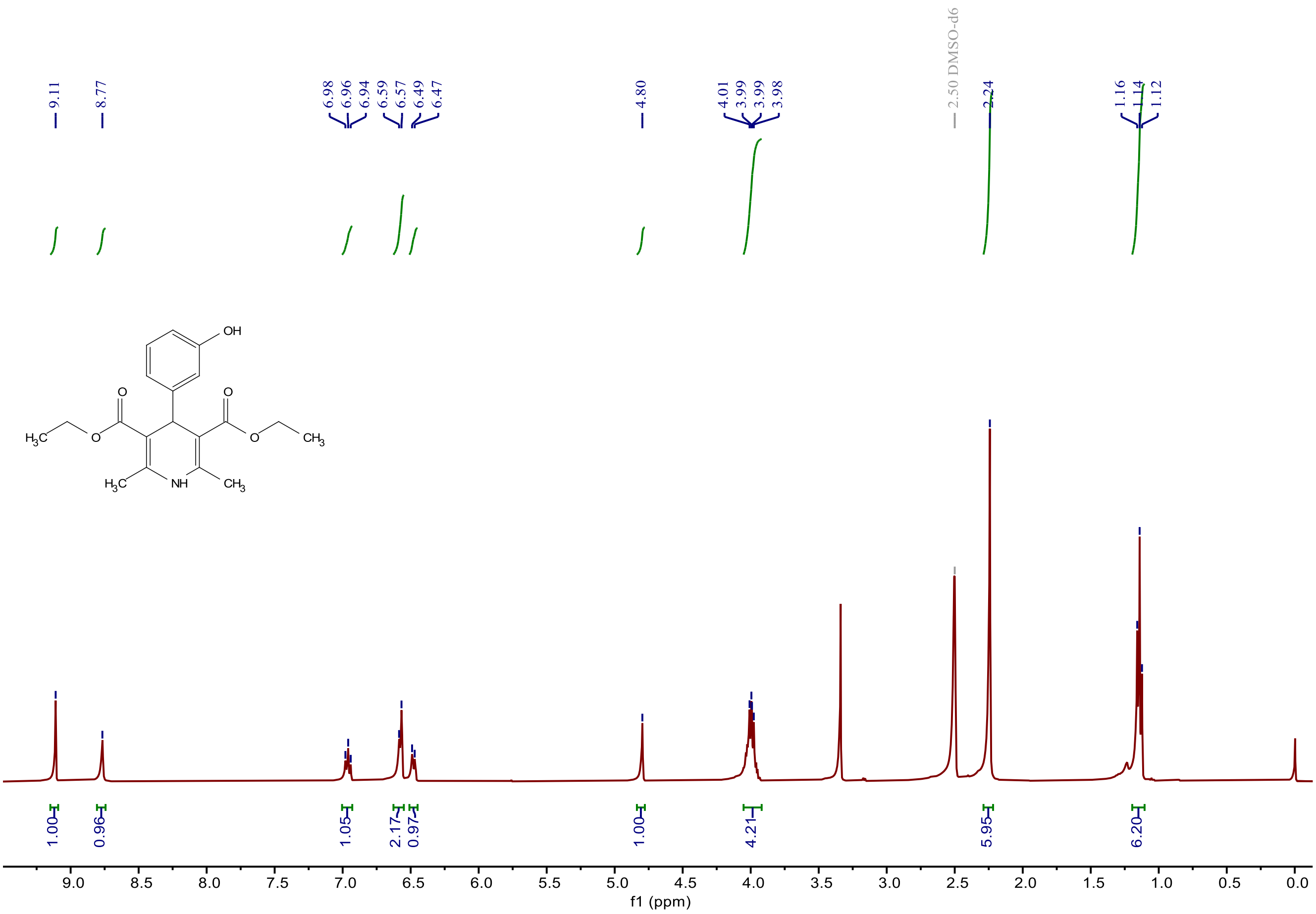
9.11
8.77
6.98
6.96
6.94
6.59
6.57
6.49
6.47
4.80
4.01
3.99
3.99
3.98
2.50 DMSO-d6
2.24
1.16
1.14
1.12
OH
O
O
H_3C
O
O
CH_3
H_3C
NH
CH_3
1.00
0.96
1.05
2.17
0.97
1.00
4.21
5.95
6.20
f1 (ppm)

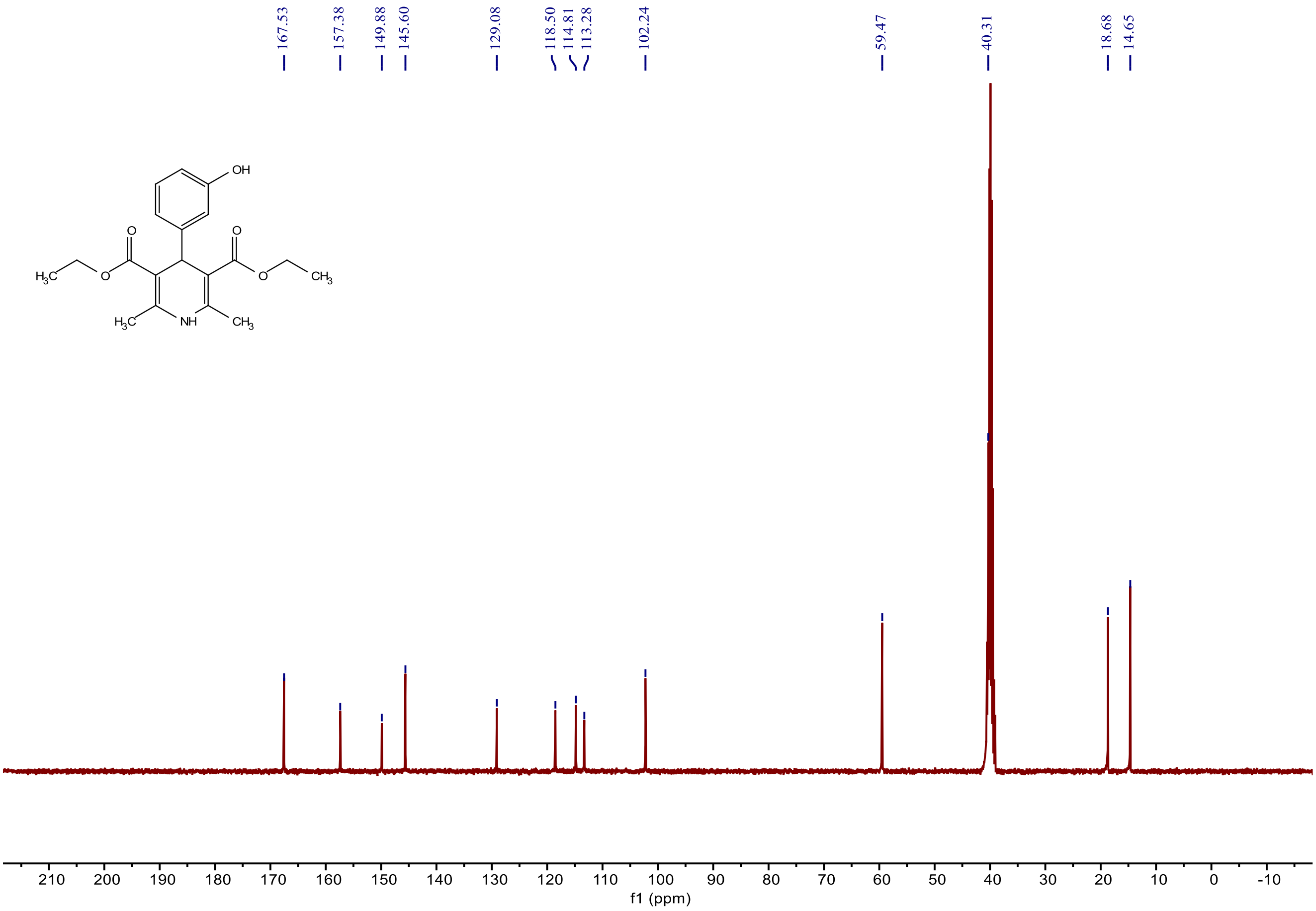
167.53
157.38
149.88
145.60
129.08
118.50
114.81
113.28
102.24
59.47
40.31
18.68
14.65
OH
O
O
H_3C
O
O
CH_3
H_3C
NH
CH_3
f1 (ppm)

Compound **1k**

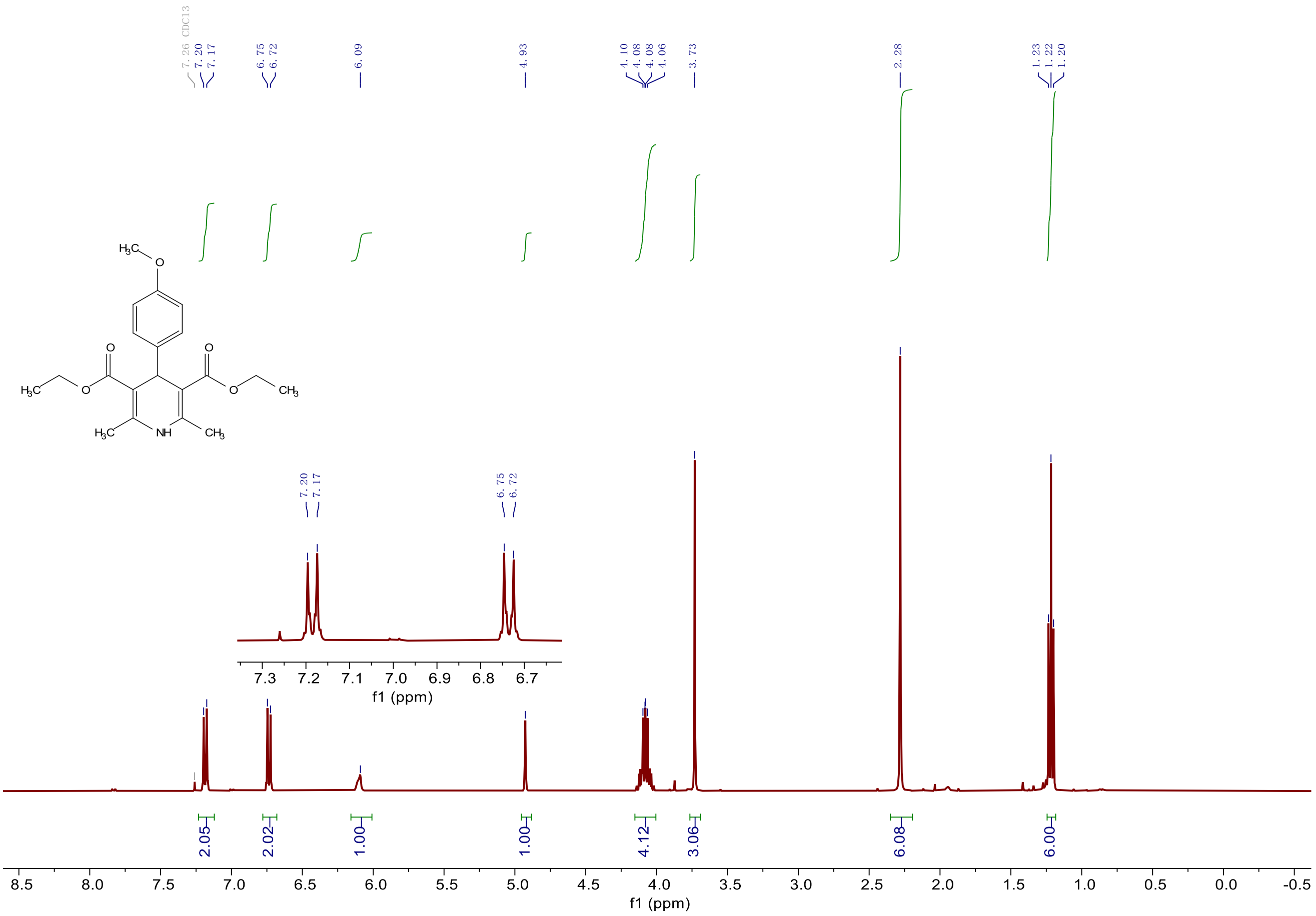

7.26 CDCl3
7.20
7.17
6.75
6.72
6.09
4.93
4.10
4.08
4.08
4.06
3.73
2.28
1.23
1.22
1.20
2.05
2.02
1.00
1.00
4.12
3.06
6.08
6.00
f1 (ppm)

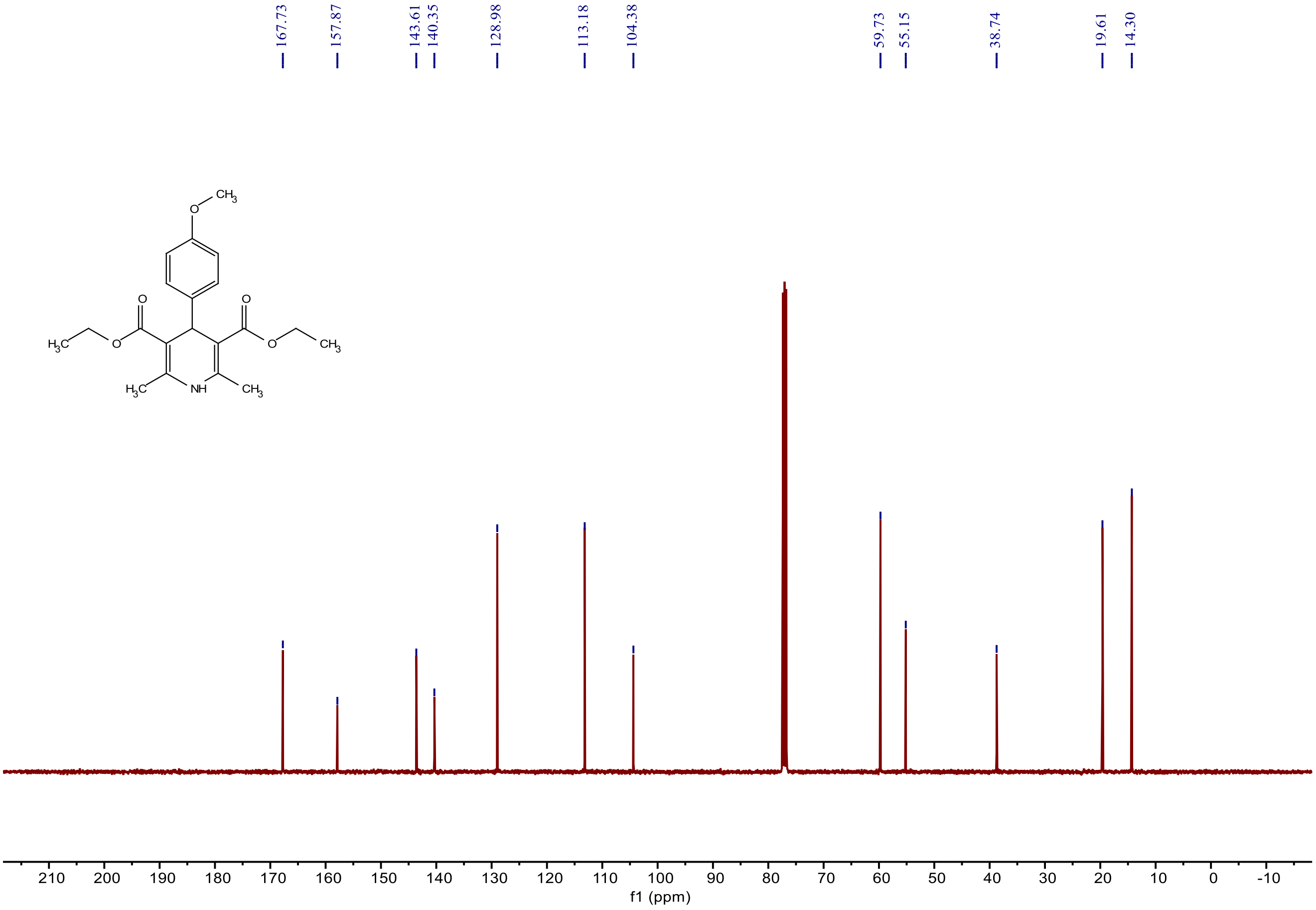

167.73
157.87
143.61
140.35
128.98
113.18
104.38
59.73
55.15
38.74
19.61
14.30
f1 (ppm)

Compound **1l**

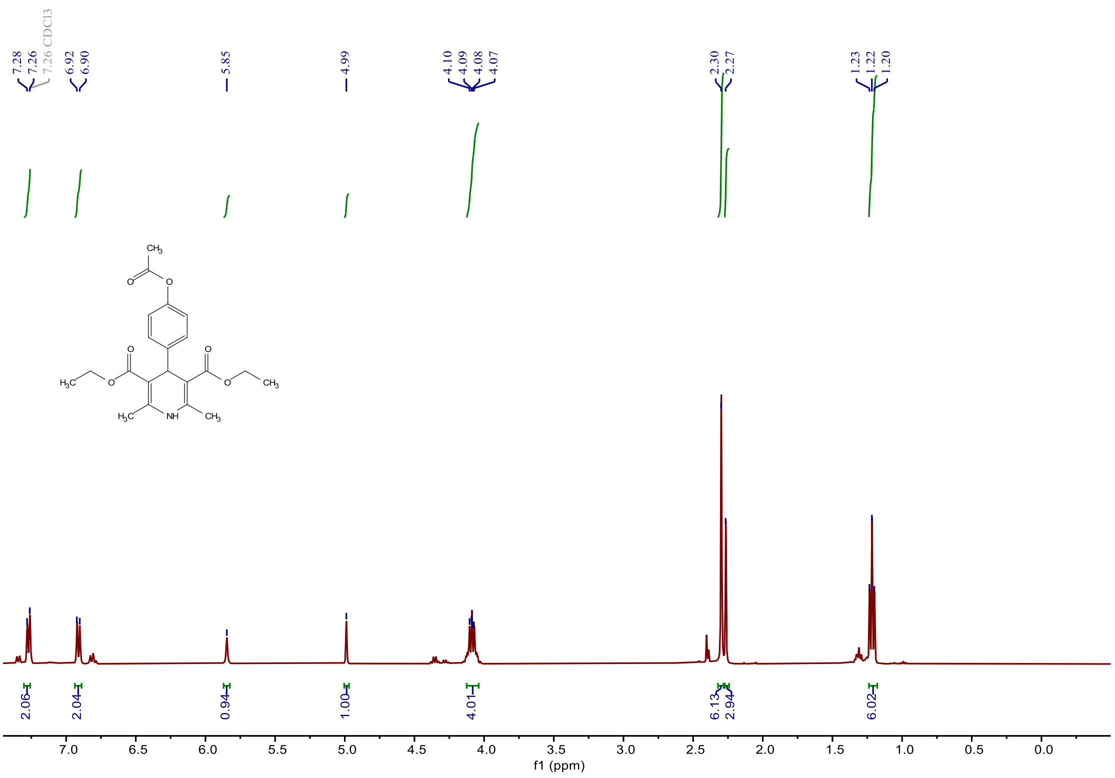
7.28
7.26
7.26 CDCl3
6.92
6.90
5.85
4.99
4.10
4.09
4.08
4.07
2.30
2.27
1.23
1.22
1.20
2.06
2.04
0.94
1.00
4.01
6.13
2.94
6.02
f1 (ppm)

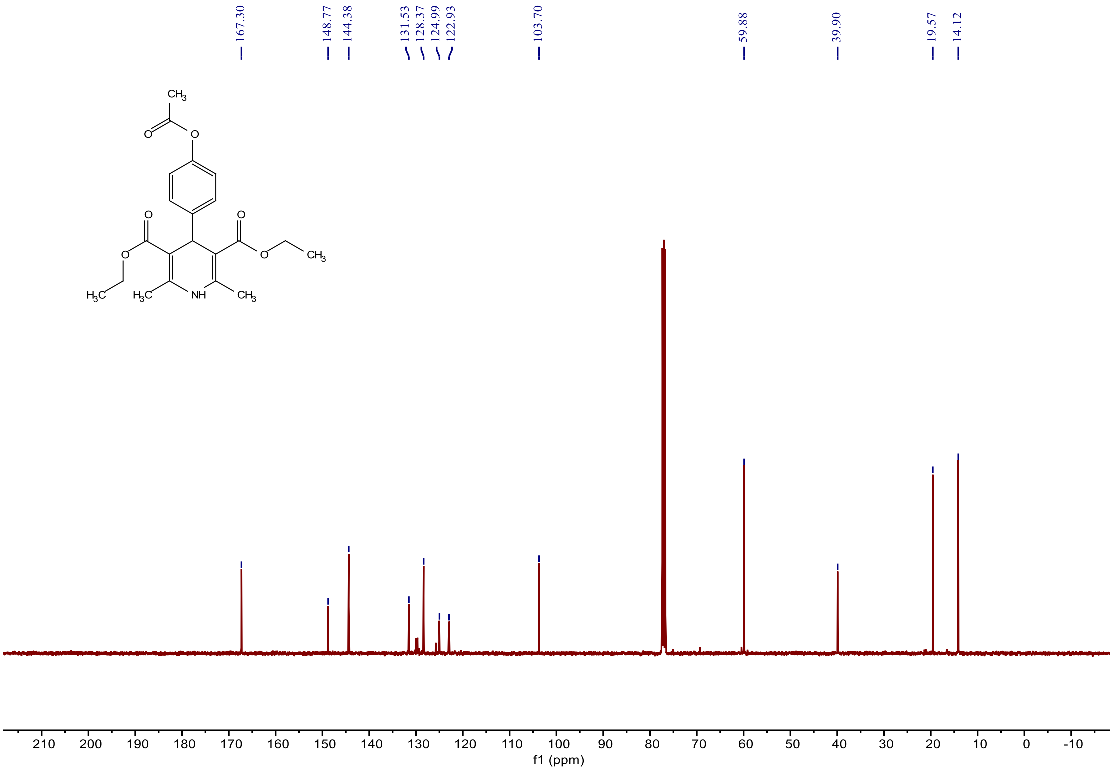
167.30
148.77
144.38
131.53
128.37
124.99
122.93
103.70
59.88
39.90
19.57
14.12
f1 (ppm)

Compound **1m**

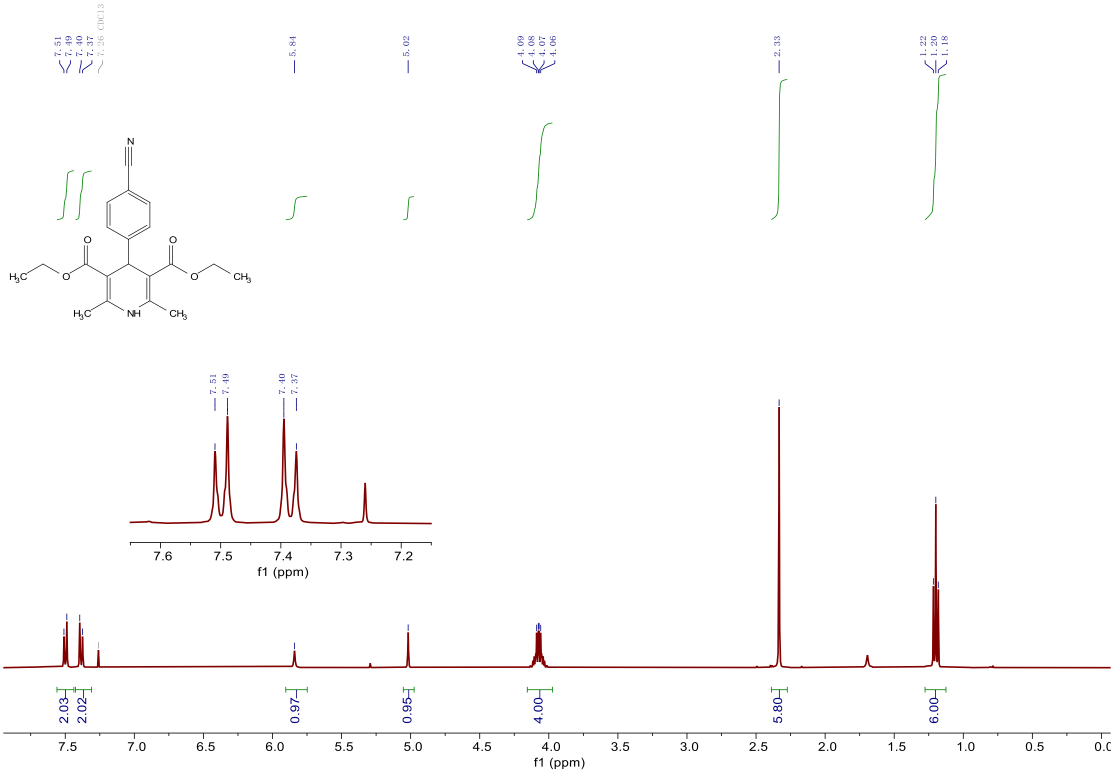
7.51
7.49
7.40
7.37
7.26 CDCl3
5.84
5.02
4.09
4.08
4.07
4.06
2.33
1.22
1.20
1.18
2.03
2.02
0.97
0.95
4.00
5.80
6.00
f1 (ppm)

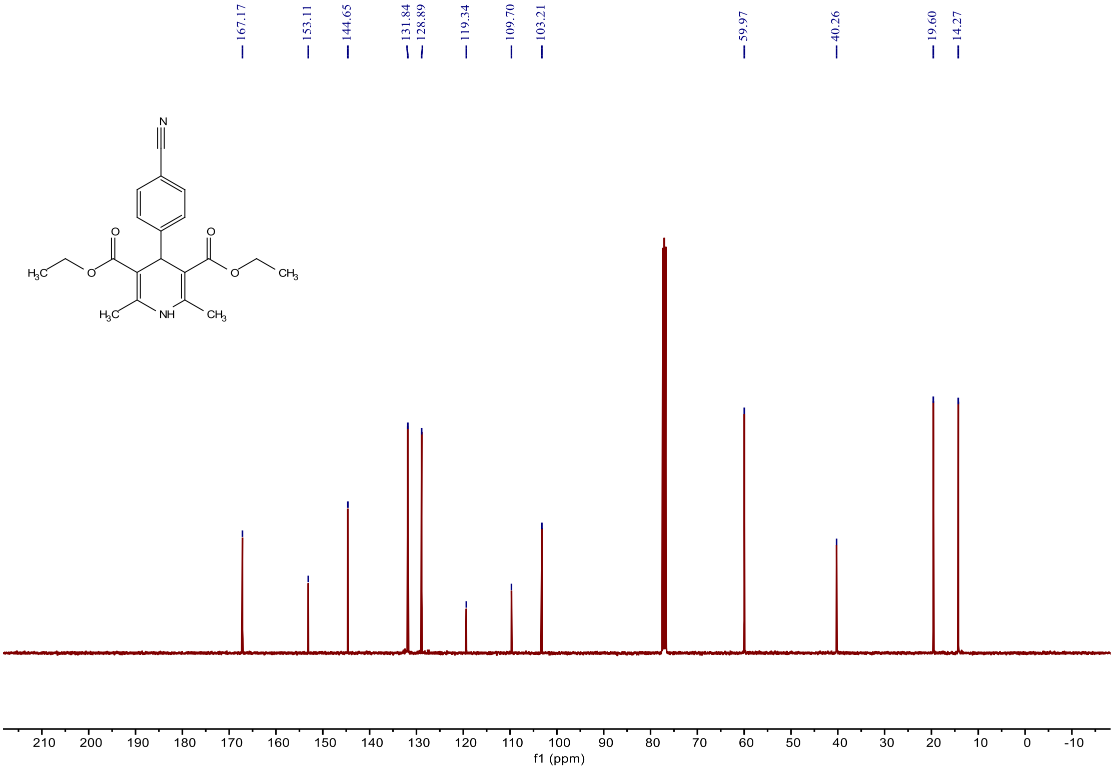
167.17
153.11
144.65
131.84
128.89
119.34
109.70
103.21
59.97
40.26
19.60
14.27
f1 (ppm)

Compound **1n**

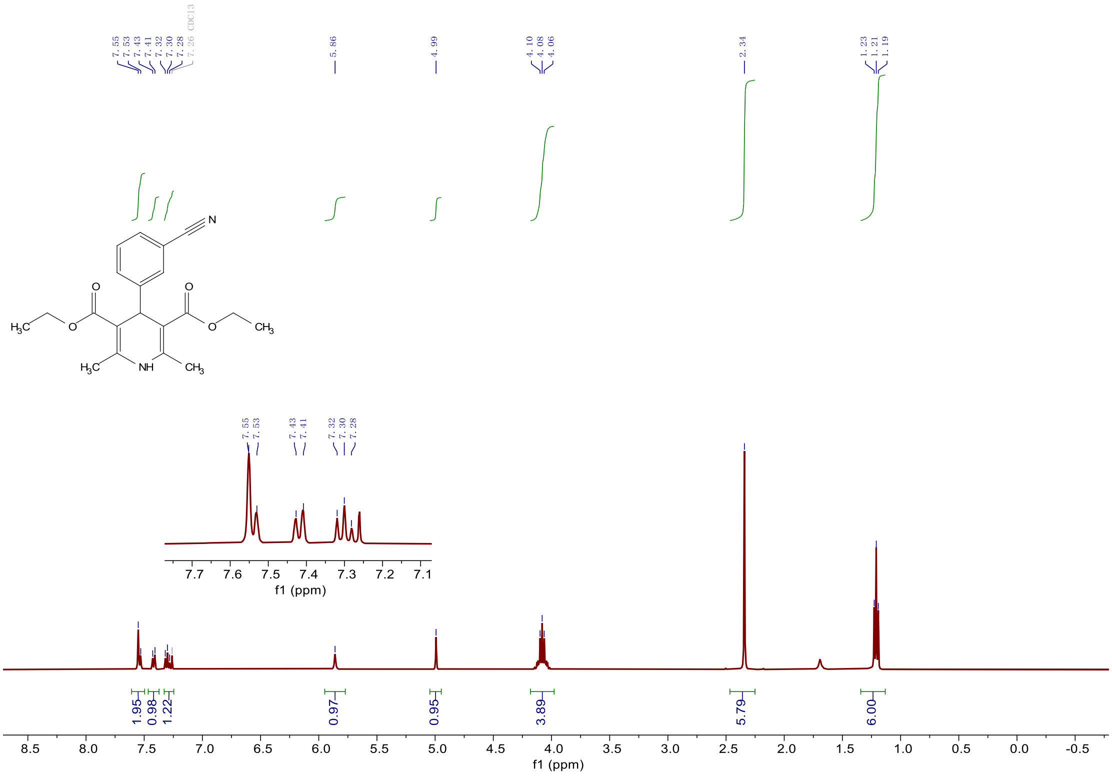

7.55
7.53
7.43
7.41
7.32
7.30
7.28
7.26 CDCl3
5.86
4.99
4.10
4.08
4.06
2.34
1.23
1.21
1.19
N
O
O
H3C
O
O
CH3
H3C
NH
CH3
1.95
0.98
1.22
0.97
0.95
3.89
5.79
6.00
f1 (ppm)

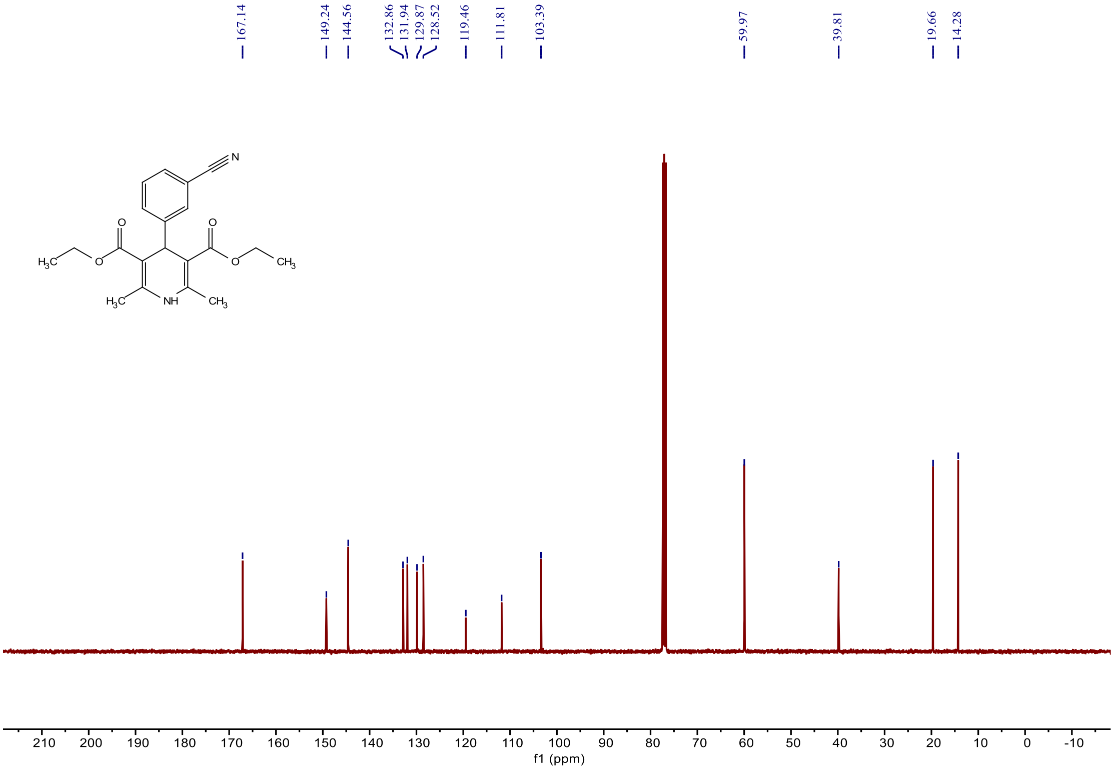

167.14
149.24
144.56
132.86
131.94
129.87
128.52
119.46
111.81
103.39
59.97
39.81
19.66
14.28
N
O
O
H3C
O
O
CH3
H3C
NH
CH3
f1 (ppm)

Compound **1o**

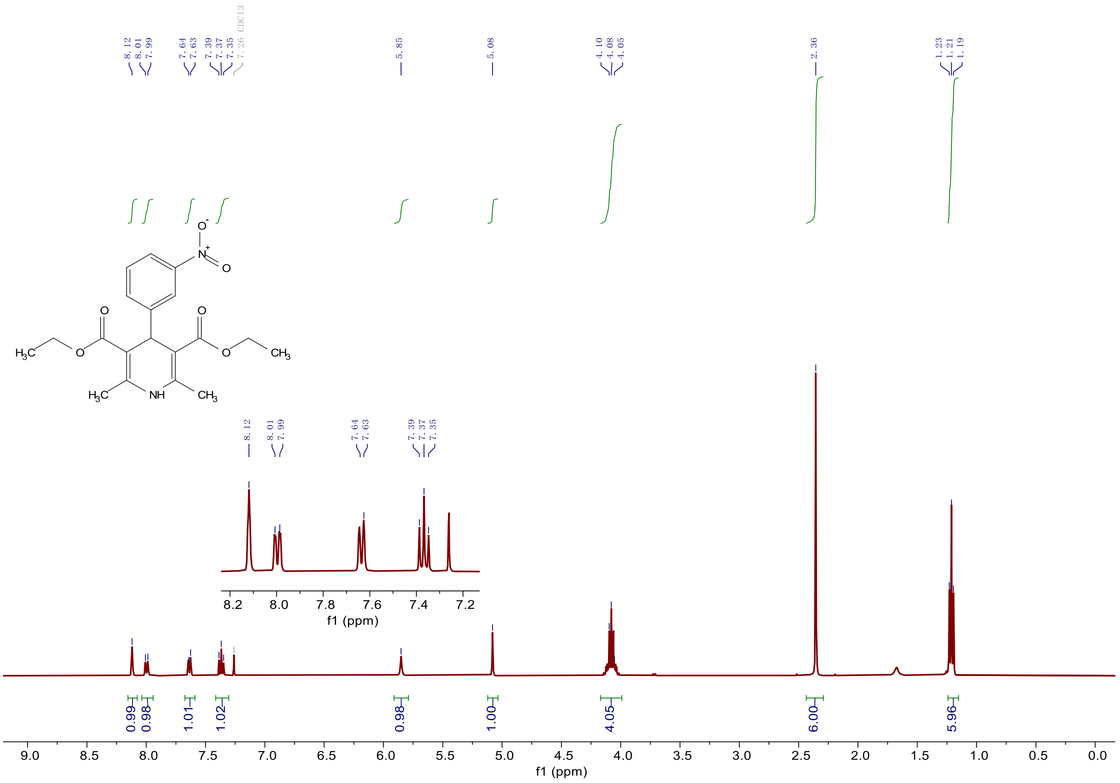
8.12 8.01 7.99 7.64 7.63 7.39 7.37 7.35 7.26 CDCl3
5.85
5.08
4.10 4.08 4.05
2.36
1.23 1.21 1.19
0.99 0.98 1.01 1.02 0.98 1.00 4.05 6.00 5.96
NH
f1 (ppm)

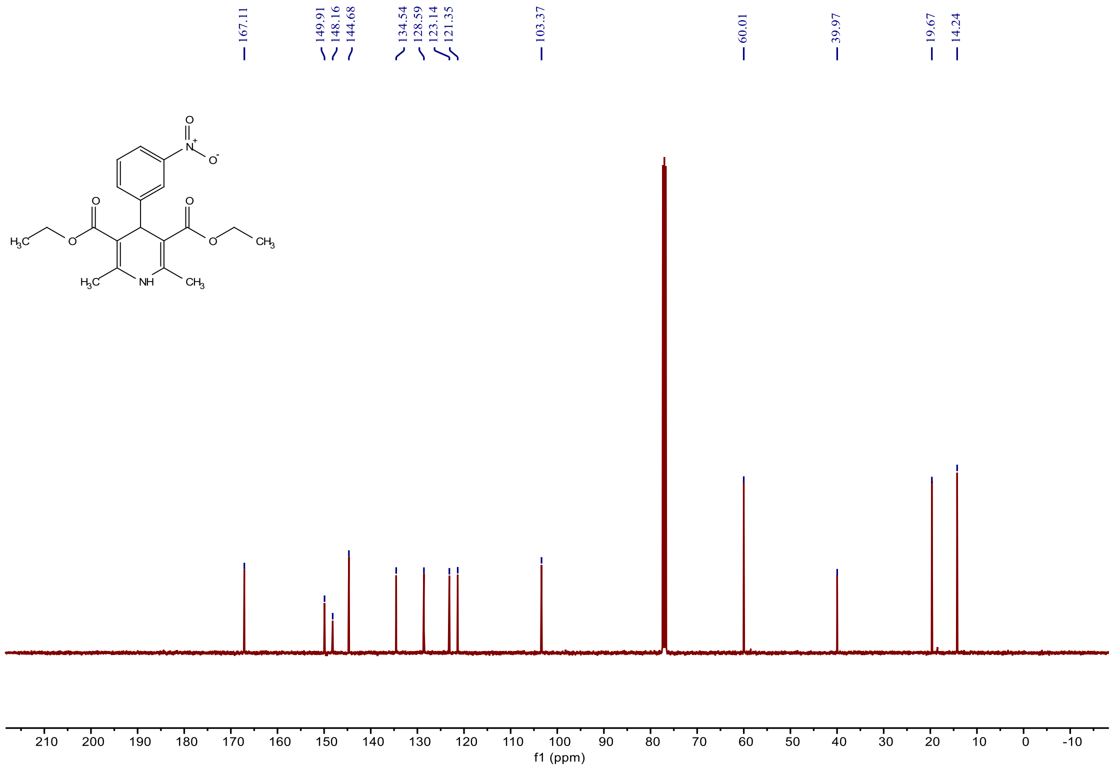
167.11
149.91 148.16 144.68
134.54 128.59 123.14 121.35
103.37
60.01
39.97
19.67
14.24
NH
f1 (ppm)

Compound **1p**

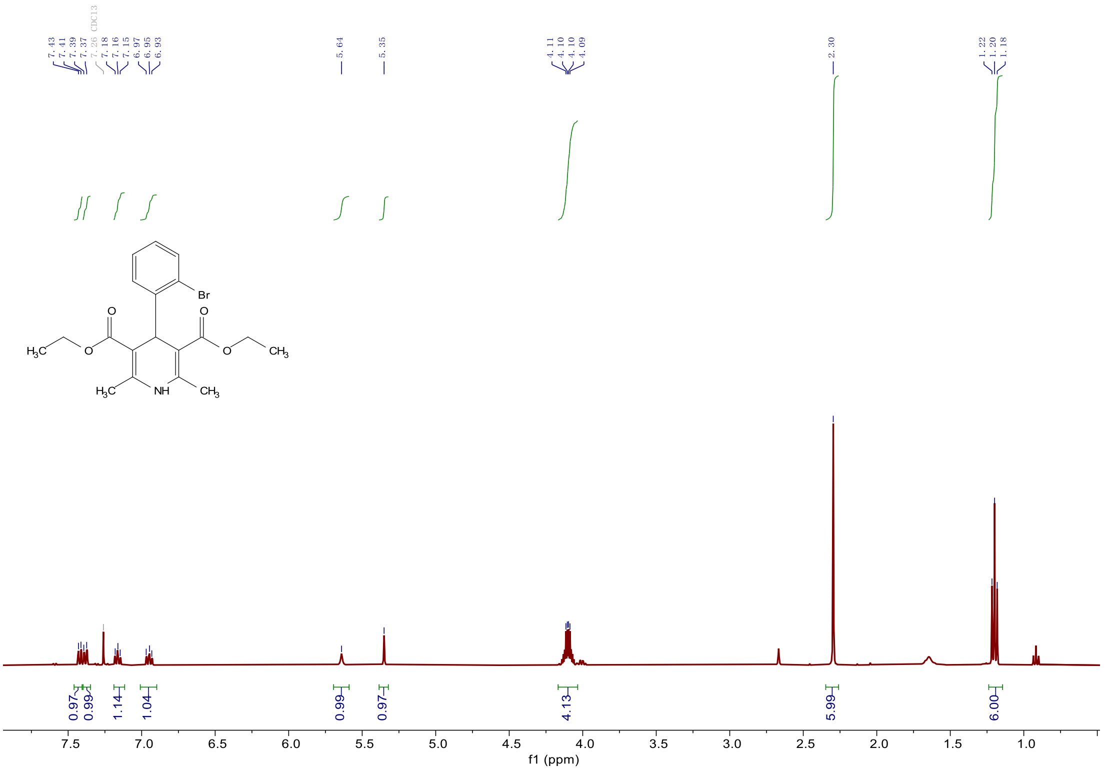

7.43
7.41
7.39
7.37
7.26 CDCl3
7.18
7.16
7.15
6.97
6.95
6.93
5.64
5.35
4.11
4.10
4.10
4.09
2.30
1.22
1.20
1.18
Br
O
O
H3C
O
O
CH3
H3C
NH
CH3
0.97
0.99
1.14
1.04
0.99
0.97
4.13
5.99
6.00
7.5
7.0
6.5
6.0
5.5
5.0
4.5
4.0
3.5
3.0
2.5
2.0
1.5
1.0
f1 (ppm)

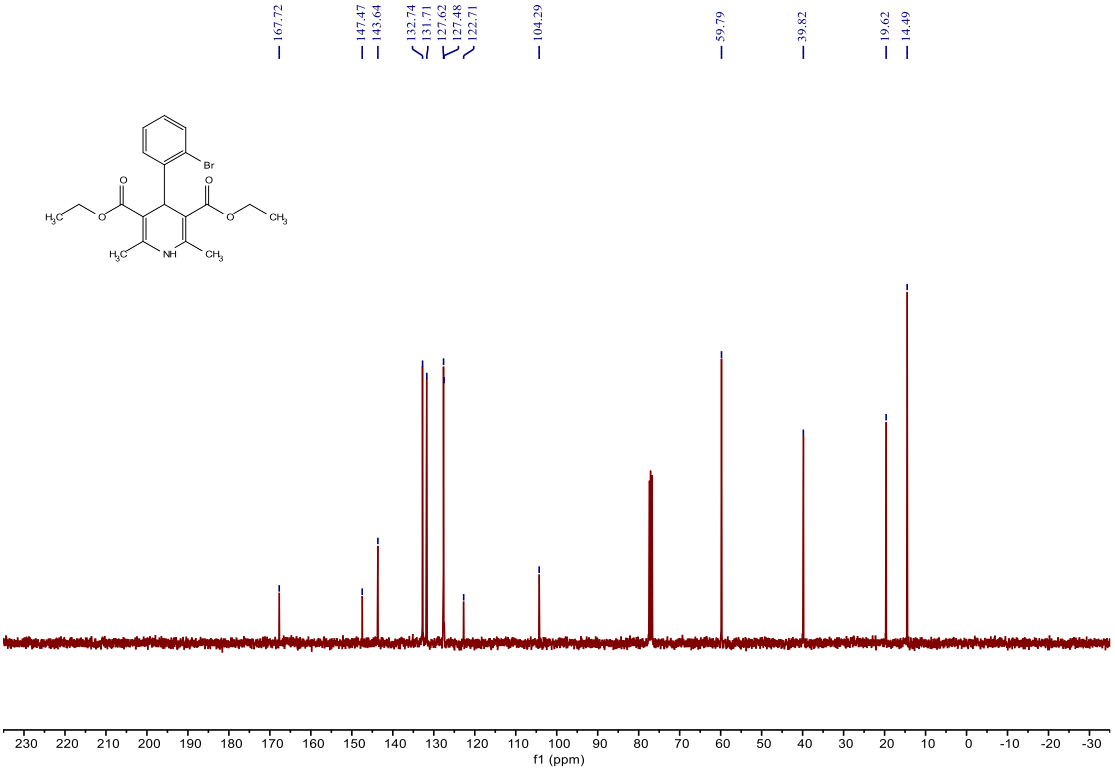

167.72
147.47
143.64
132.74
131.71
127.62
127.48
122.71
104.29
59.79
39.82
19.62
14.49
Br
O
O
H3C
O
O
CH3
H3C
NH
CH3
230
220
210
200
190
180
170
160
150
140
130
120
110
100
90
80
70
60
50
40
30
20
10
0
-10
-20
-30
f1 (ppm)

Compound **1q**

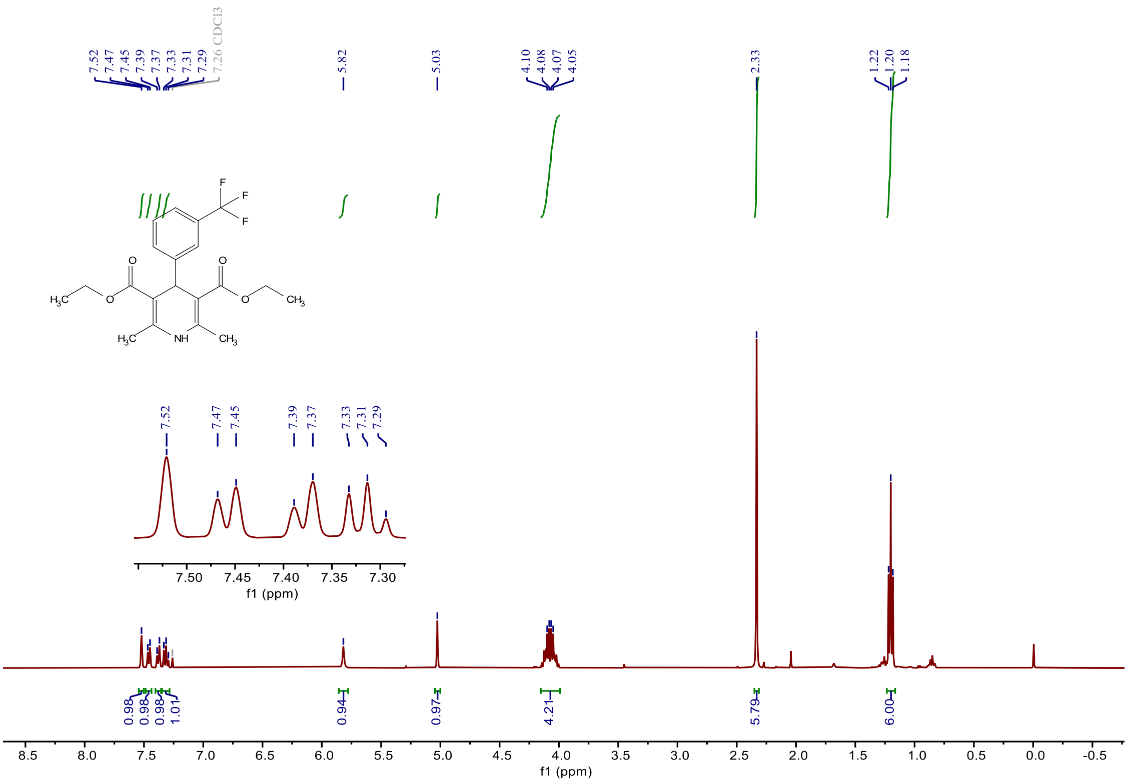

7.52
7.47
7.45
7.39
7.37
7.33
7.31
7.29
7.26 CDCl3
5.82
5.03
4.10
4.08
4.07
4.05
2.33
1.22
1.20
1.18
0.98
0.98
0.98
1.01
0.94
0.97
4.21
5.79
6.00
f1 (ppm)

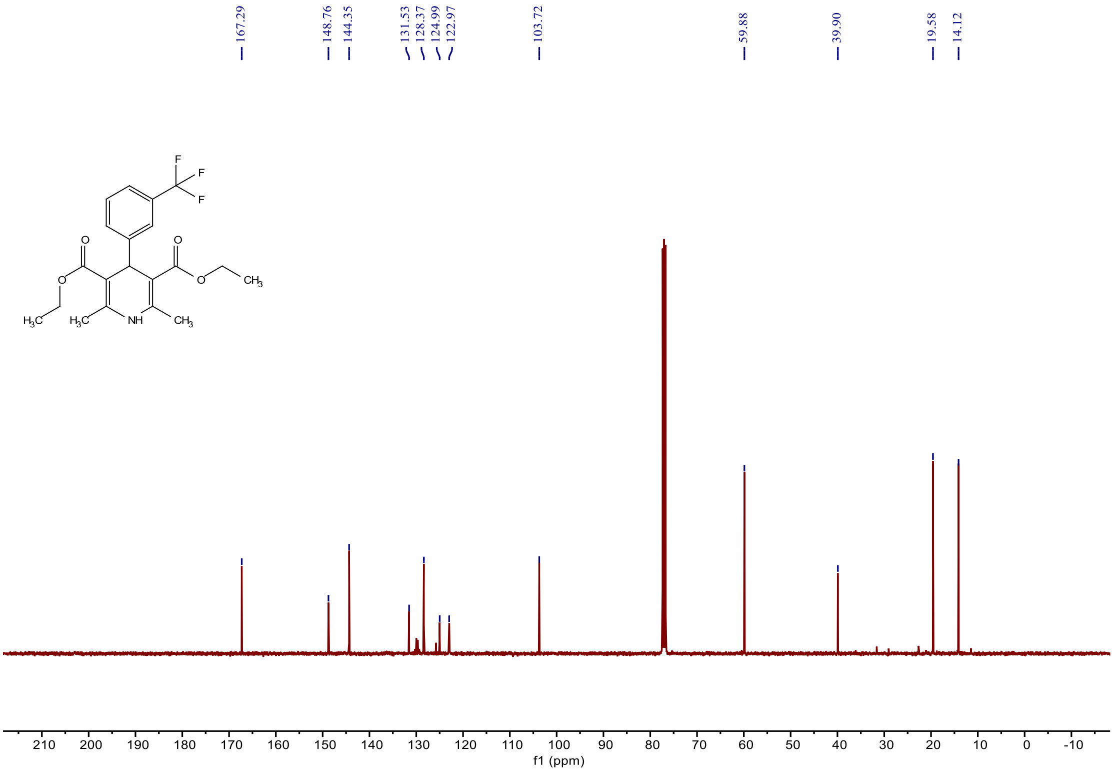

167.29
148.76
144.35
131.53
128.37
124.99
122.97
103.72
59.88
39.90
19.58
14.12
f1 (ppm)

Compound **1r**

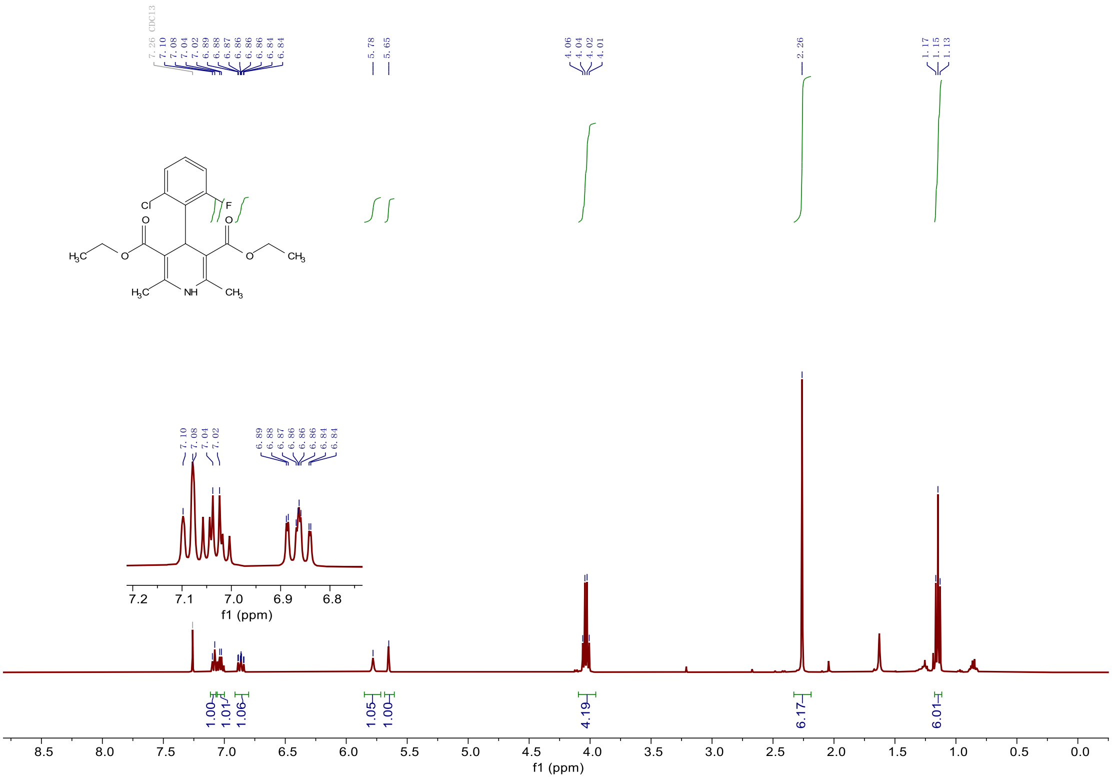

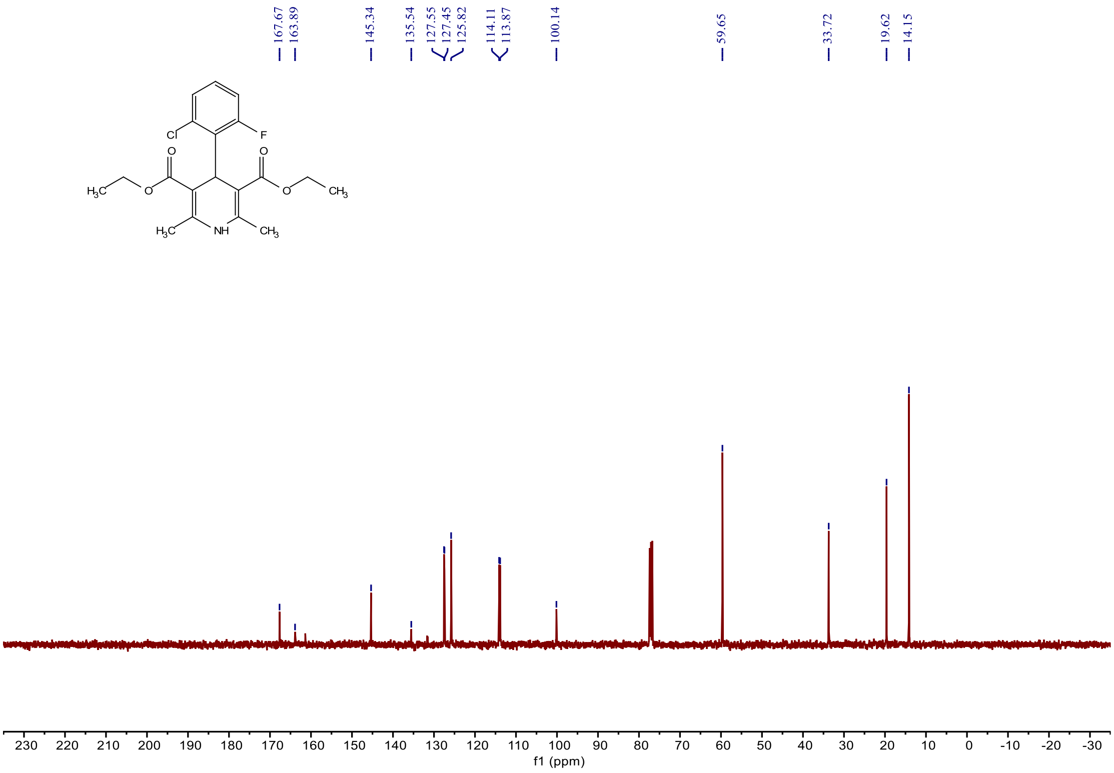

Compound **1s**

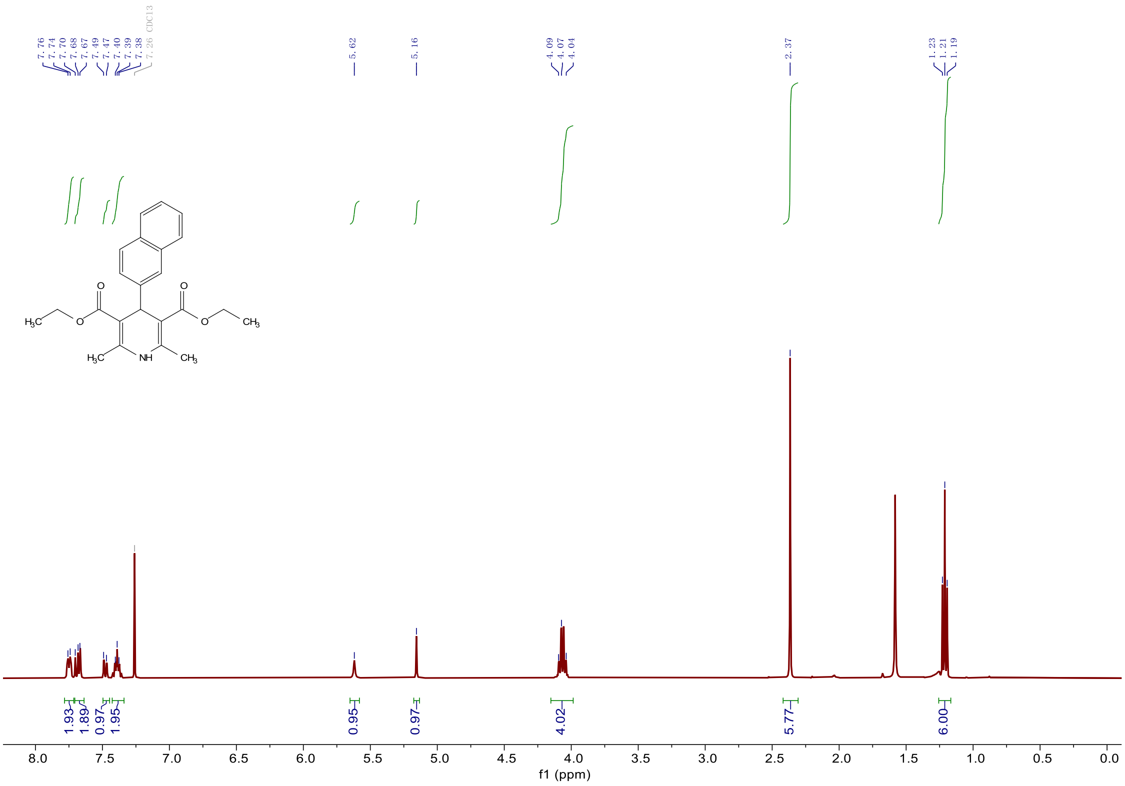

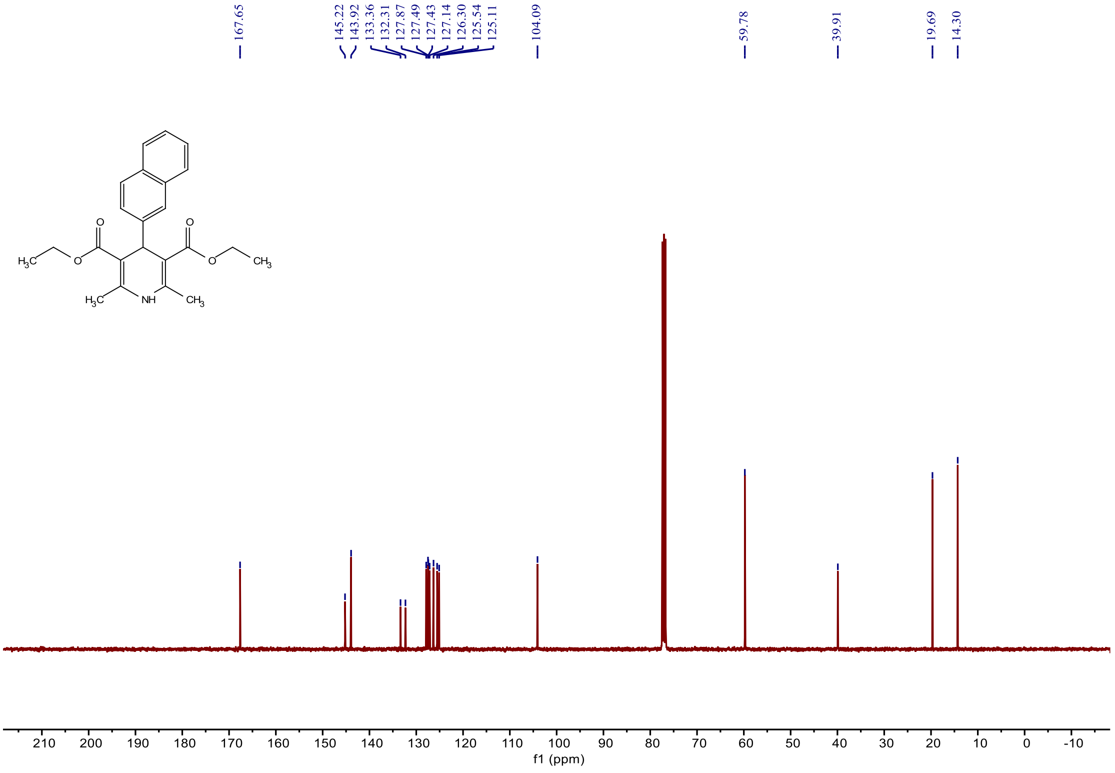

Compound **1t**

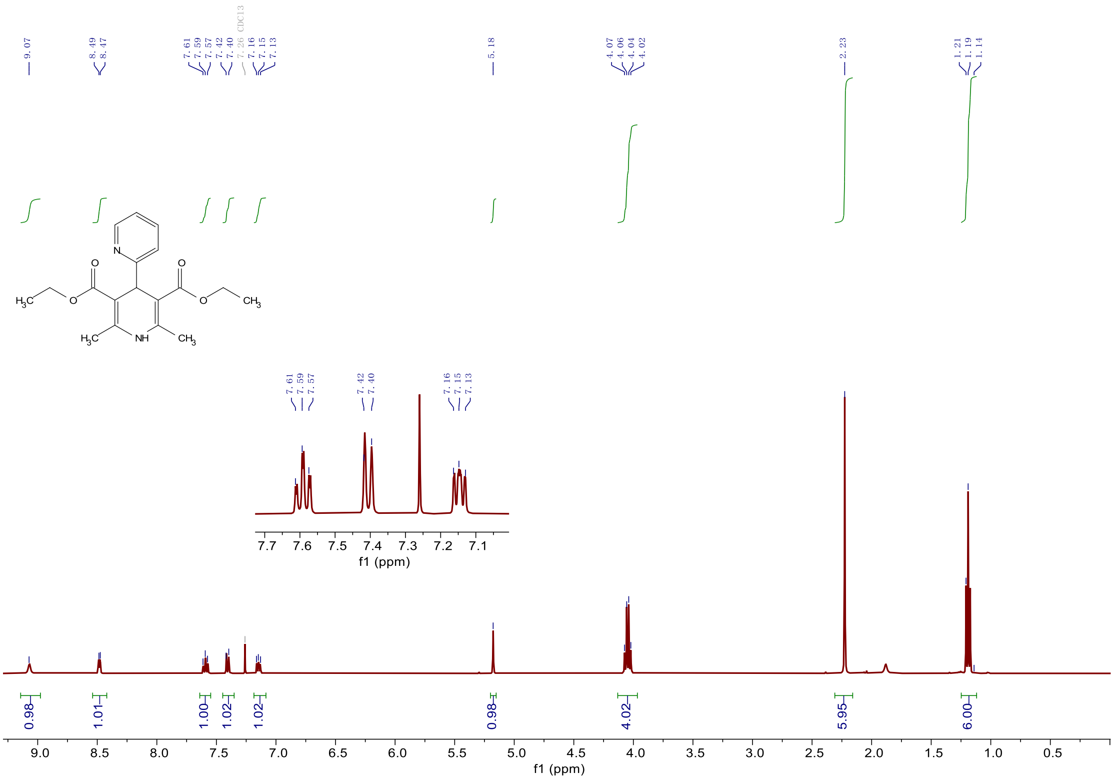

9.07
8.49
8.47
7.61
7.59
7.57
7.42
7.40
7.26 CDCl3
7.16
7.15
7.13
5.18
4.07
4.06
4.04
4.02
2.23
1.21
1.19
1.14
0.98
1.01
1.00
1.02
1.02
0.98
4.02
5.95
6.00
f1 (ppm)

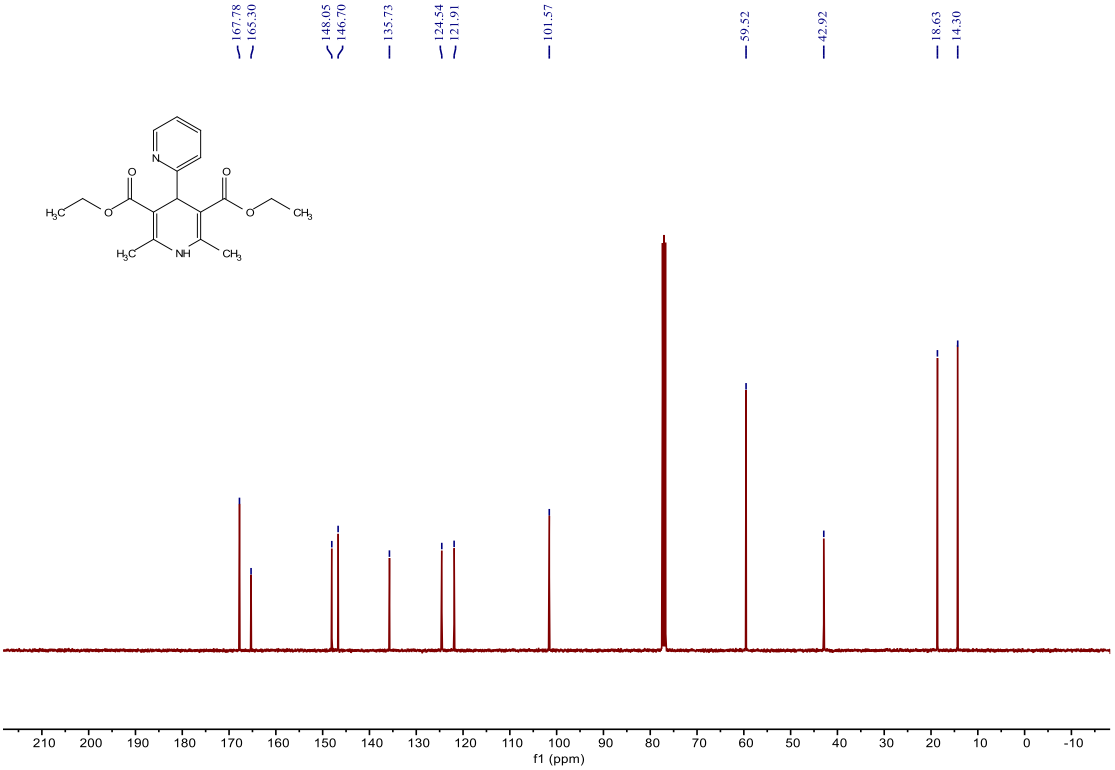

167.78
165.30
148.05
146.70
135.73
124.54
121.91
101.57
59.52
42.92
18.63
14.30
f1 (ppm)

Compound **1u**

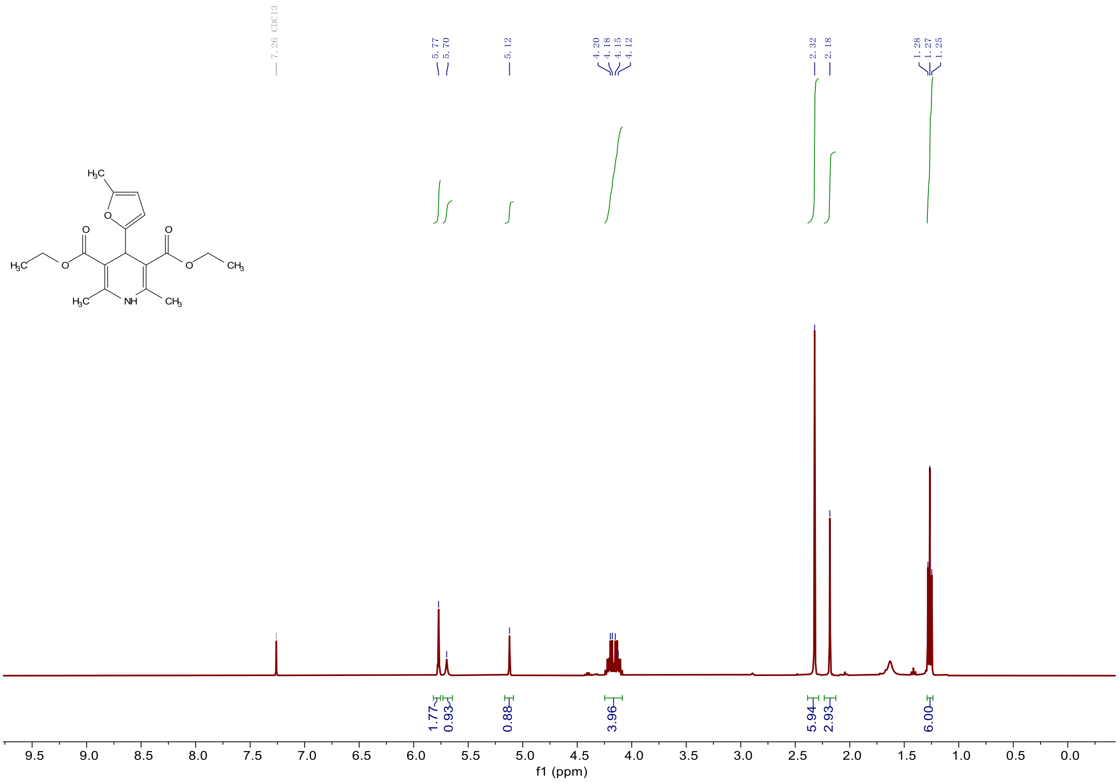
7.26 CDCl3
5.77
5.70
5.12
4.20
4.18
4.15
4.12
2.32
2.18
1.28
1.27
1.25
1.77
0.93
0.88
3.96
5.94
2.93
6.00
f1 (ppm)

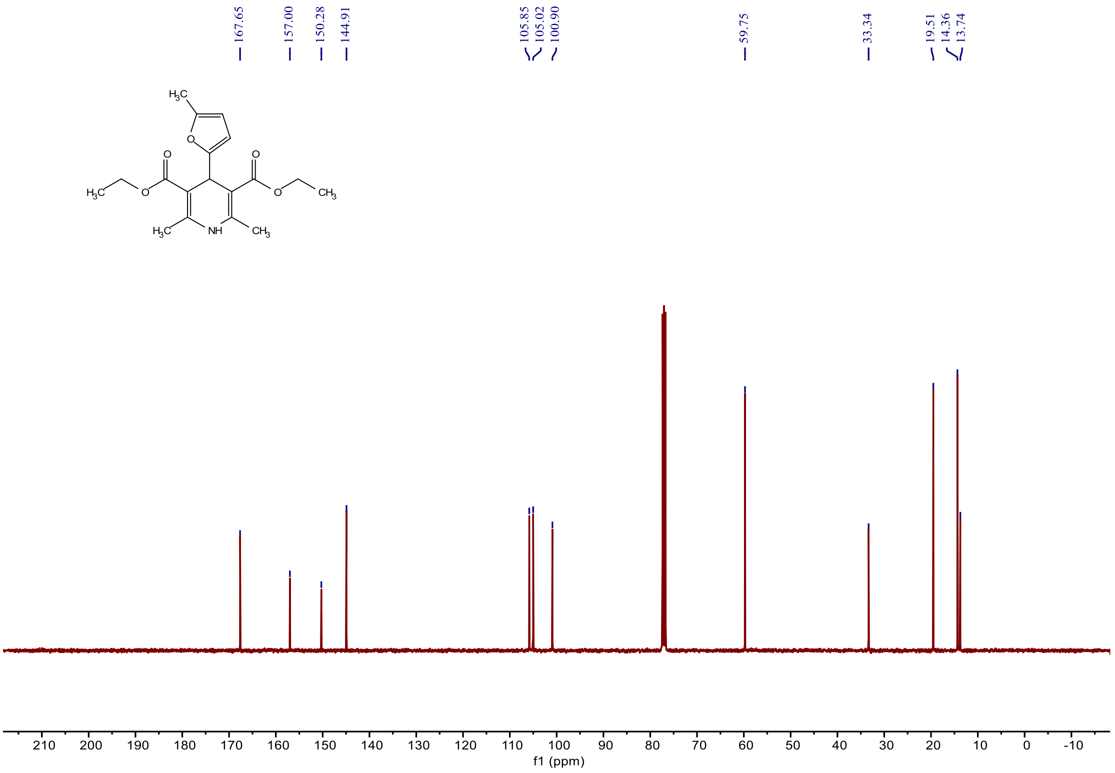
167.65
157.00
150.28
144.91
105.85
105.02
100.90
59.75
33.34
19.51
14.36
13.74
f1 (ppm)

Compound **1v**

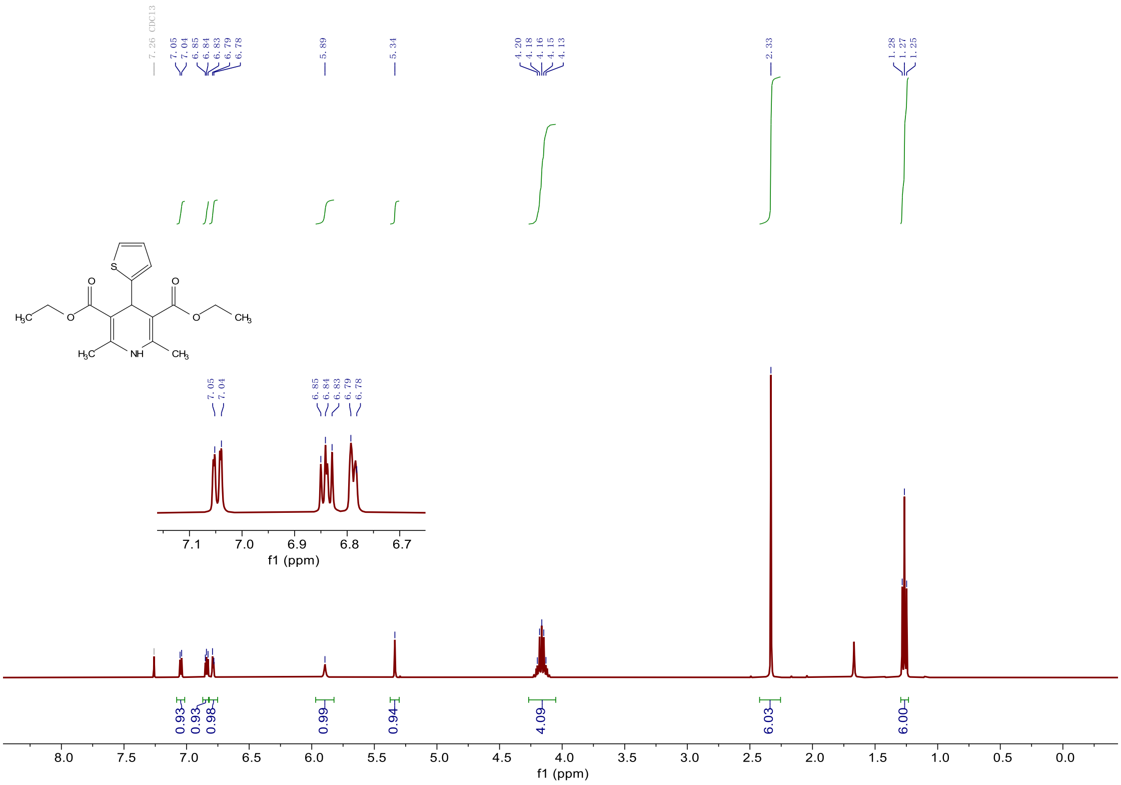

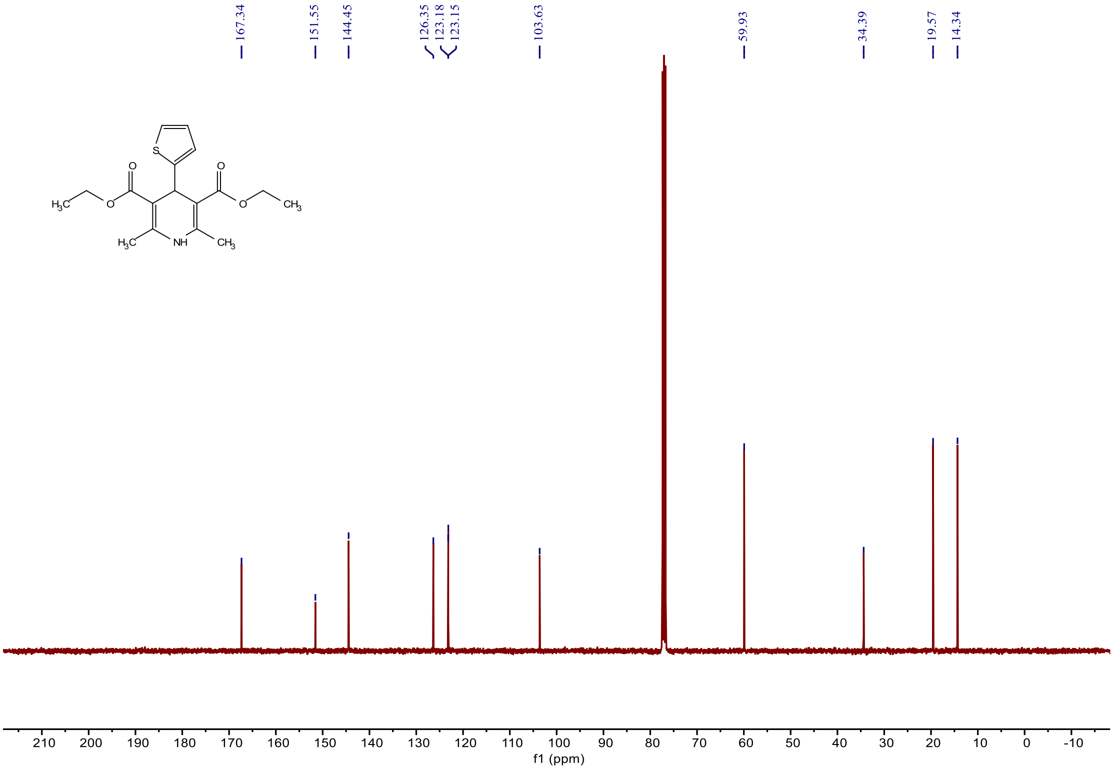

Compound **1w**

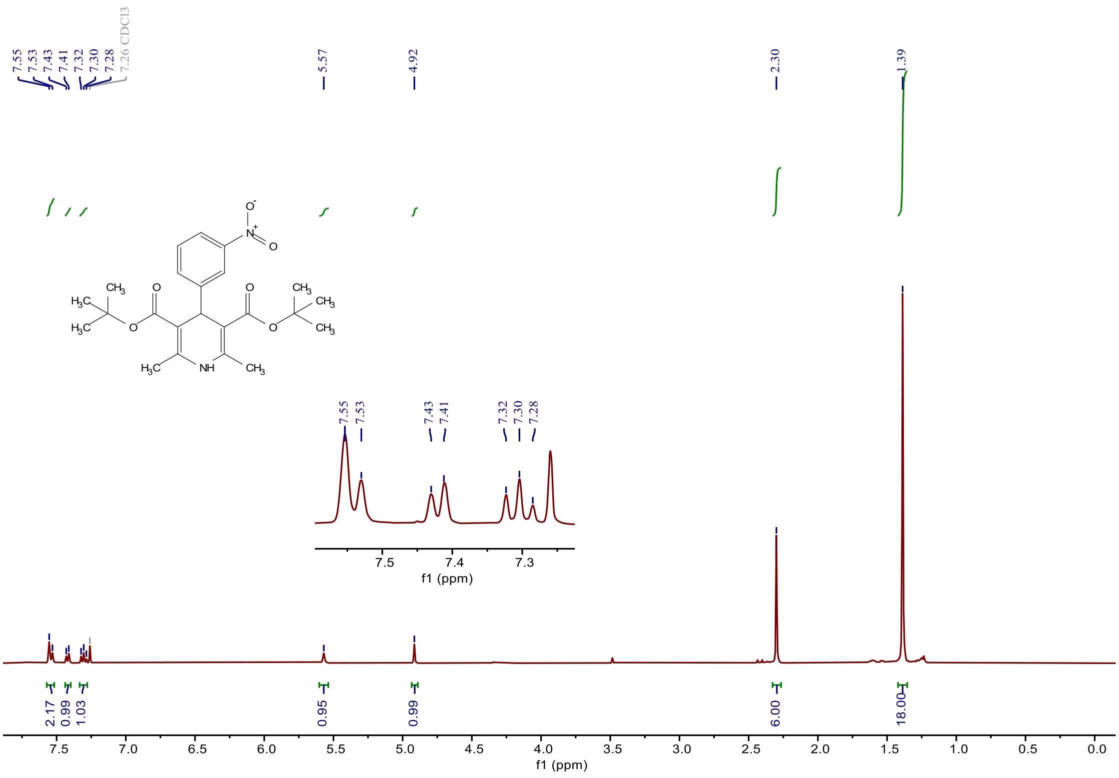
7.55
7.53
7.43
7.41
7.32
7.30
7.28
7.26 CDCl3
5.57
4.92
2.30
1.39
2.17
0.99
1.03
0.95
0.99
6.00
18.00
f1 (ppm)

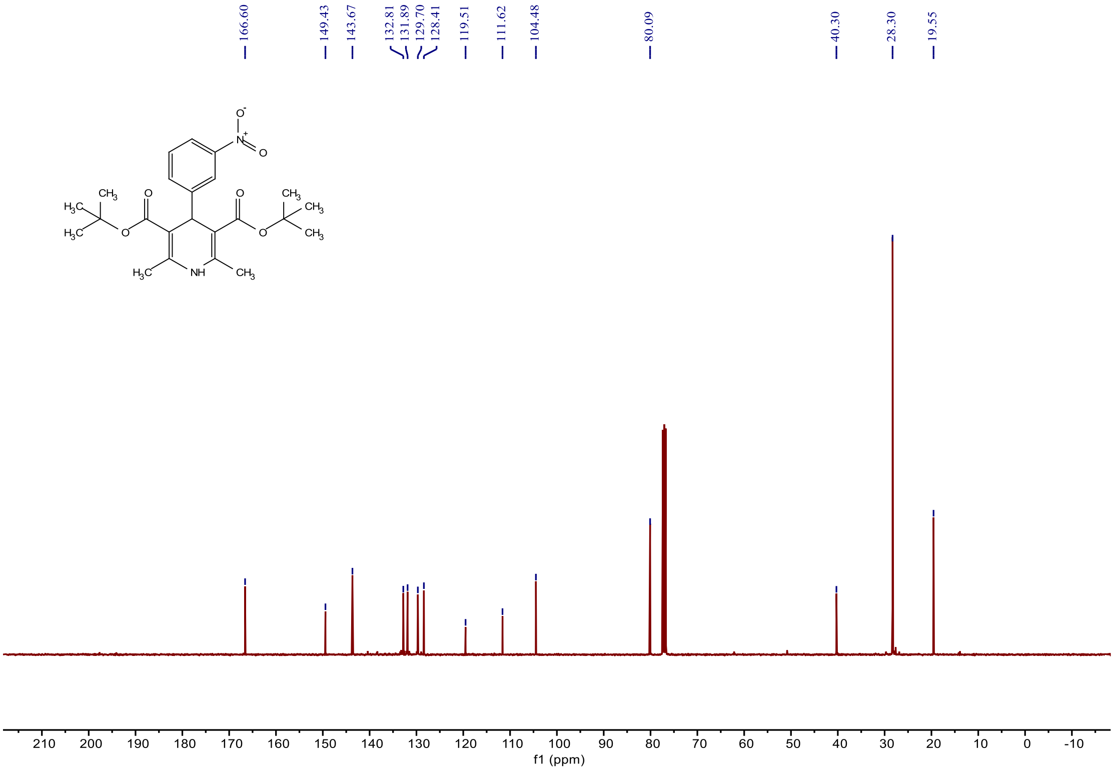
166.60
149.43
143.67
132.81
131.89
129.70
128.41
119.51
111.62
104.48
80.09
40.30
28.30
19.55
f1 (ppm)

Oxidative products:

Compound **2a**

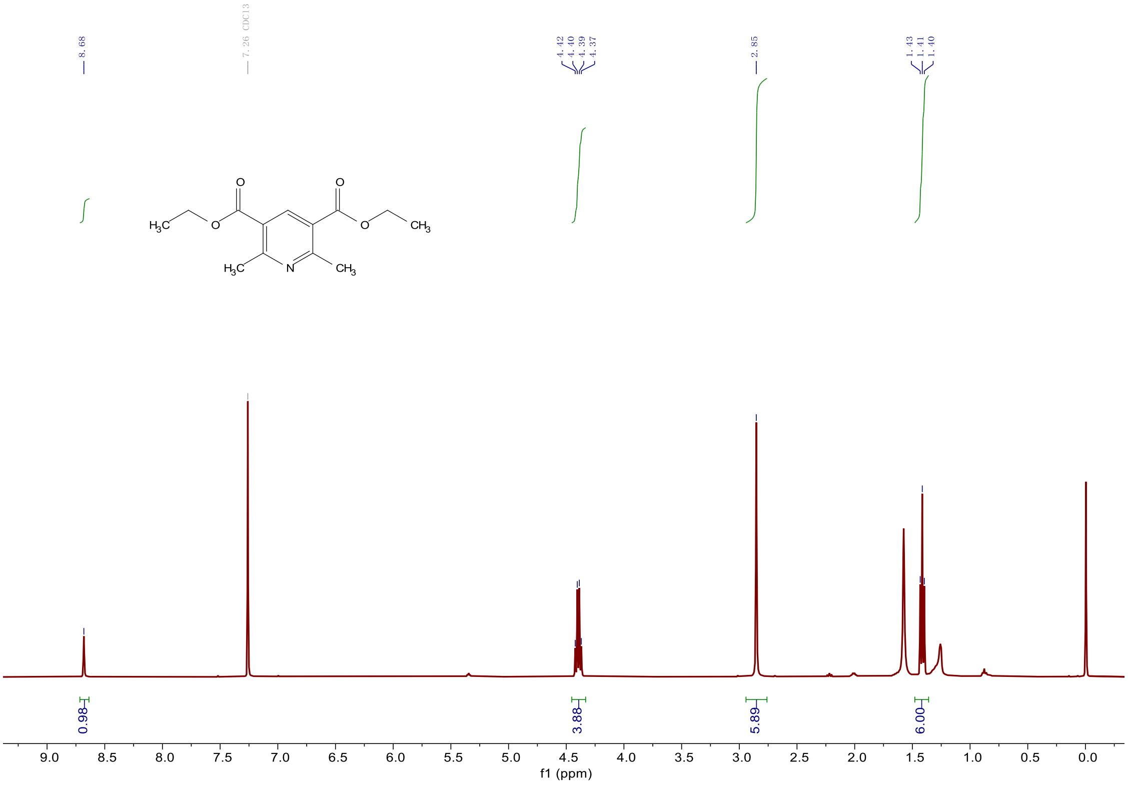

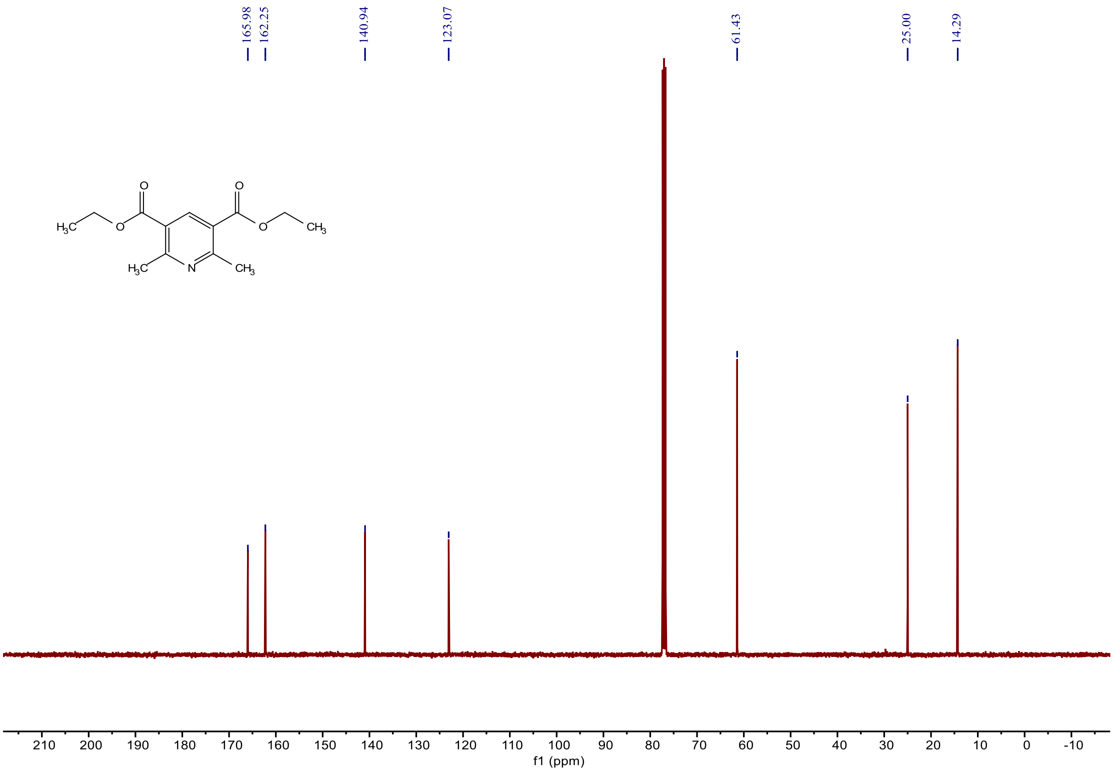

Compound **2b**

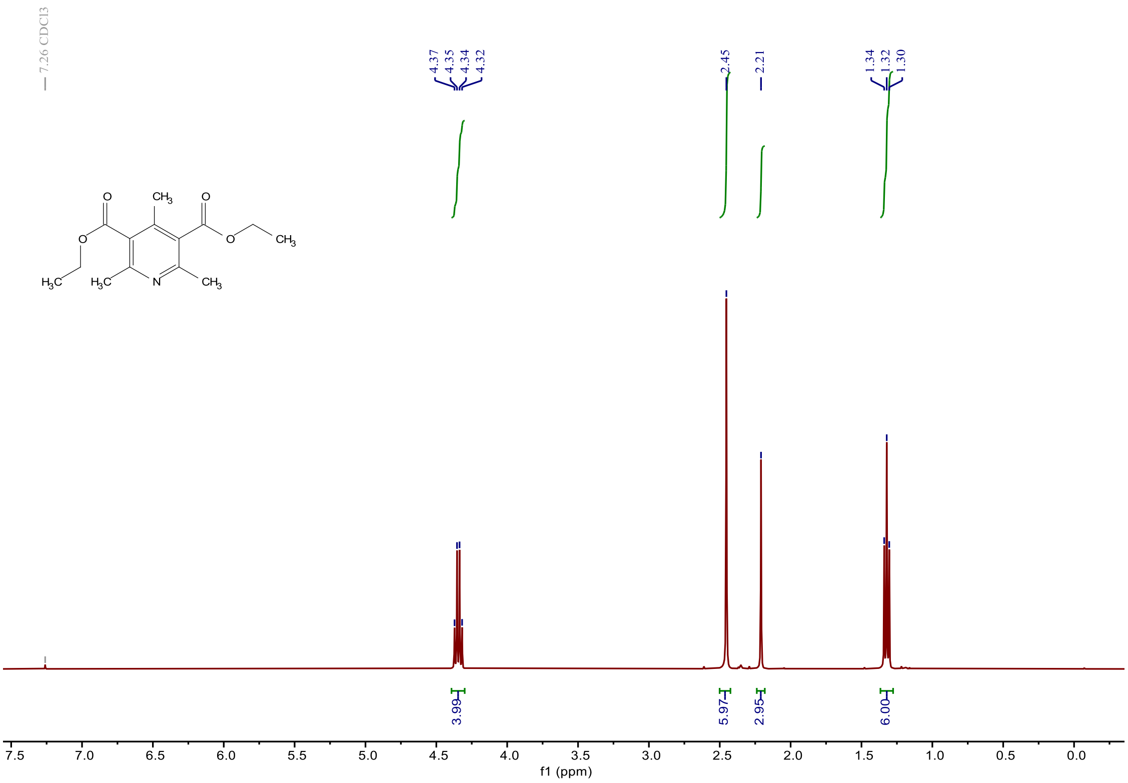
7.26 CDCl3
4.37
4.35
4.34
4.32
2.45
2.21
1.34
1.32
1.30
3.99
5.97
2.95
6.00
f1 (ppm)

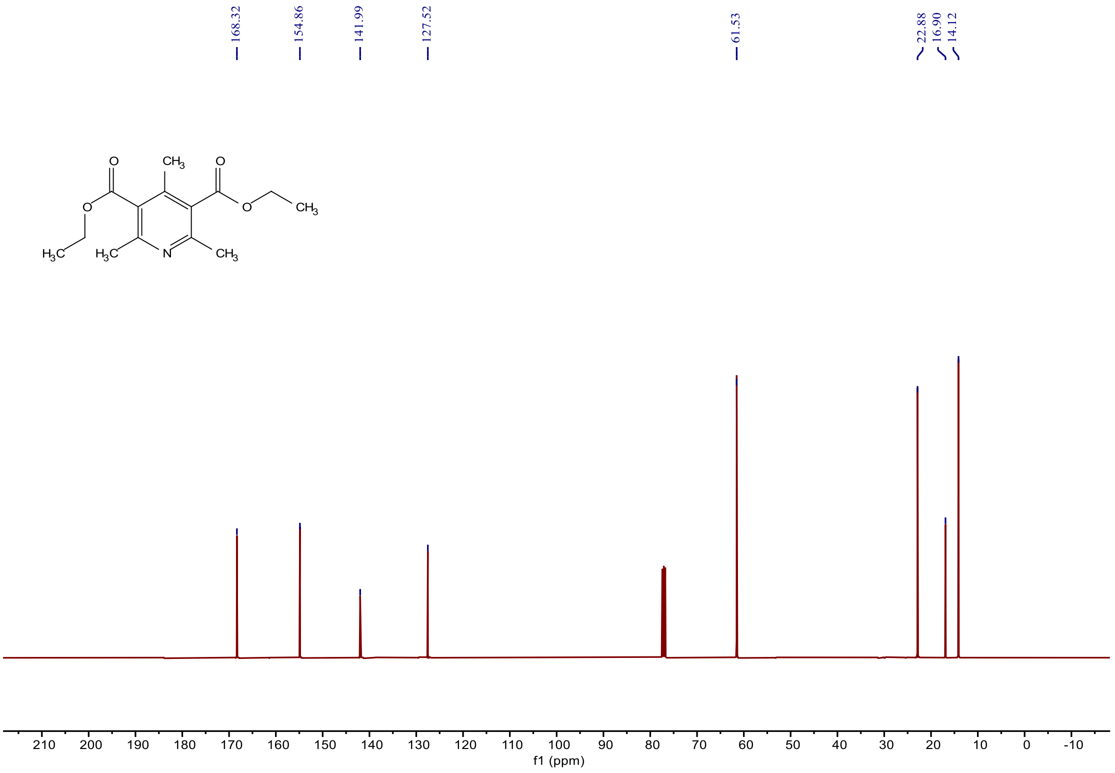
168.32
154.86
141.99
127.52
61.53
22.88
16.90
14.12
f1 (ppm)

Compound **2c**

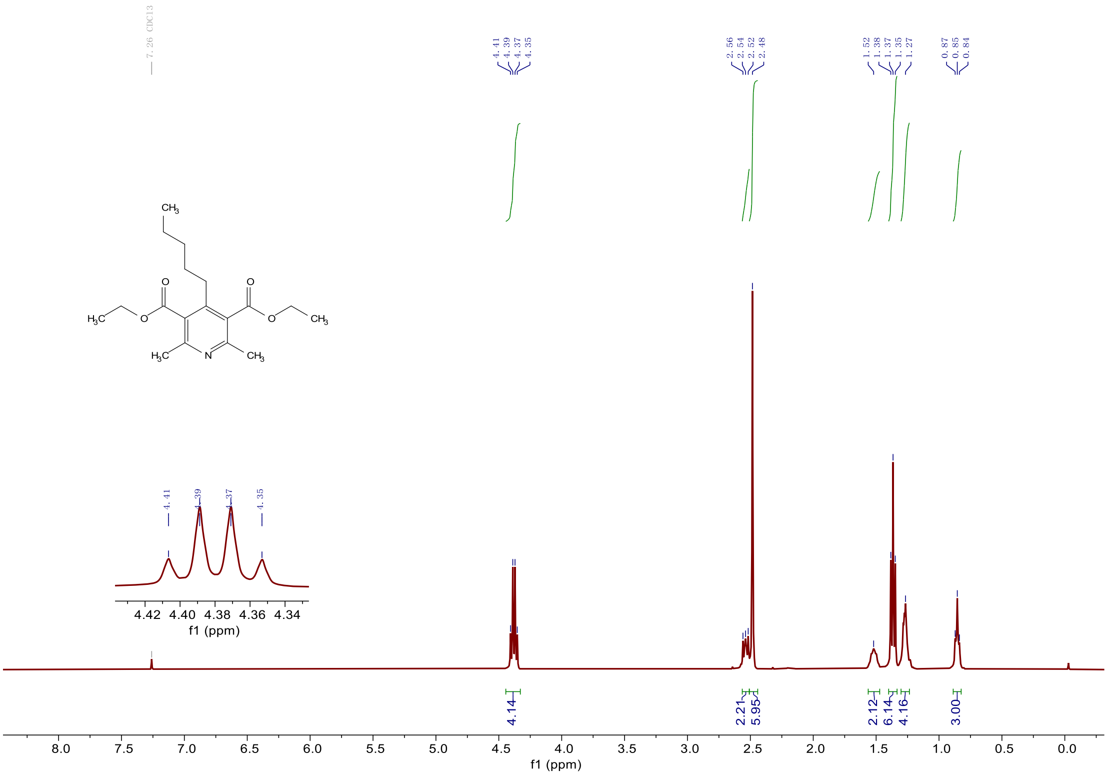
7.26 CDCl3
4.41
4.39
4.37
4.35
2.56
2.54
2.52
2.48
1.52
1.38
1.37
1.35
1.27
0.87
0.85
0.84
4.14
2.21
5.95
2.12
6.14
4.16
3.00
f1 (ppm)

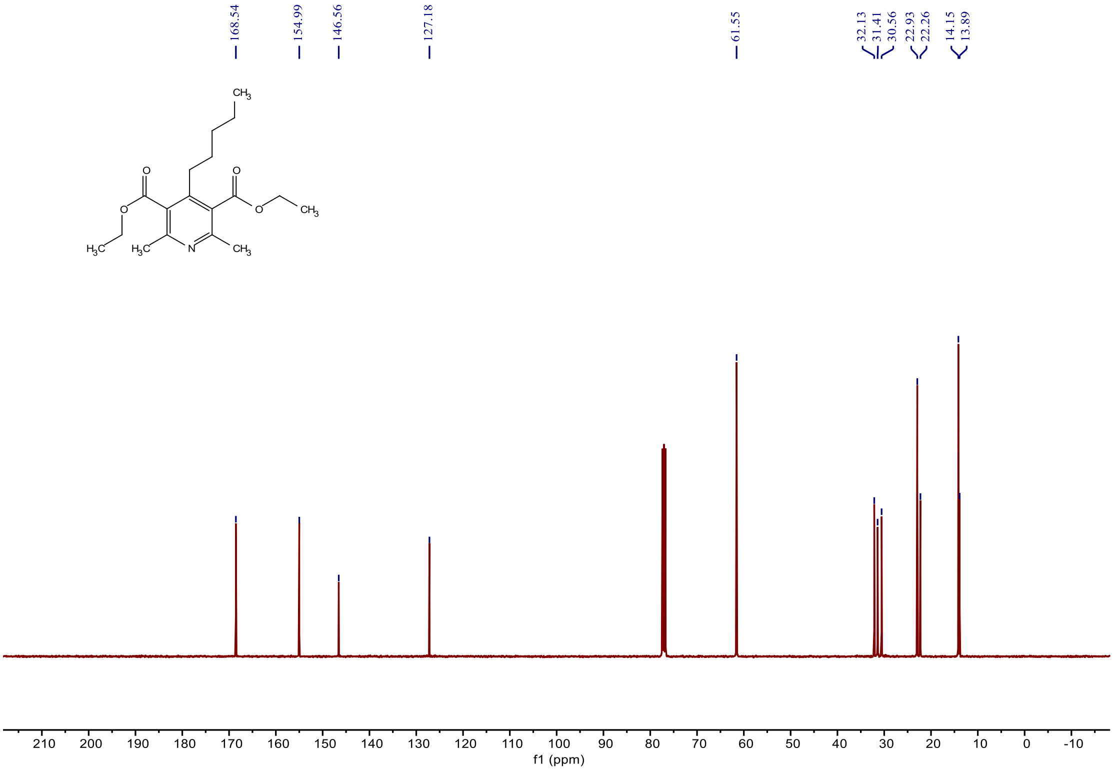
168.54
154.99
146.56
127.18
61.55
32.13
31.41
30.56
22.93
22.26
14.15
13.89
f1 (ppm)

Compound **2d**

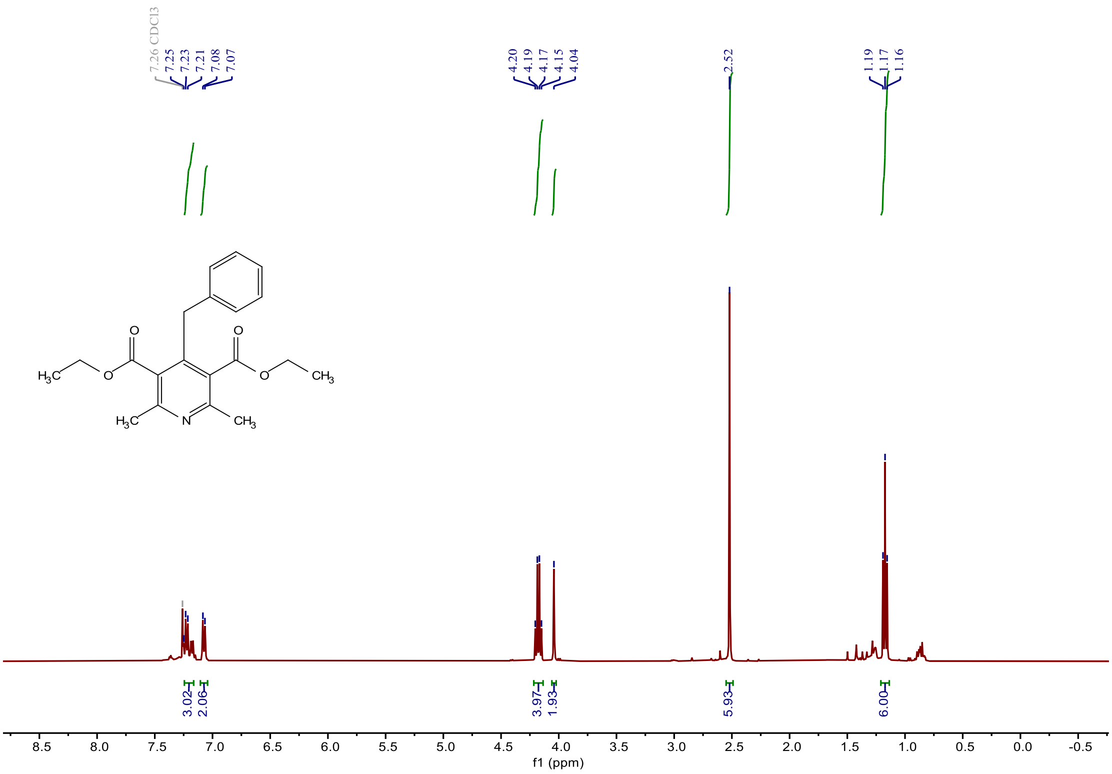

7.26 CDCl3
7.25
7.23
7.21
7.08
7.07
4.20
4.19
4.17
4.15
4.04
2.52
1.19
1.17
1.16
3.02
2.06
3.97
1.93
5.93
6.00
f1 (ppm)

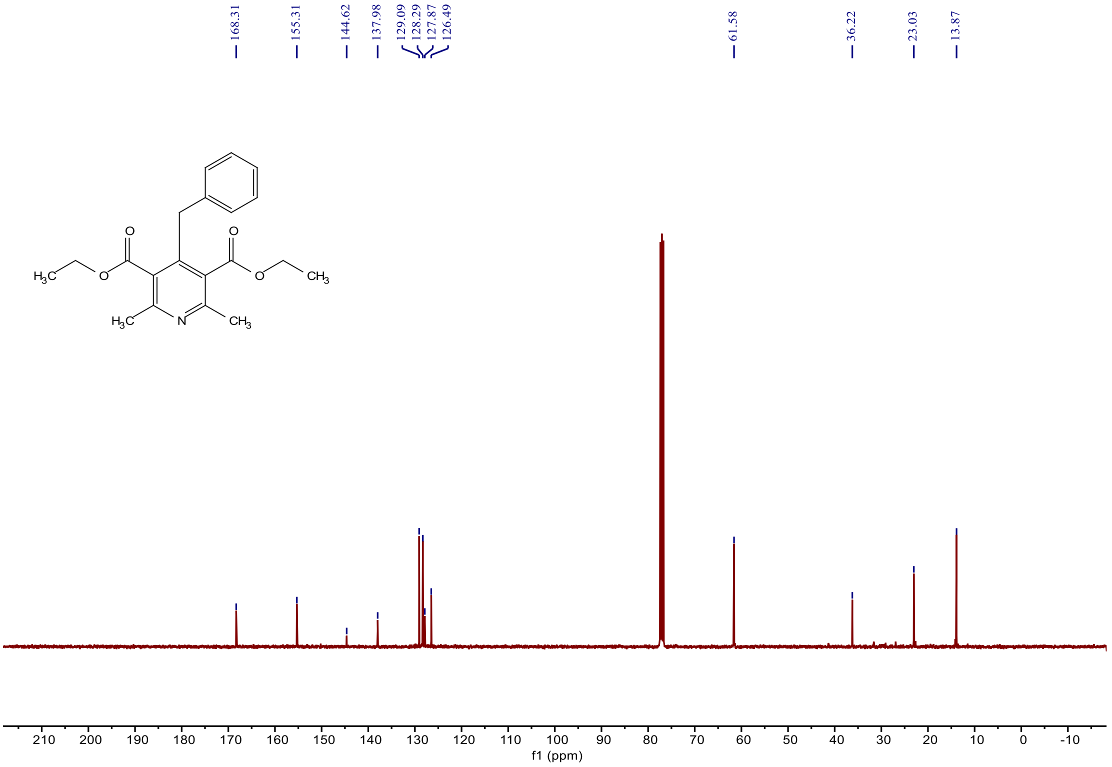

168.31
155.31
144.62
137.98
129.09
128.29
127.87
126.49
61.58
36.22
23.03
13.87
f1 (ppm)

Compound **2e**

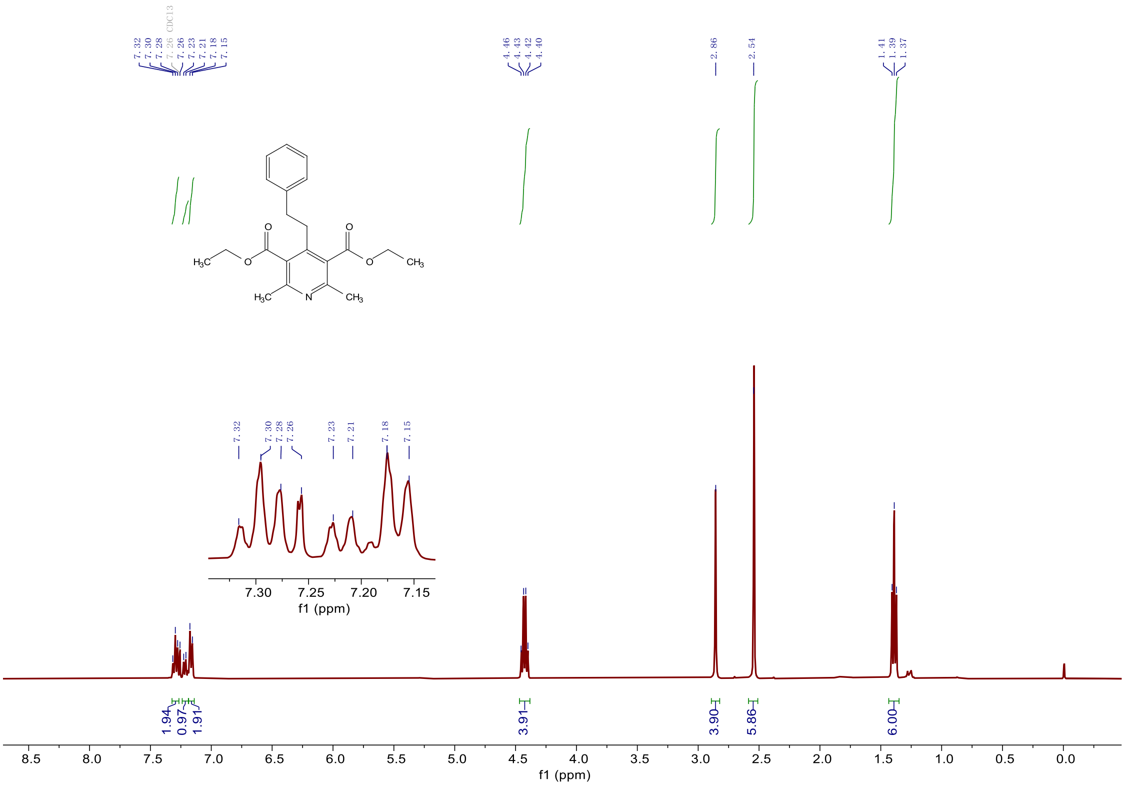

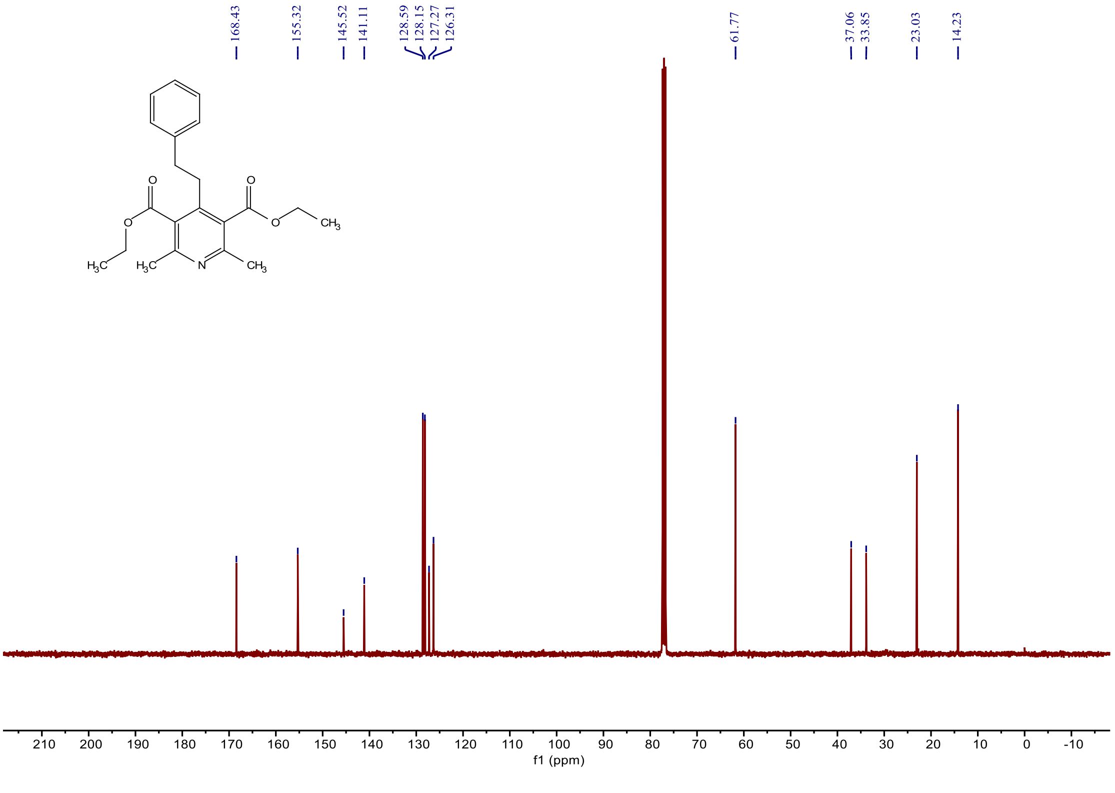

Compound **2f**

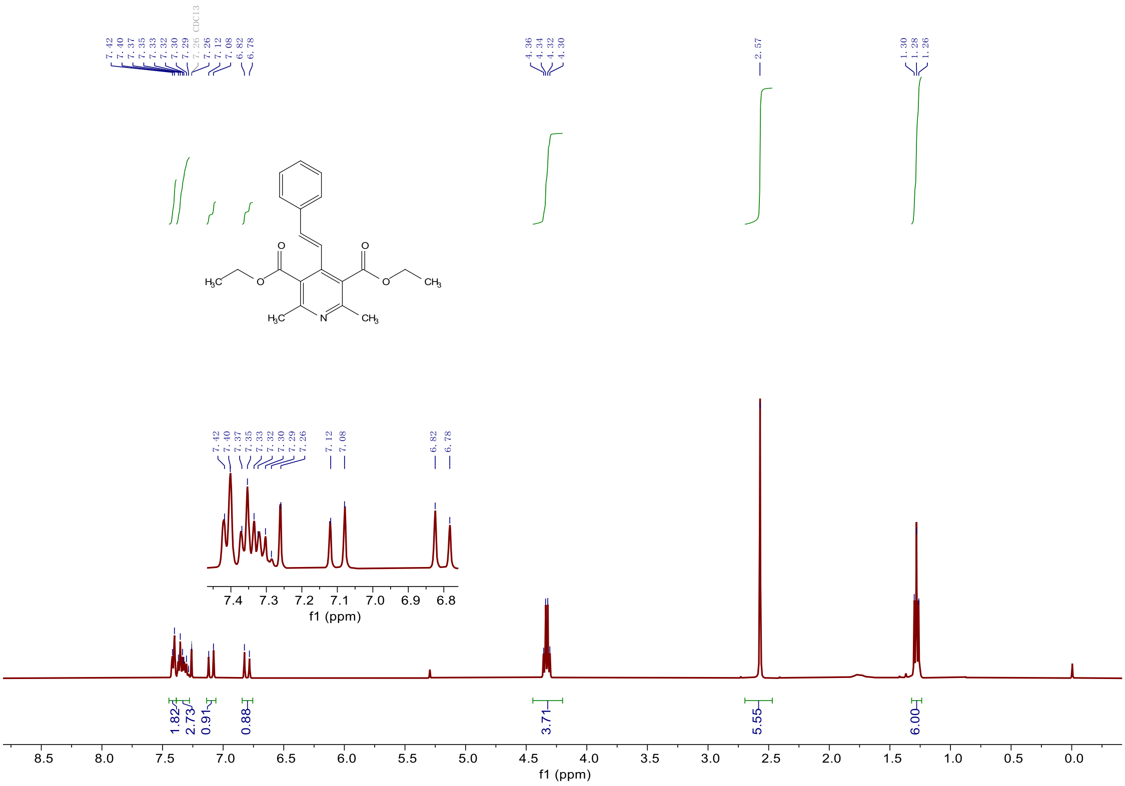
7.42 7.40 7.37 7.35 7.33 7.32 7.30 7.29 7.26 CDCl3 7.26 7.12 7.08 6.82 6.78
4.36 4.34 4.32 4.30
2.57
1.30 1.28 1.26
1.82 2.73 0.91 0.88 3.71 5.55 6.00
f1 (ppm)

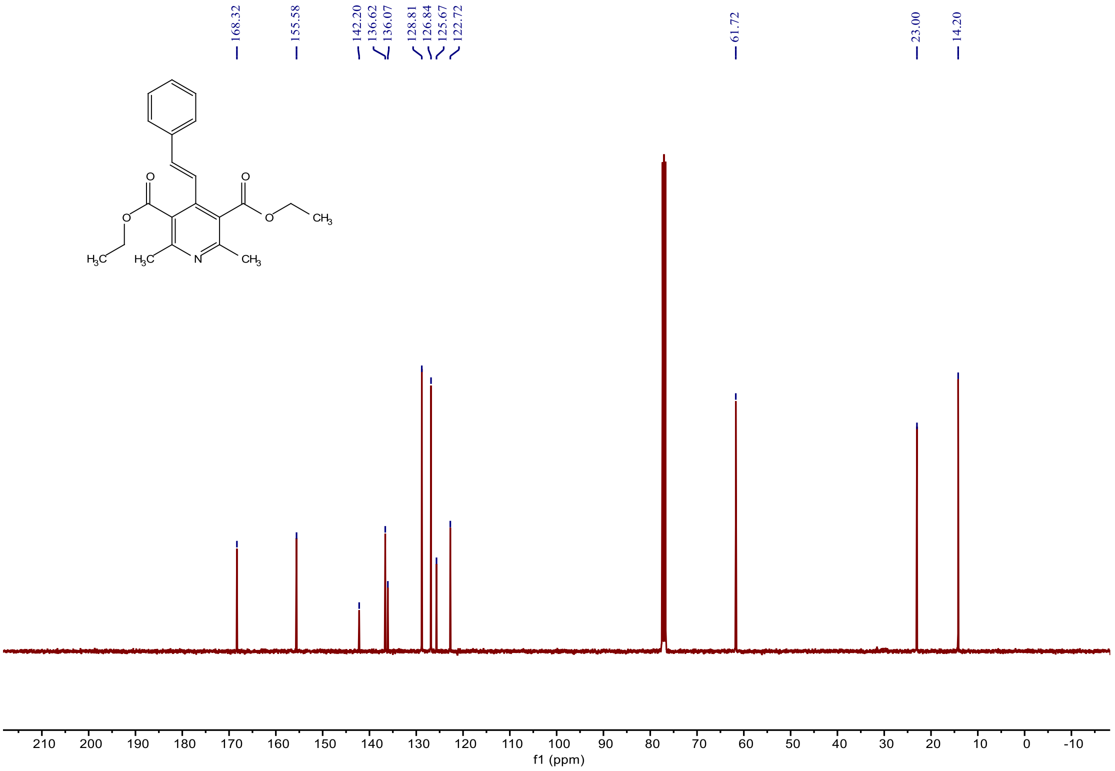
168.32
155.58
142.20 136.62 136.07
128.81 126.84 125.67 122.72
61.72
23.00
14.20
f1 (ppm)

Compound **2g**

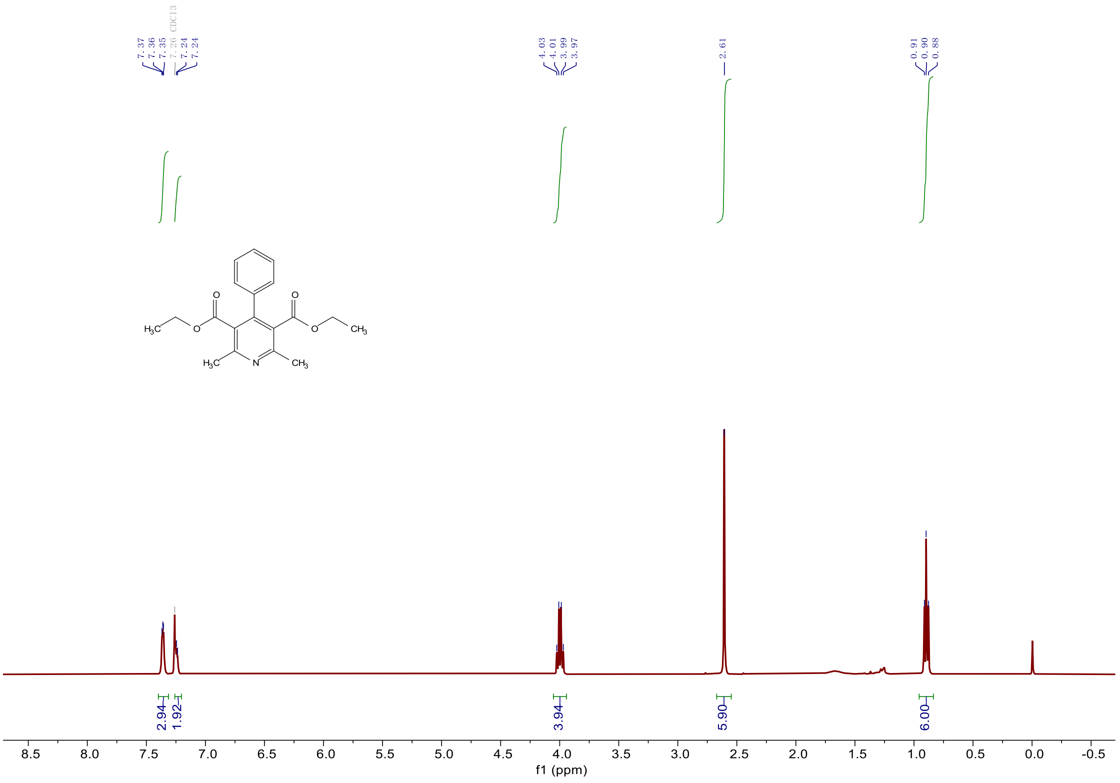

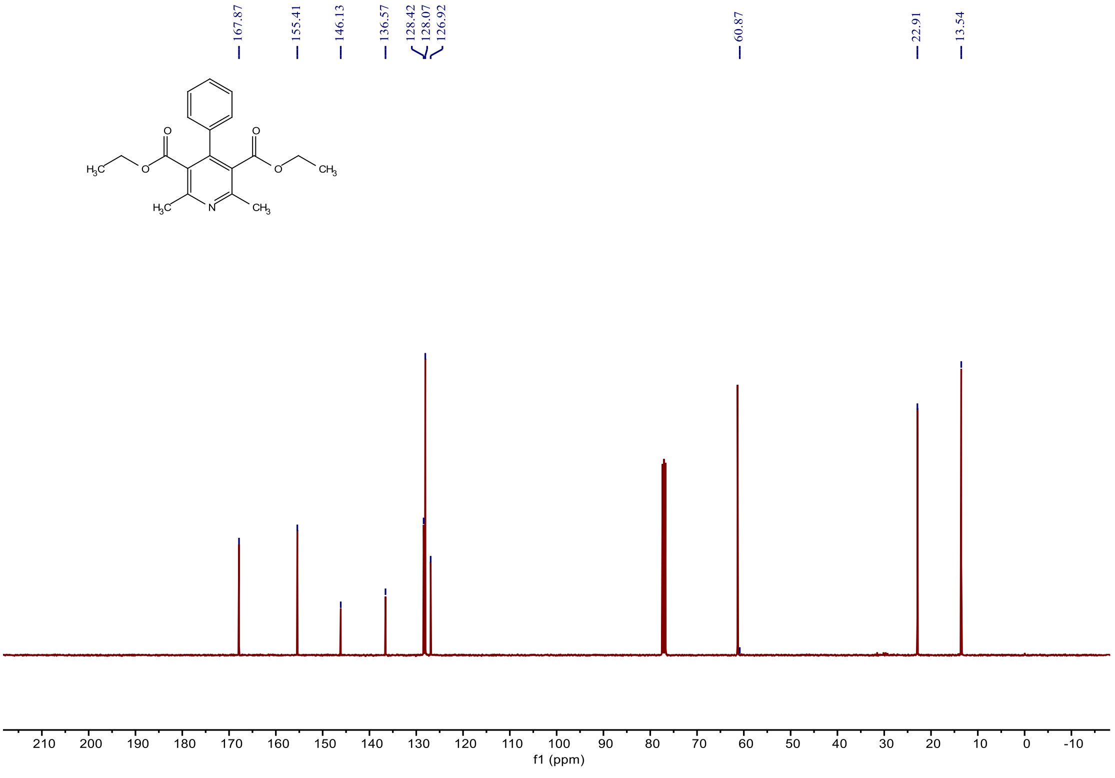

Compound **2h**

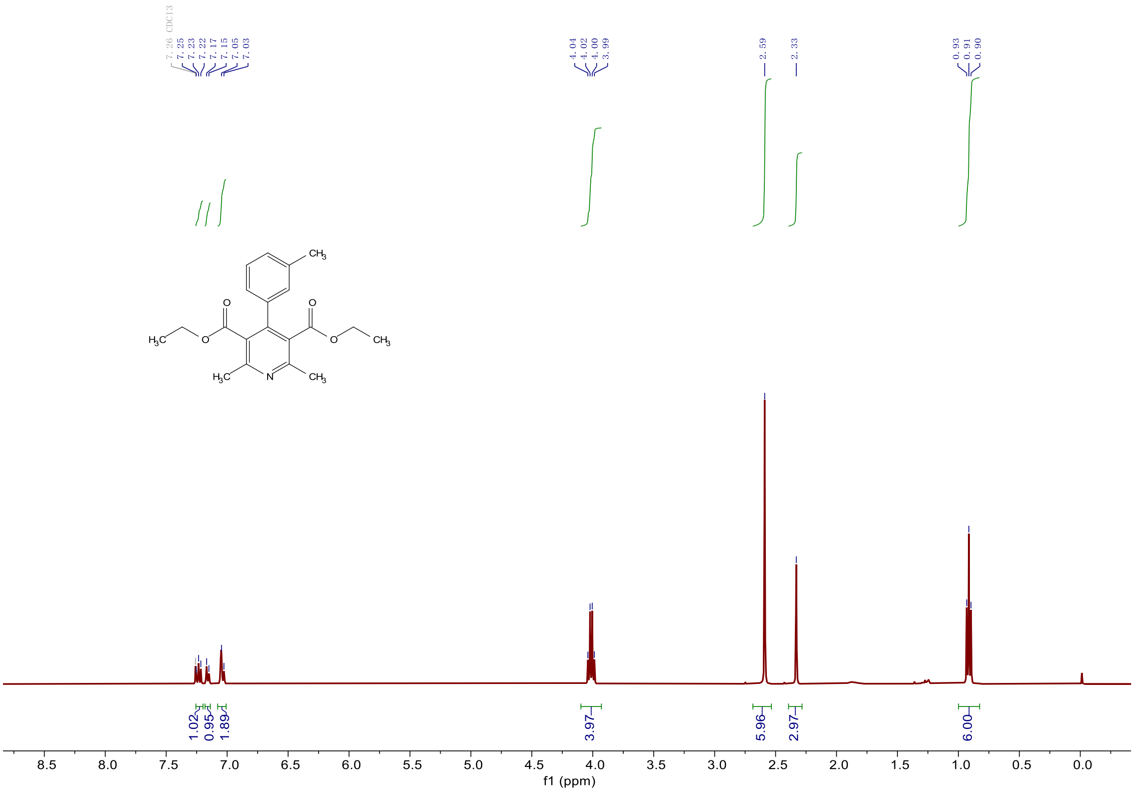
7.26 CDCl3
7.25
7.23
7.22
7.17
7.15
7.05
7.03
4.04
4.02
4.00
3.99
2.59
2.33
0.93
0.91
0.90
1.02
0.95
1.89
3.97
5.96
2.97
6.00
f1 (ppm)

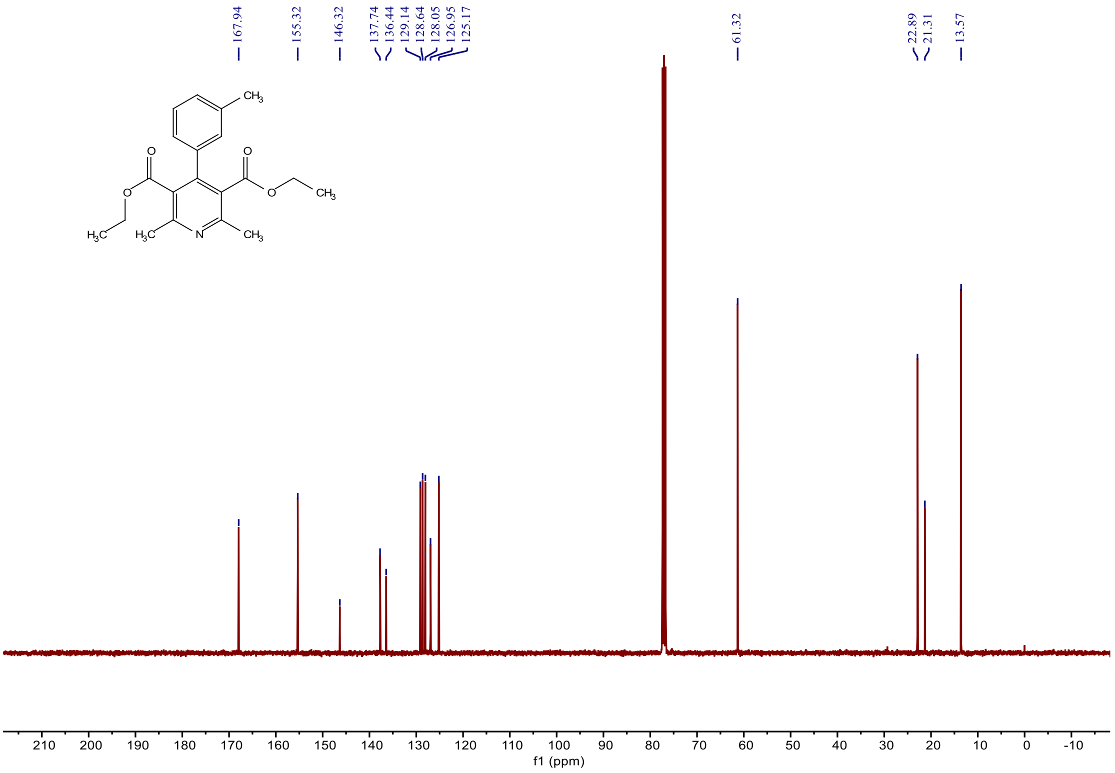
167.94
155.32
146.32
137.74
136.44
129.14
128.64
128.05
126.95
125.17
61.32
22.89
21.31
13.57
f1 (ppm)

Compound **2i**

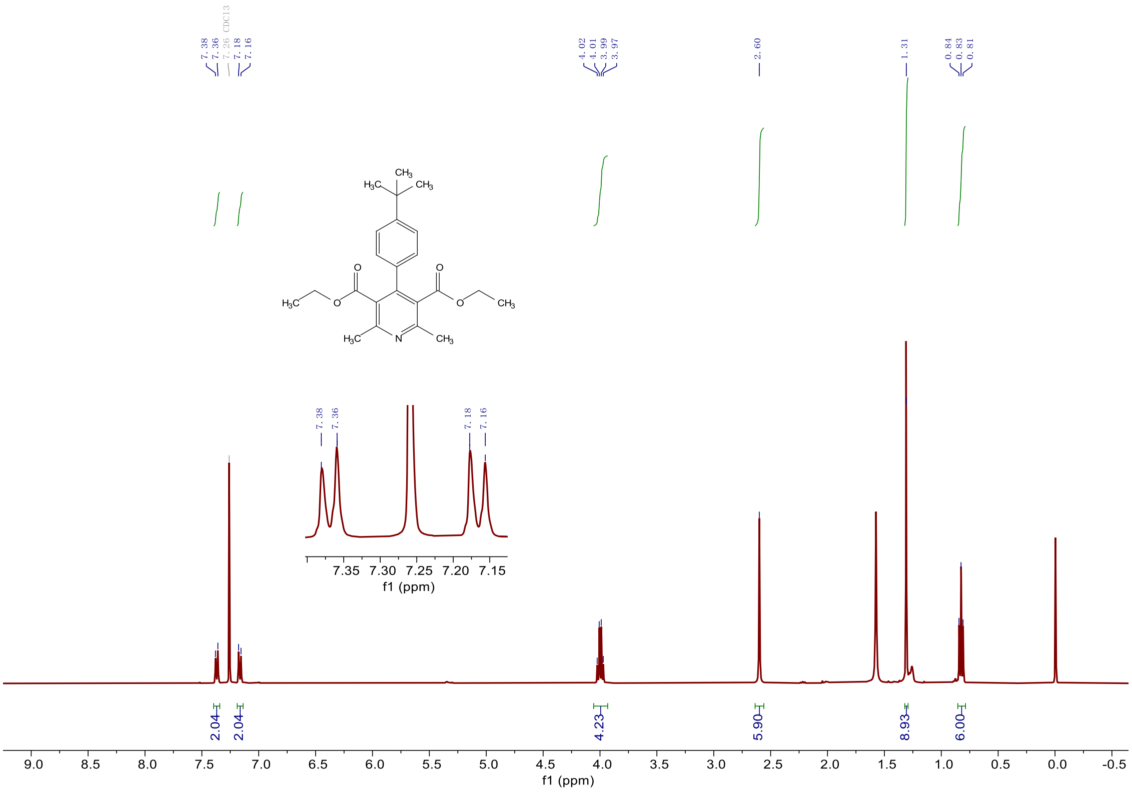
7.38
7.36
7.26 CDCl3
7.18
7.16
4.02
4.01
3.99
3.97
2.60
1.31
0.84
0.83
0.81
2.04
2.04
4.23
5.90
8.93
6.00
f1 (ppm)

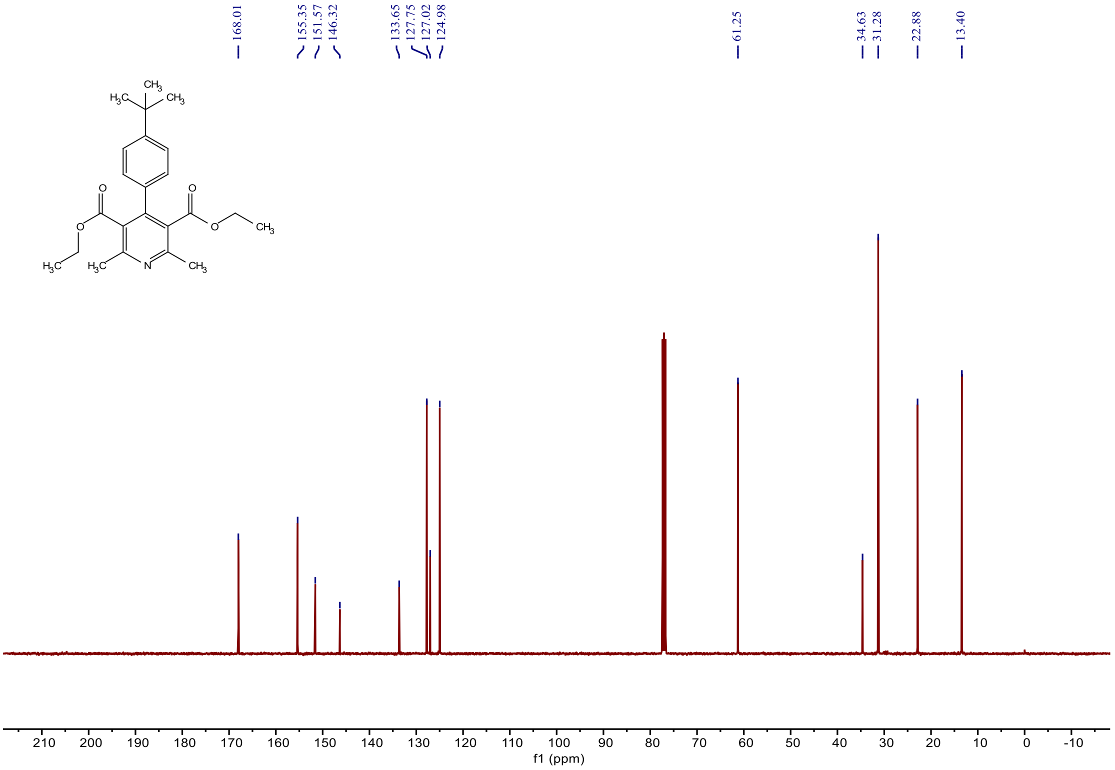
168.01
155.35
151.57
146.32
133.65
127.75
127.02
124.98
61.25
34.63
31.28
22.88
13.40
f1 (ppm)

Compound **2j**

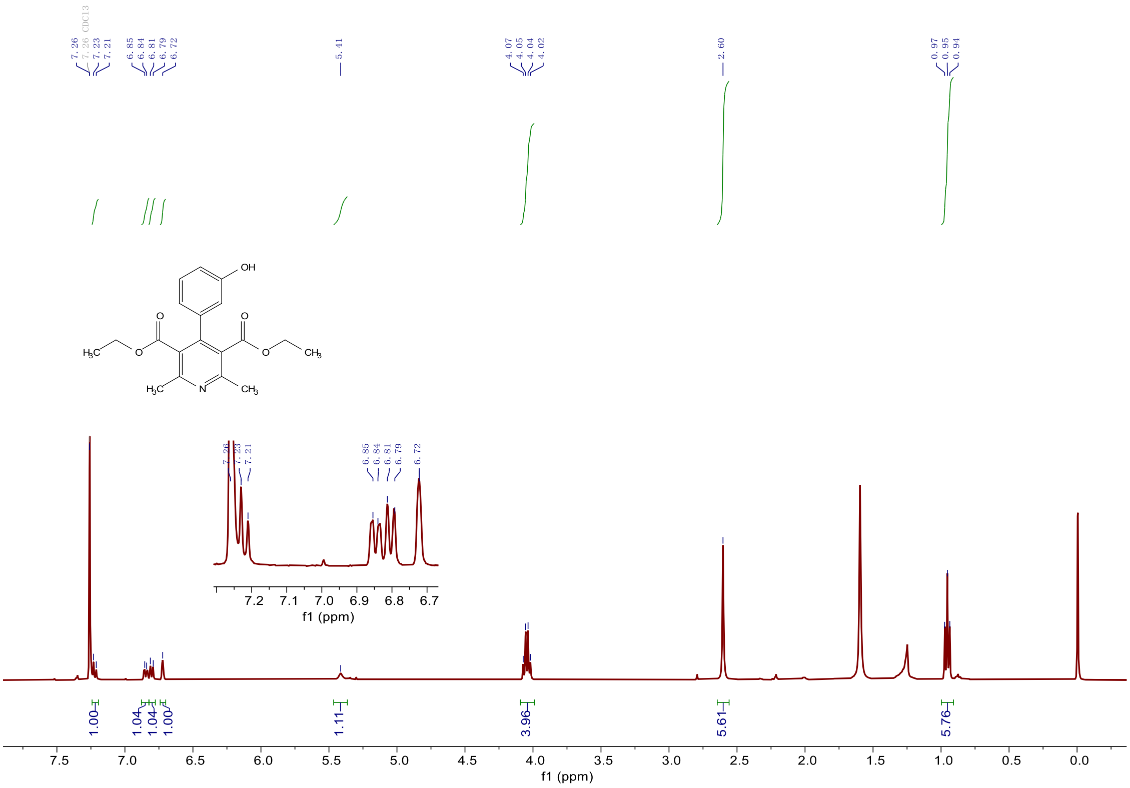
7.26
7.26 CDCl3
7.23
7.21
6.85
6.84
6.81
6.79
6.72
5.41
4.07
4.05
4.04
4.02
2.60
0.97
0.95
0.94
OH
O
O
H3C
O
O
CH3
H3C
N
CH3
1.00
1.04
1.04
1.00
1.11
3.96
5.61
5.76
f1 (ppm)

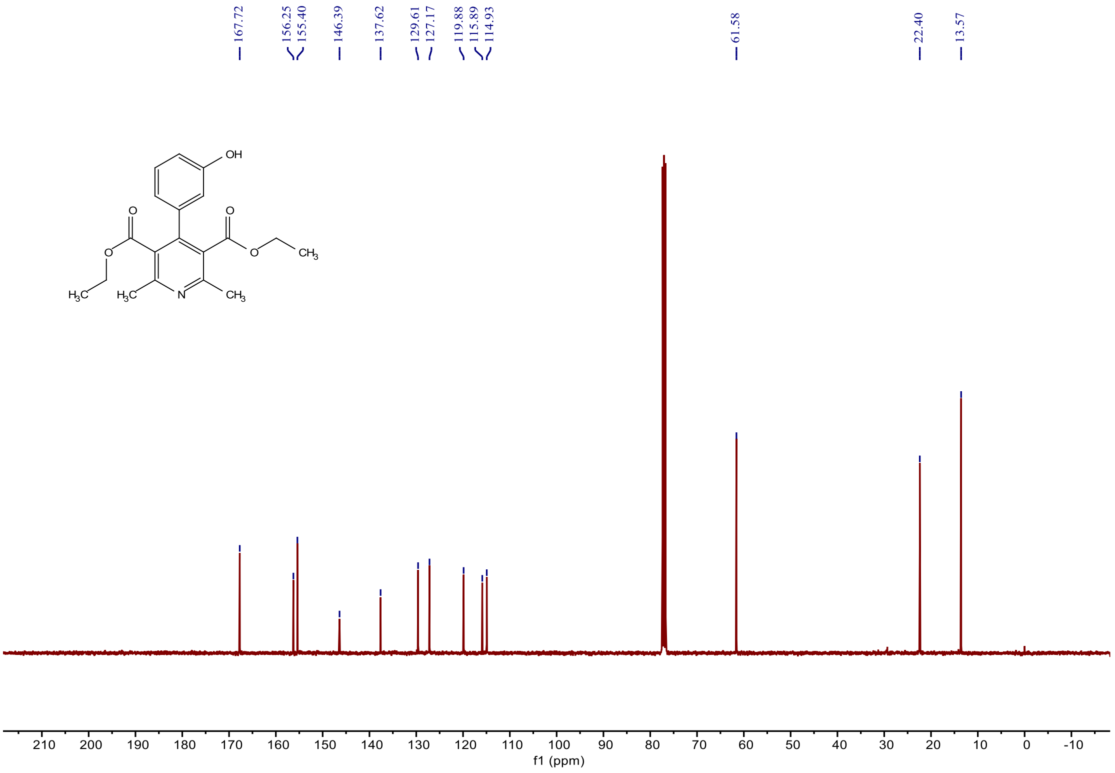
167.72
156.25
155.40
146.39
137.62
129.61
127.17
119.88
115.89
114.93
61.58
22.40
13.57
OH
O
O
O
O
CH3
H3C
H3C
N
CH3
f1 (ppm)

Compound **2k**

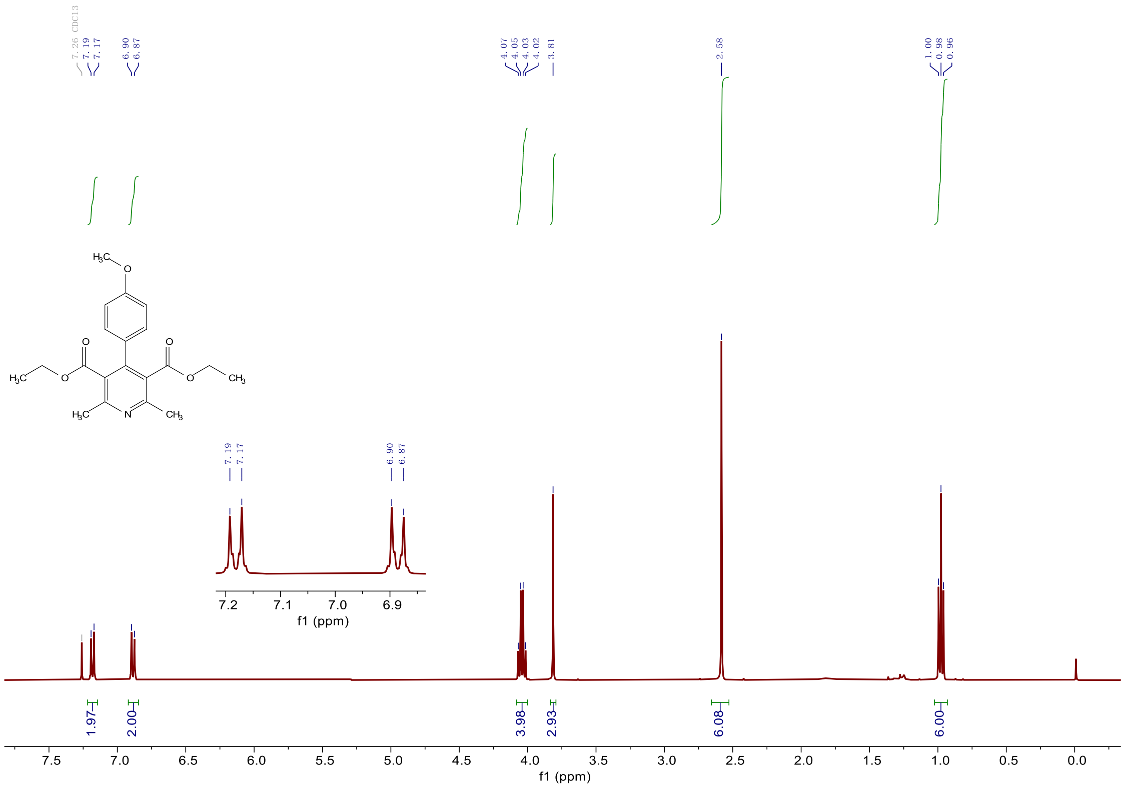
7.26 CDCl3
7.19
7.17
6.90
6.87
4.07
4.05
4.03
4.02
3.81
2.58
1.00
0.98
0.96
1.97
2.00
3.98
2.93
6.08
6.00
7.5
7.0
6.5
6.0
5.5
5.0
4.5
4.0
3.5
3.0
2.5
2.0
1.5
1.0
0.5
0.0
f1 (ppm)
7.2
7.1
7.0
6.9

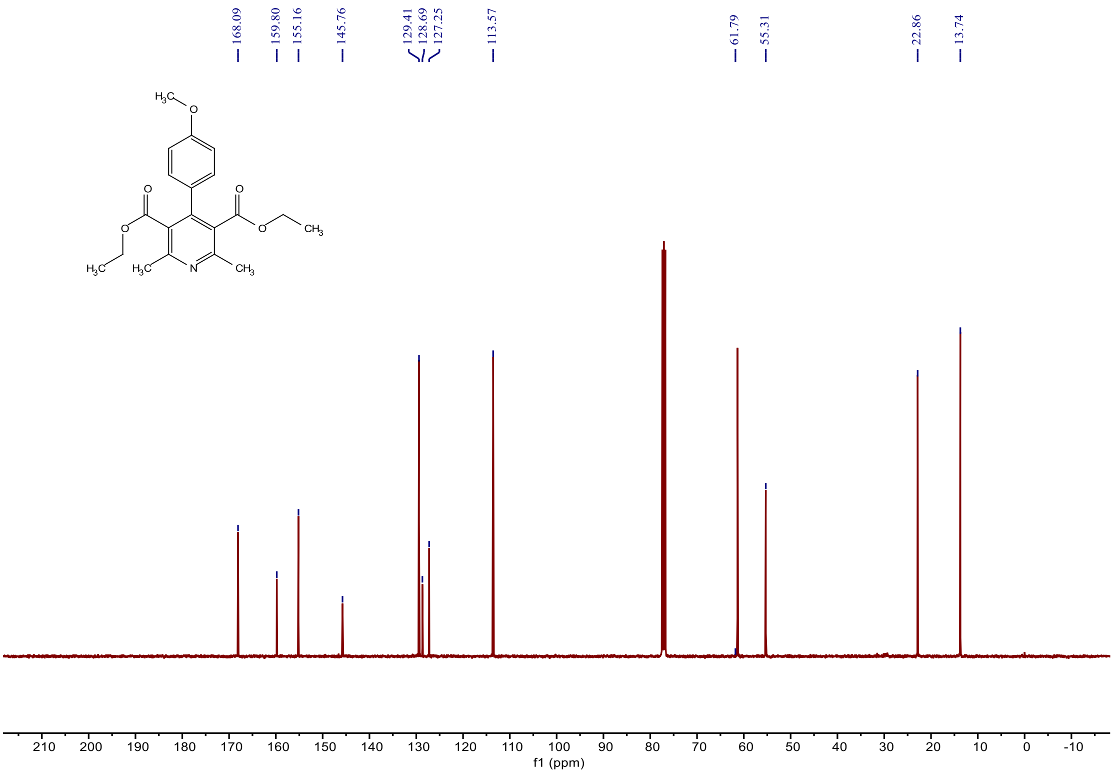
168.09
159.80
155.16
145.76
129.41
128.69
127.25
113.57
61.79
55.31
22.86
13.74
210
200
190
180
170
160
150
140
130
120
110
100
90
80
70
60
50
40
30
20
10
0
-10
f1 (ppm)

Compound **2l**

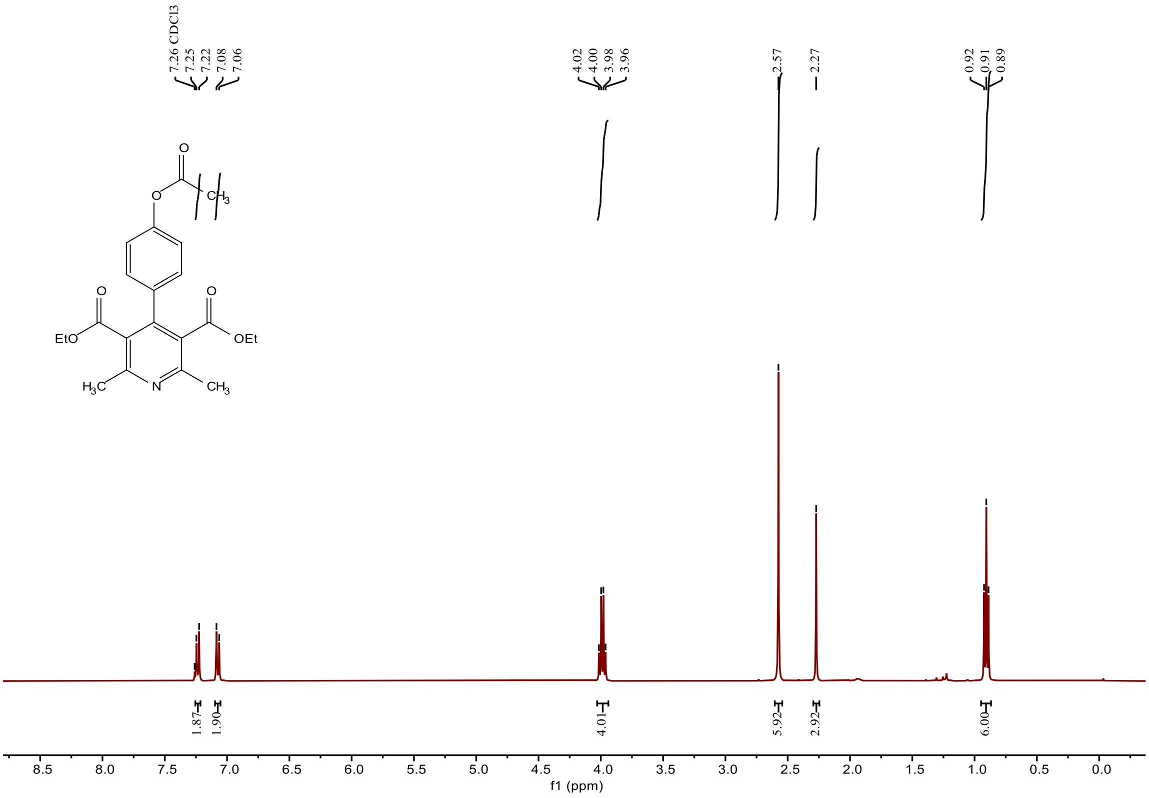
7.26 CDCl3
7.25
7.22
7.08
7.06
4.02
4.00
3.98
3.96
2.57
2.27
0.92
0.91
0.89
O
O
CH3
O
O
EtO
OEt
H3C
N
CH3
1.87
1.90
4.01
5.92
2.92
6.00
8.5 8.0 7.5 7.0 6.5 6.0 5.5 5.0 4.5 4.0 3.5 3.0 2.5 2.0 1.5 1.0 0.5 0.0
f1 (ppm)

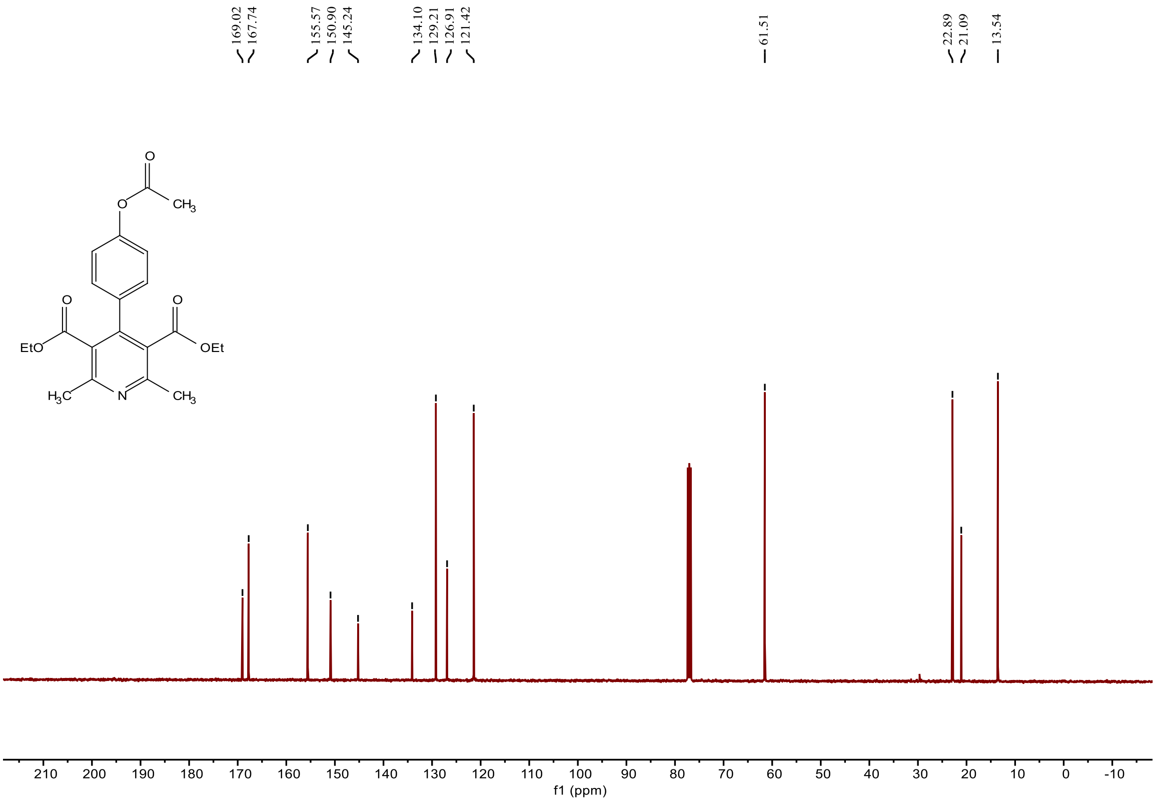
169.02
167.74
155.57
150.90
145.24
134.10
129.21
126.91
121.42
61.51
22.89
21.09
13.54
O
O
CH3
O
O
EtO
OEt
H3C
N
CH3
210 200 190 180 170 160 150 140 130 120 110 100 90 80 70 60 50 40 30 20 10 0 -10
f1 (ppm)

Compound **2m**

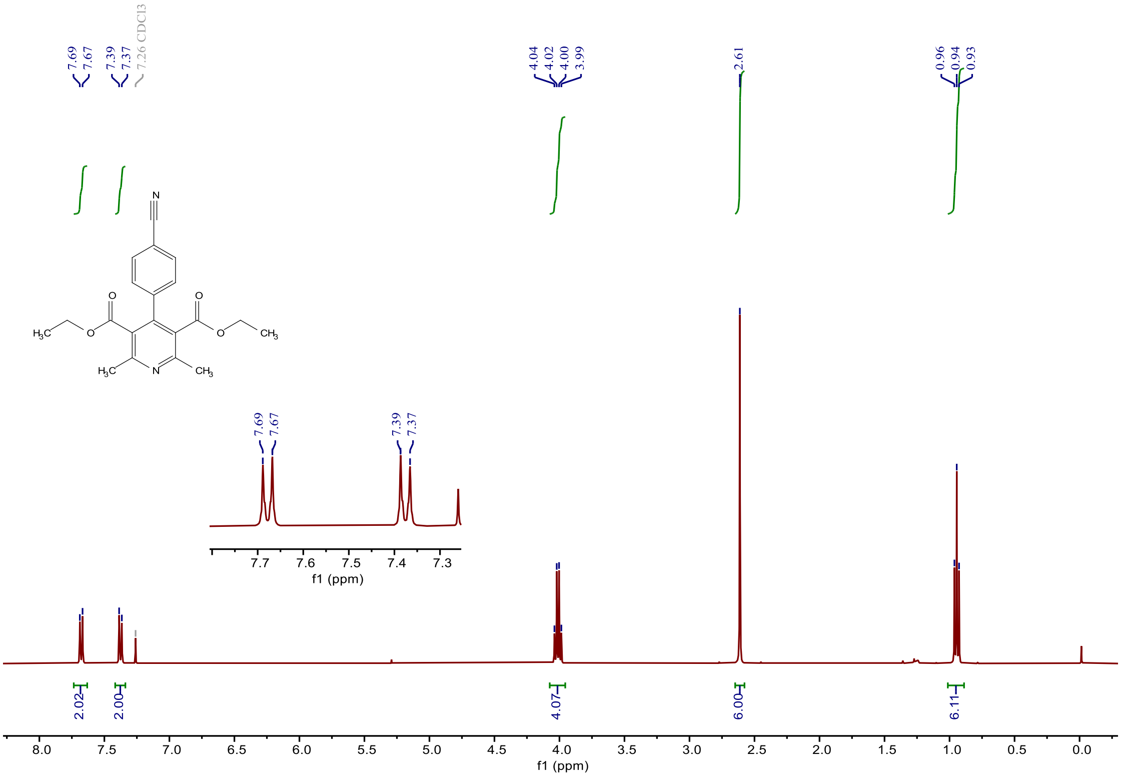
7.69
7.67
7.39
7.37
7.26 CDCl3
4.04
4.02
4.00
3.99
2.61
0.96
0.94
0.93
2.02
2.00
4.07
6.00
6.11
f1 (ppm)

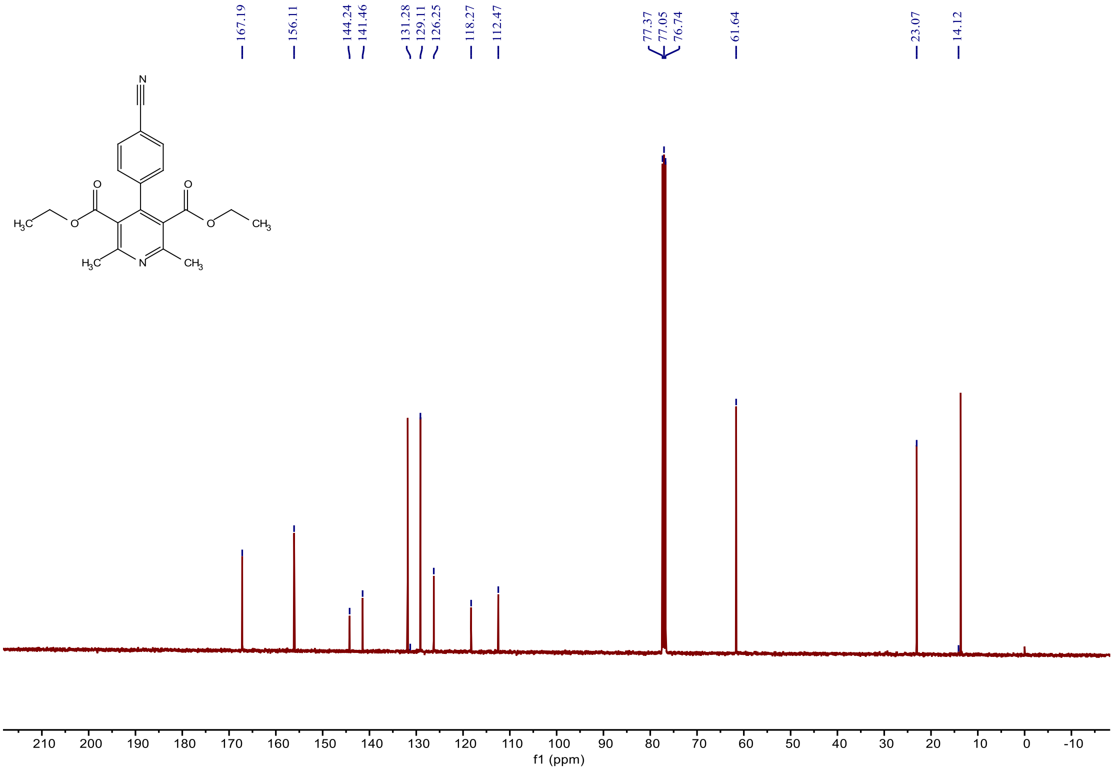
167.19
156.11
144.24
141.46
131.28
129.11
126.25
118.27
112.47
77.37
77.05
76.74
61.64
23.07
14.12
f1 (ppm)

Compound **2n**

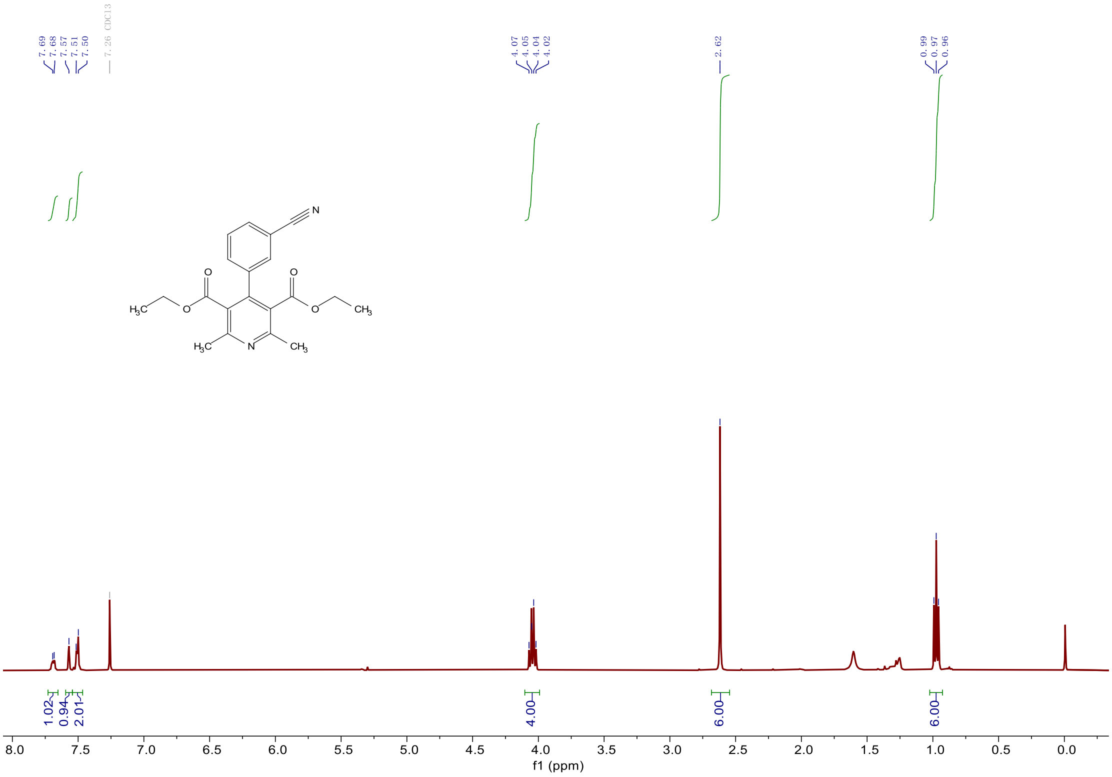
7.69
7.68
7.57
7.51
7.50
7.26 CDCl3
4.07
4.05
4.04
4.02
2.62
0.99
0.97
0.96
1.02
0.94
2.01
4.00
6.00
6.00
f1 (ppm)

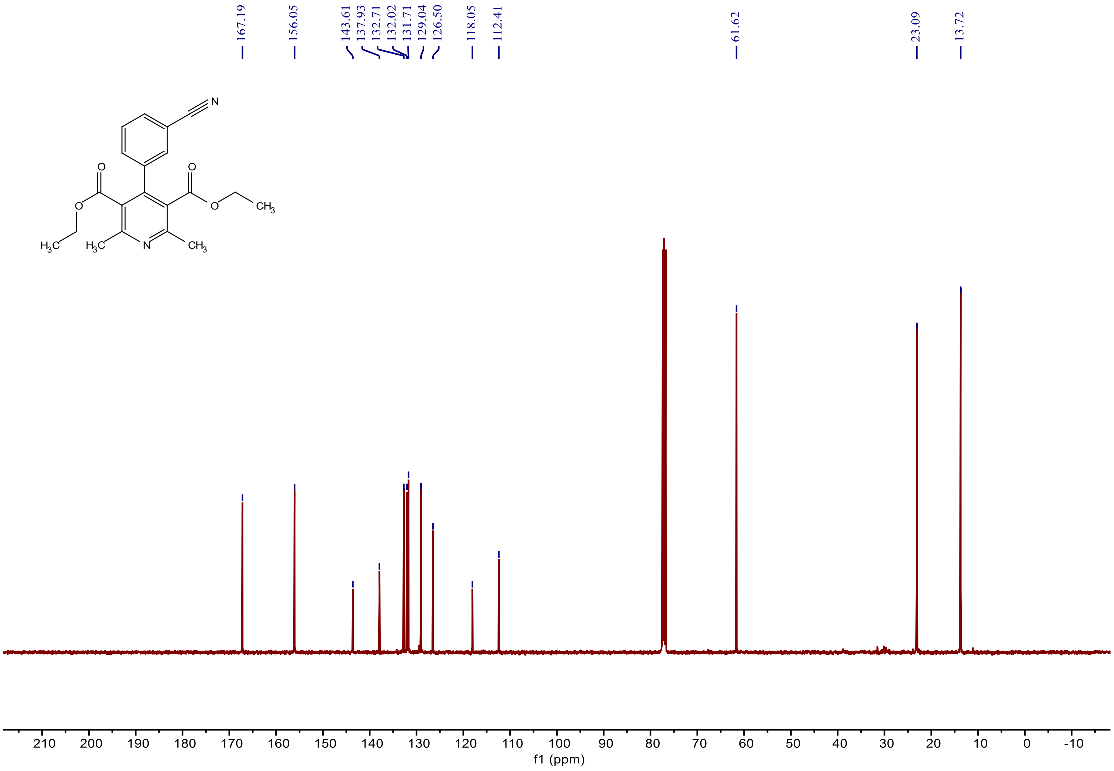
167.19
156.05
143.61
137.93
132.71
132.02
131.71
129.04
126.50
118.05
112.41
61.62
23.09
13.72
f1 (ppm)

Compound **2o**

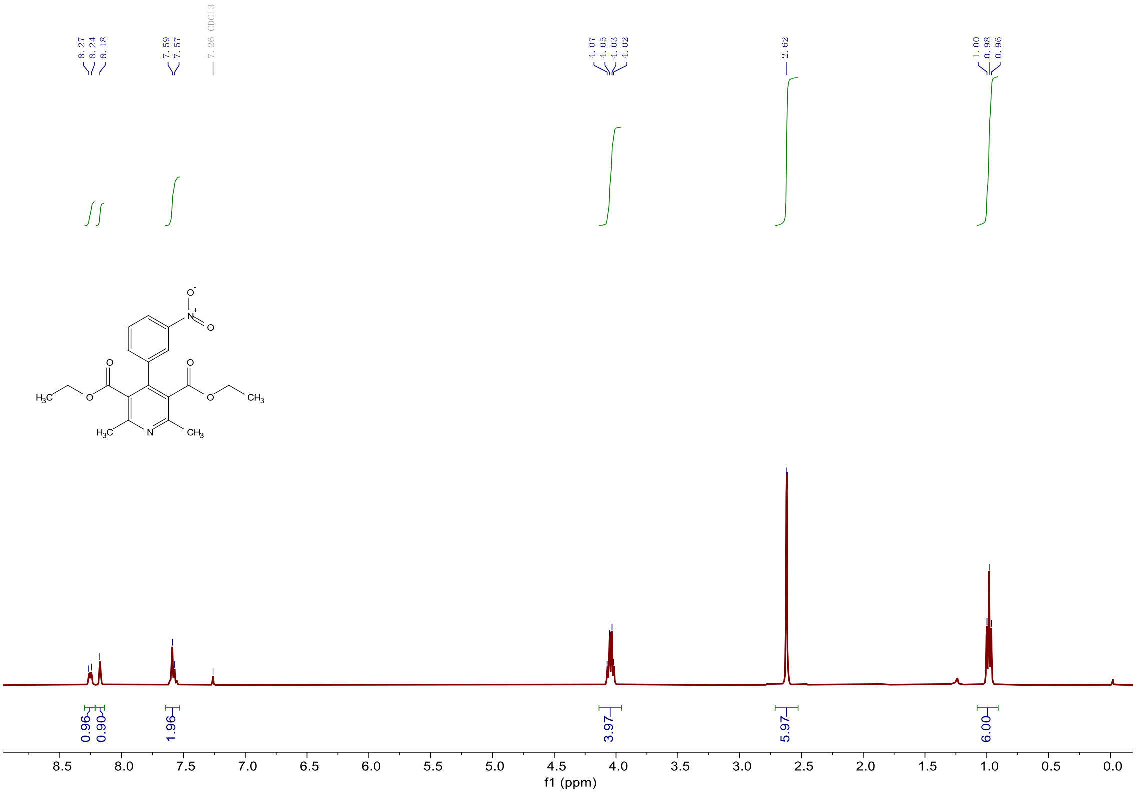
8.27
8.24
8.18
7.59
7.57
7.26 CDCl3
4.07
4.05
4.03
4.02
2.62
1.00
0.98
0.96
0.96
0.90
1.96
3.97
5.97
6.00
f1 (ppm)

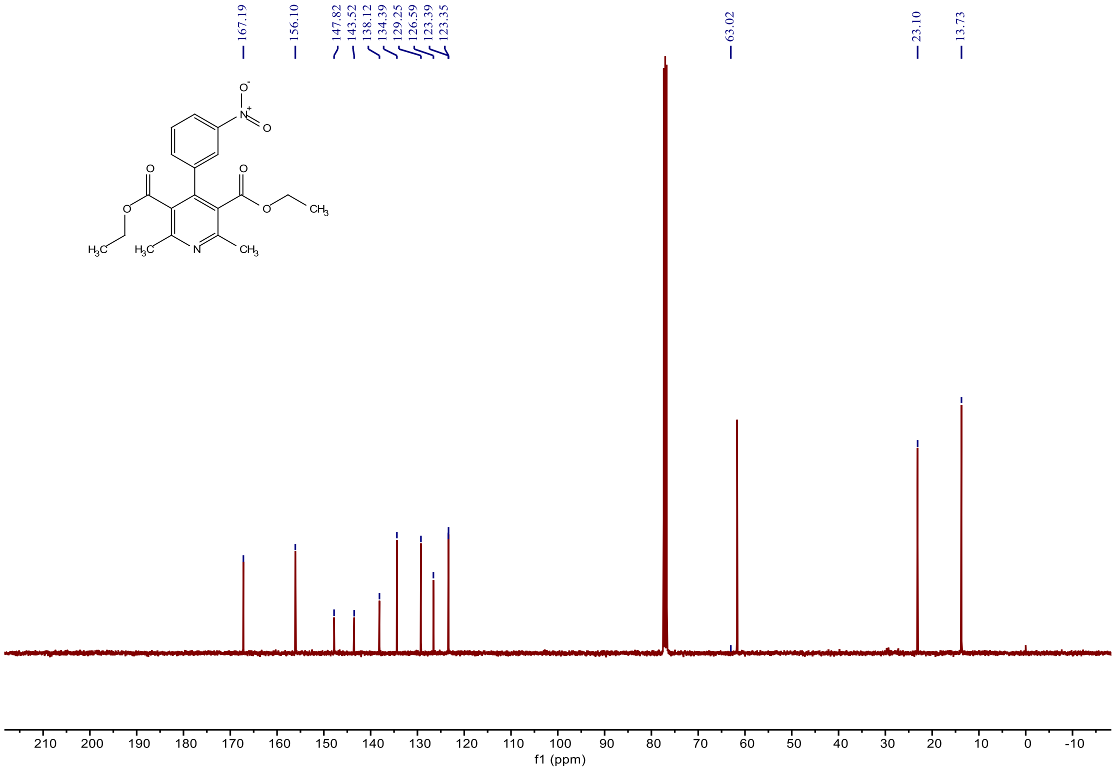
167.19
156.10
147.82
143.52
138.12
134.39
129.25
126.59
123.39
123.35
63.02
23.10
13.73
f1 (ppm)

Compound **2p**

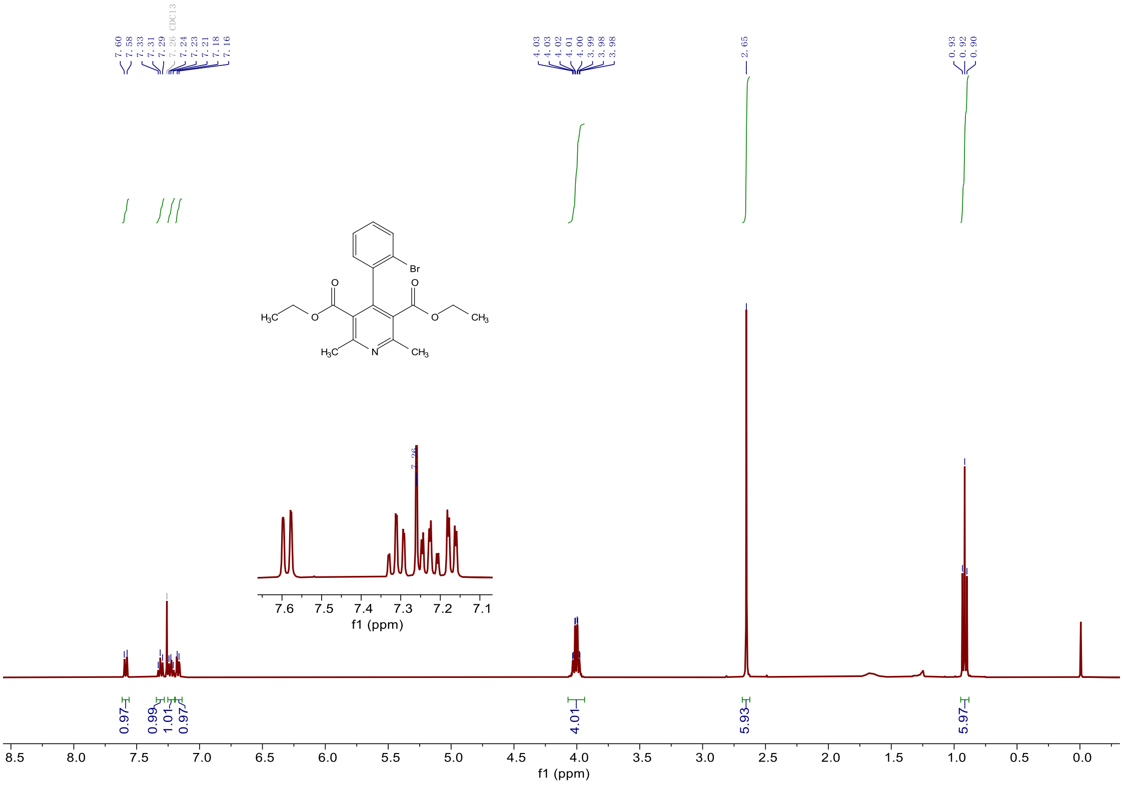

7.60
7.58
7.33
7.31
7.29
7.26 CDCl3
7.24
7.23
7.21
7.18
7.16
4.03
4.03
4.02
4.01
4.00
3.99
3.98
3.98
2.65
0.93
0.92
0.90
Br
O
O
O
O
H3C
CH3
H3C
N
CH3
7.6
7.5
7.4
7.3
7.2
7.1
f1 (ppm)
0.97
0.99
1.01
0.97
4.01
5.93
5.97
8.5
8.0
7.5
7.0
6.5
6.0
5.5
5.0
4.5
4.0
3.5
3.0
2.5
2.0
1.5
1.0
0.5
0.0
f1 (ppm)

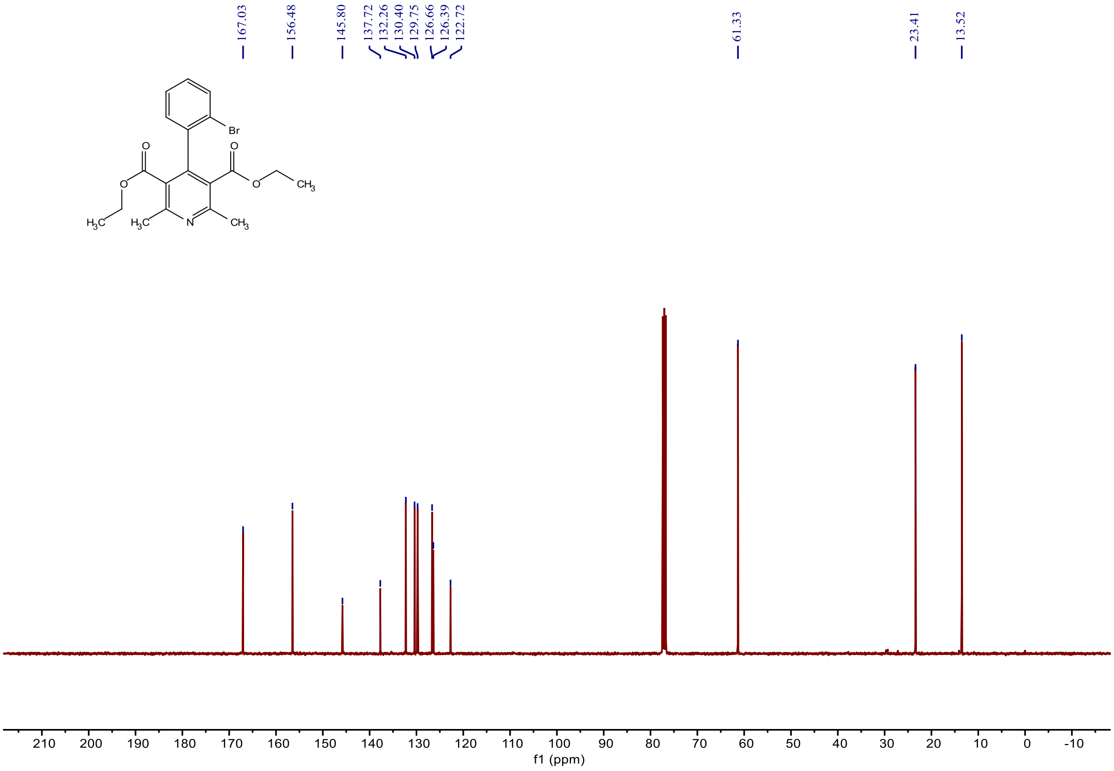

167.03
156.48
145.80
137.72
132.26
130.40
129.75
126.66
126.39
122.72
61.33
23.41
13.52
Br
O
O
O
O
CH3
H3C
H3C
N
CH3
210
200
190
180
170
160
150
140
130
120
110
100
90
80
70
60
50
40
30
20
10
0
-10
f1 (ppm)

Compound **2q**

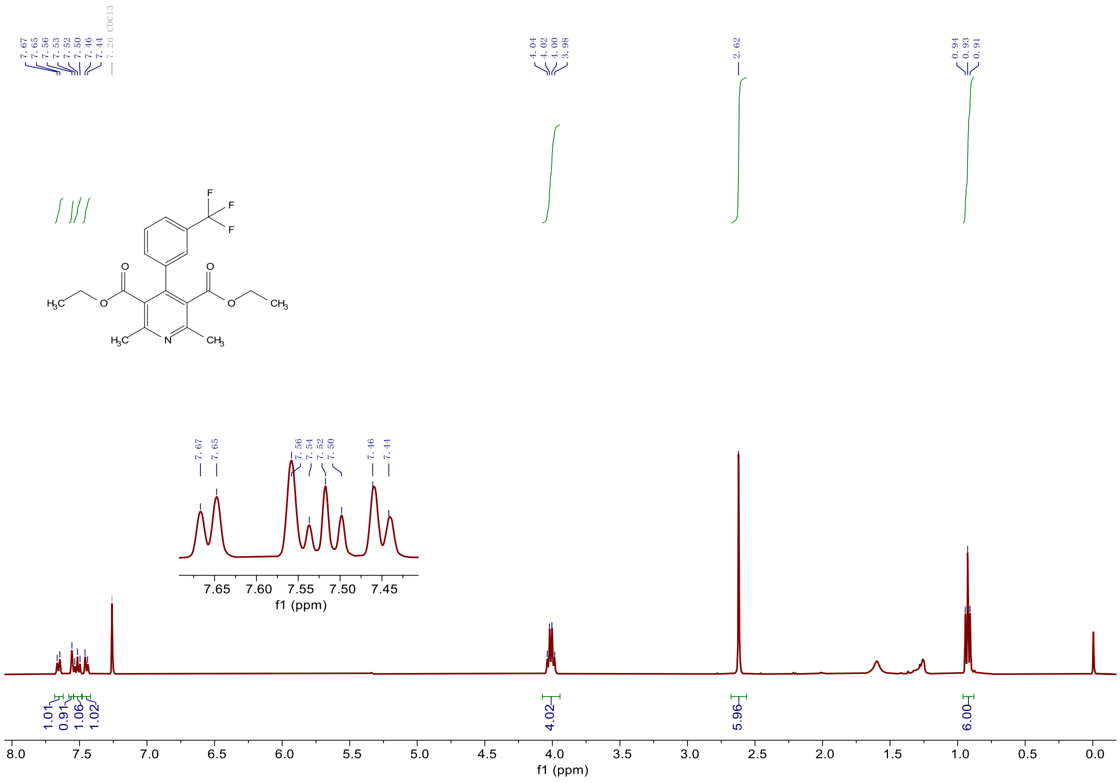

7.67
7.65
7.56
7.53
7.52
7.50
7.46
7.44
7.26 CDCl3
4.04
4.02
4.00
3.98
2.62
0.94
0.93
0.91
1.01
0.91
1.06
1.02
4.02
5.96
6.00
f1 (ppm)

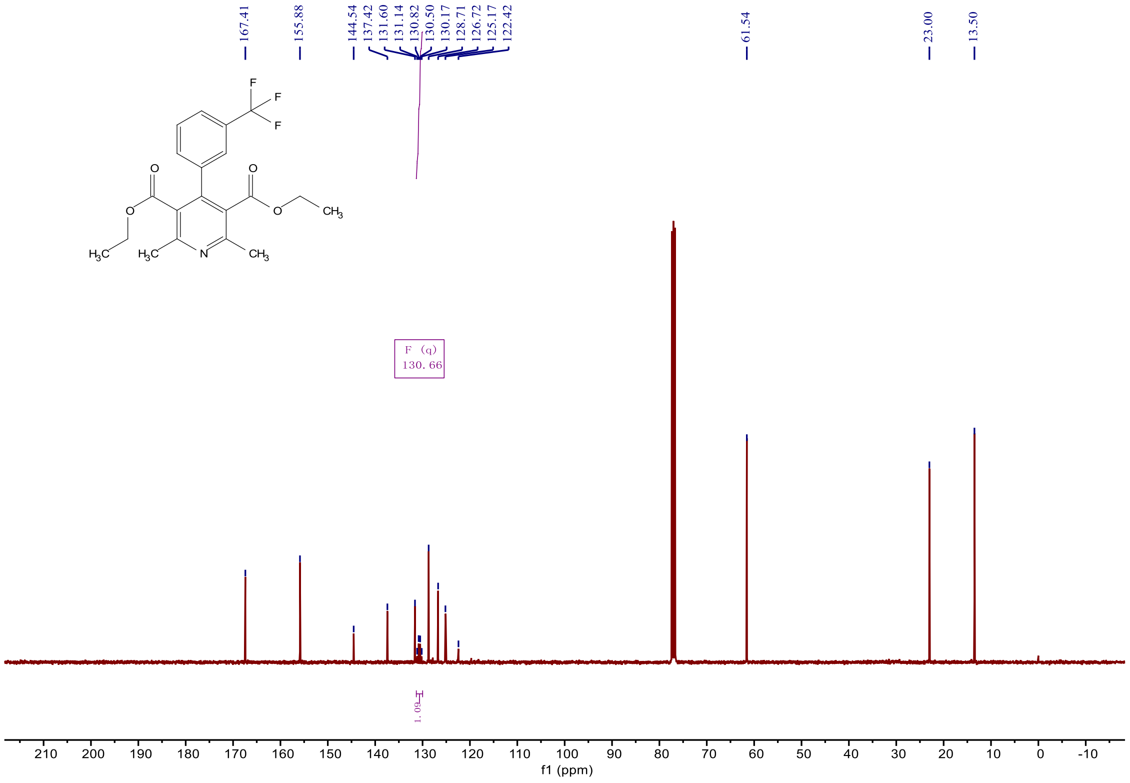

167.41
155.88
144.54
137.42
131.60
131.14
130.82
130.50
130.17
128.71
126.72
125.17
122.42
61.54
23.00
13.50
F (q)
130.66
f1 (ppm)

Compound **2r**

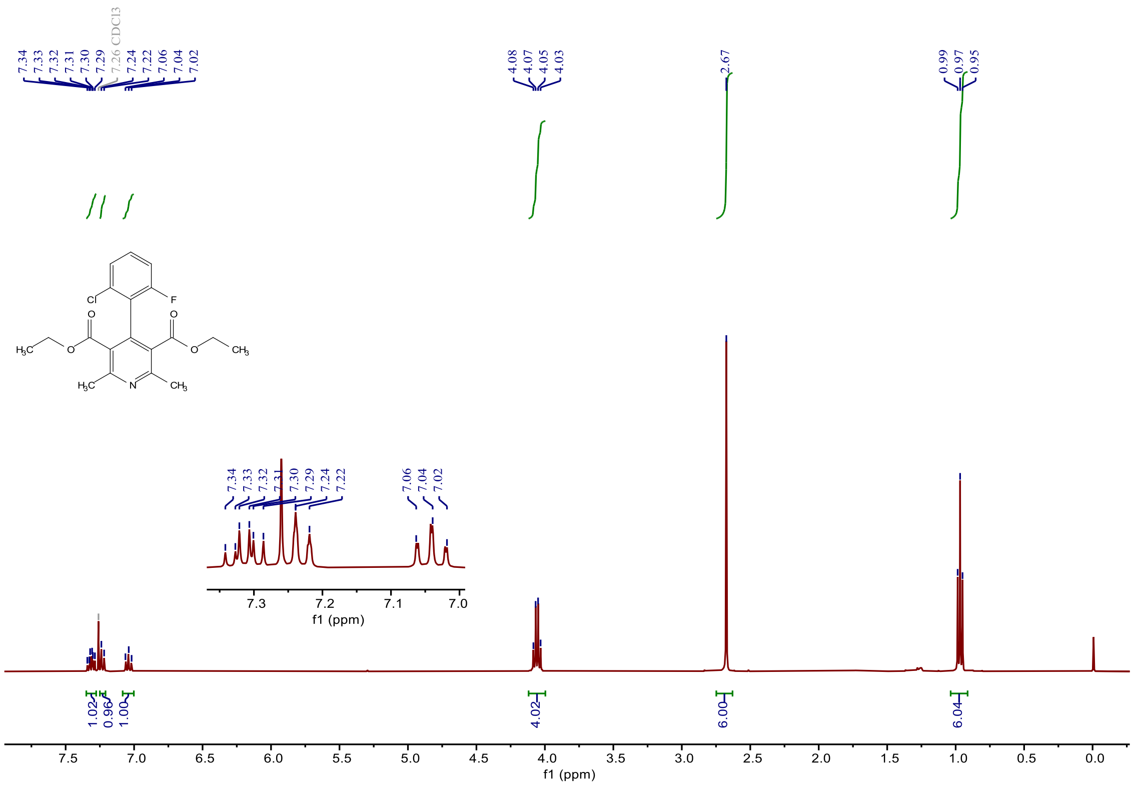
7.34
7.33
7.32
7.31
7.30
7.29
7.26 CDCl3
7.24
7.22
7.06
7.04
7.02
4.08
4.07
4.05
4.03
2.67
0.99
0.97
0.95
1.02
0.96
1.00
4.02
6.00
6.04
f1 (ppm)

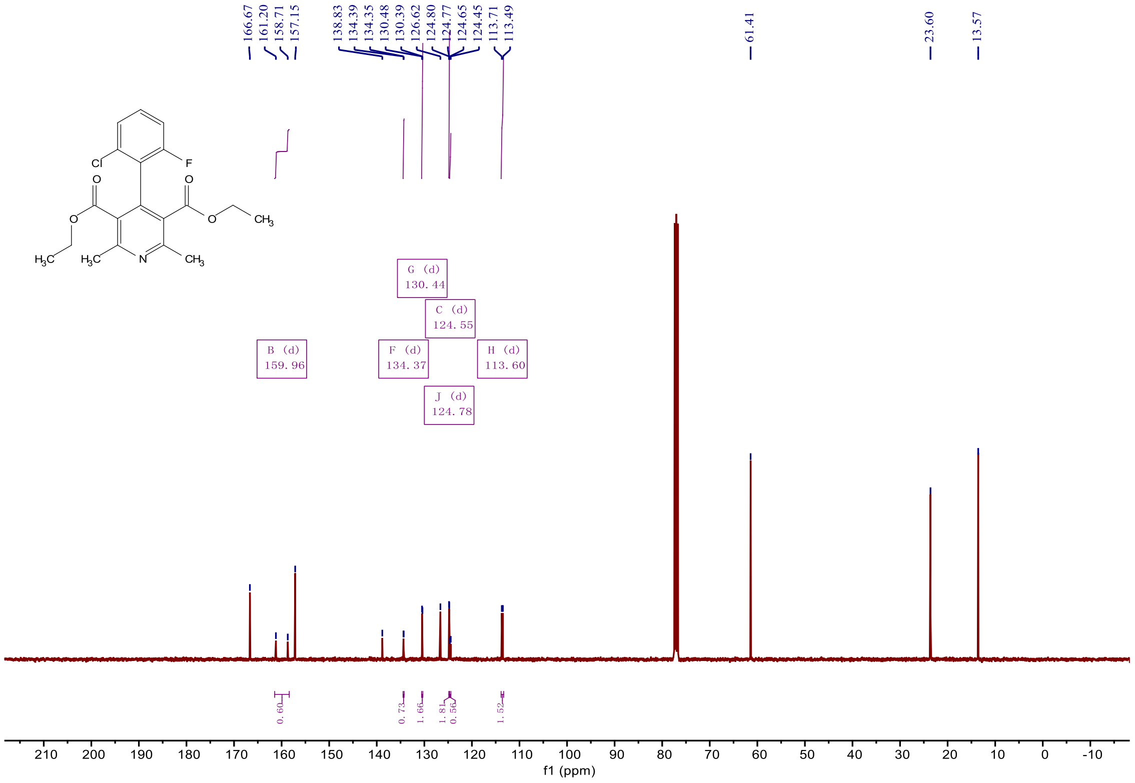
166.67
161.20
158.71
157.15
138.83
134.39
134.35
130.48
130.39
126.62
124.80
124.77
124.65
124.45
113.71
113.49
61.41
23.60
13.57
G (d) 130.44
C (d) 124.55
B (d) 159.96
F (d) 134.37
H (d) 113.60
J (d) 124.78
f1 (ppm)

Compound **2s**

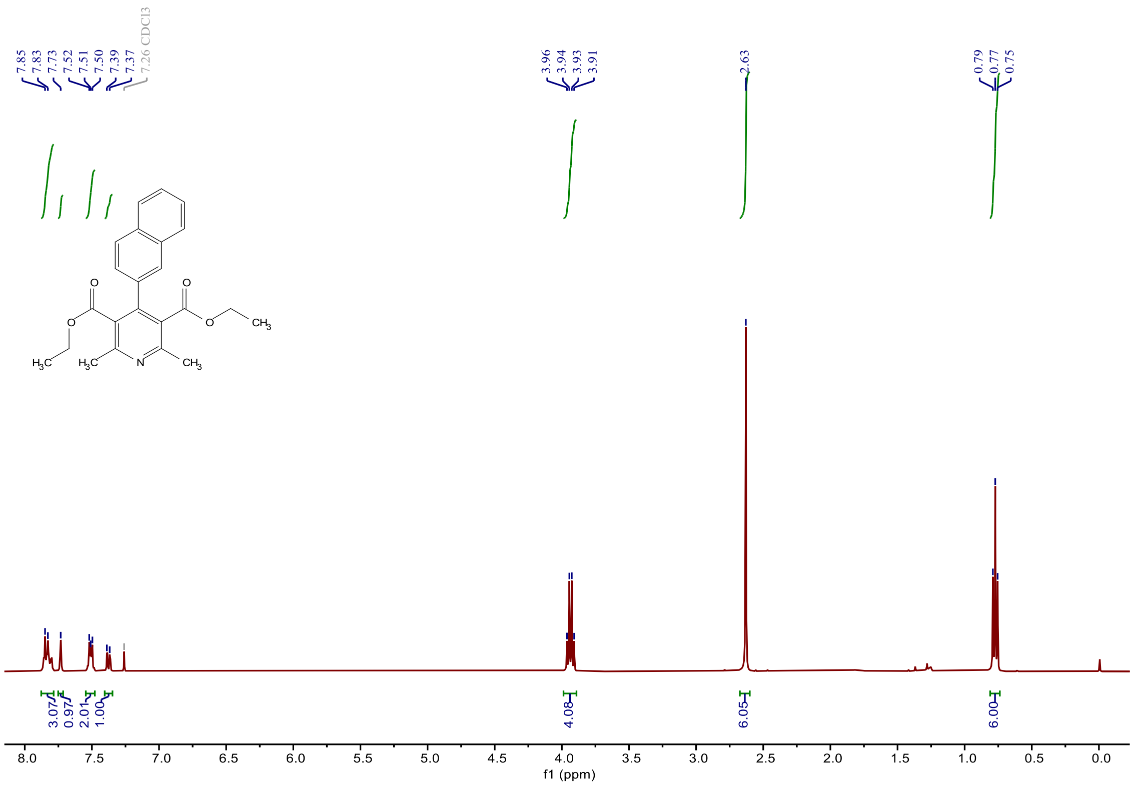
7.85
7.83
7.73
7.52
7.51
7.50
7.39
7.37
7.26 CDCl3
3.96
3.94
3.93
3.91
2.63
0.79
0.77
0.75
3.07
0.97
2.01
1.00
4.08
6.05
6.00
f1 (ppm)

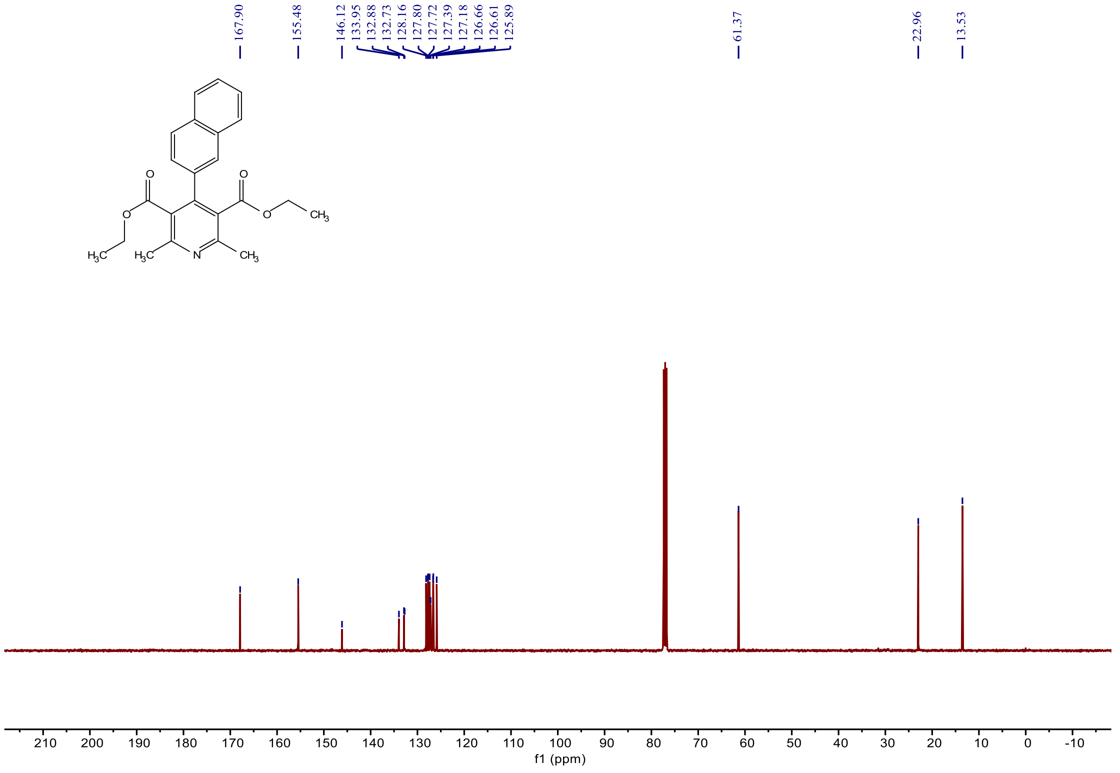
167.90
155.48
146.12
133.95
132.88
132.73
128.16
127.80
127.72
127.39
127.18
126.66
126.61
125.89
61.37
22.96
13.53
f1 (ppm)

Compound **2t**

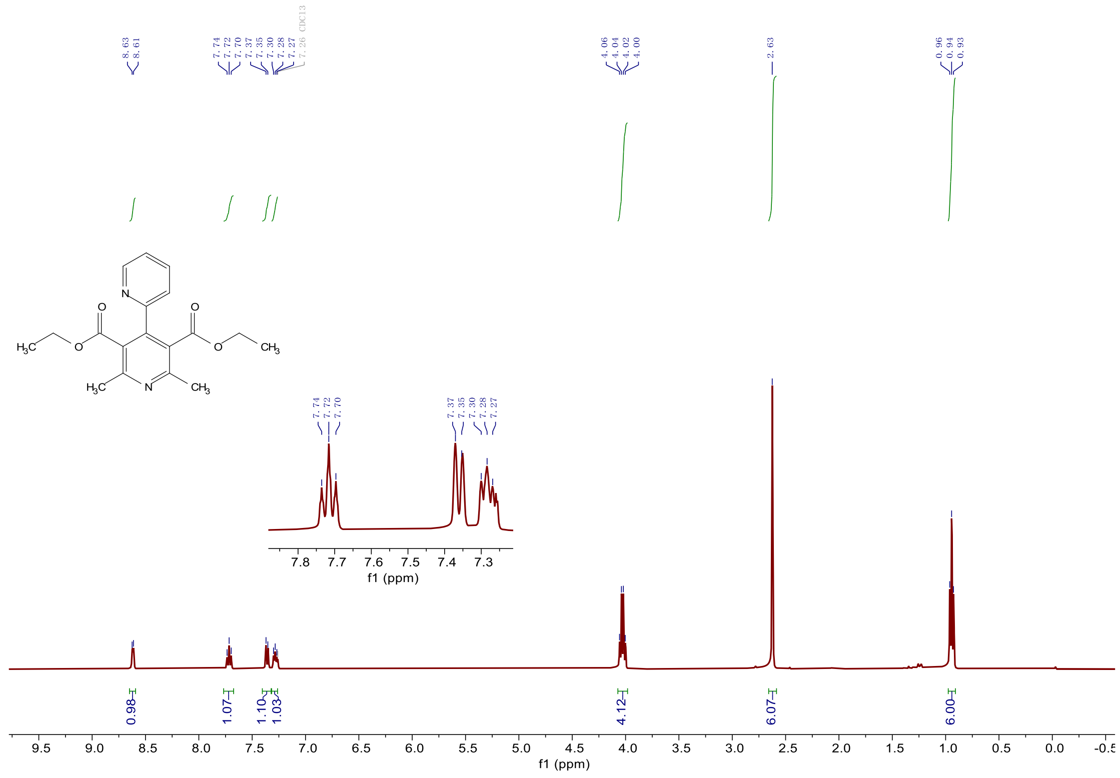

8.63
8.61
7.74
7.72
7.70
7.37
7.35
7.30
7.28
7.27
7.26 CDCl3
4.06
4.04
4.02
4.00
2.63
0.96
0.94
0.93
0.98
1.07
1.10
1.03
4.12
6.07
6.00
f1 (ppm)

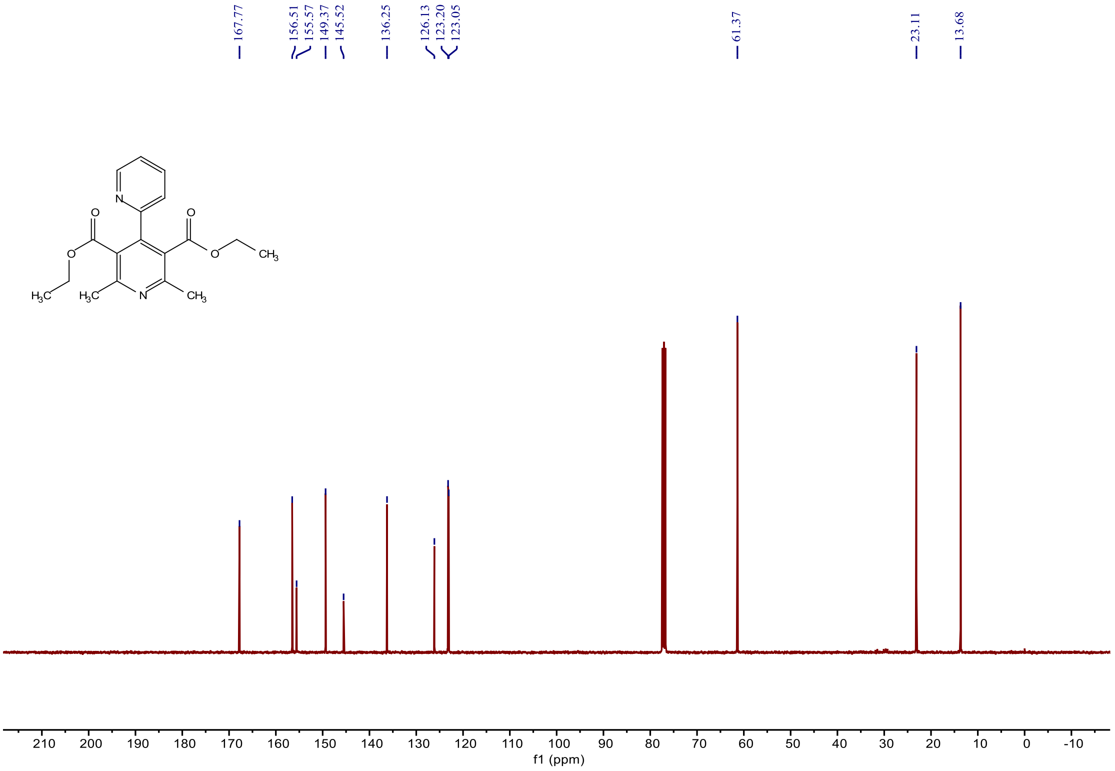

167.77
156.51
155.57
149.37
145.52
136.25
126.13
123.20
123.05
61.37
23.11
13.68
f1 (ppm)

Compound **2u**

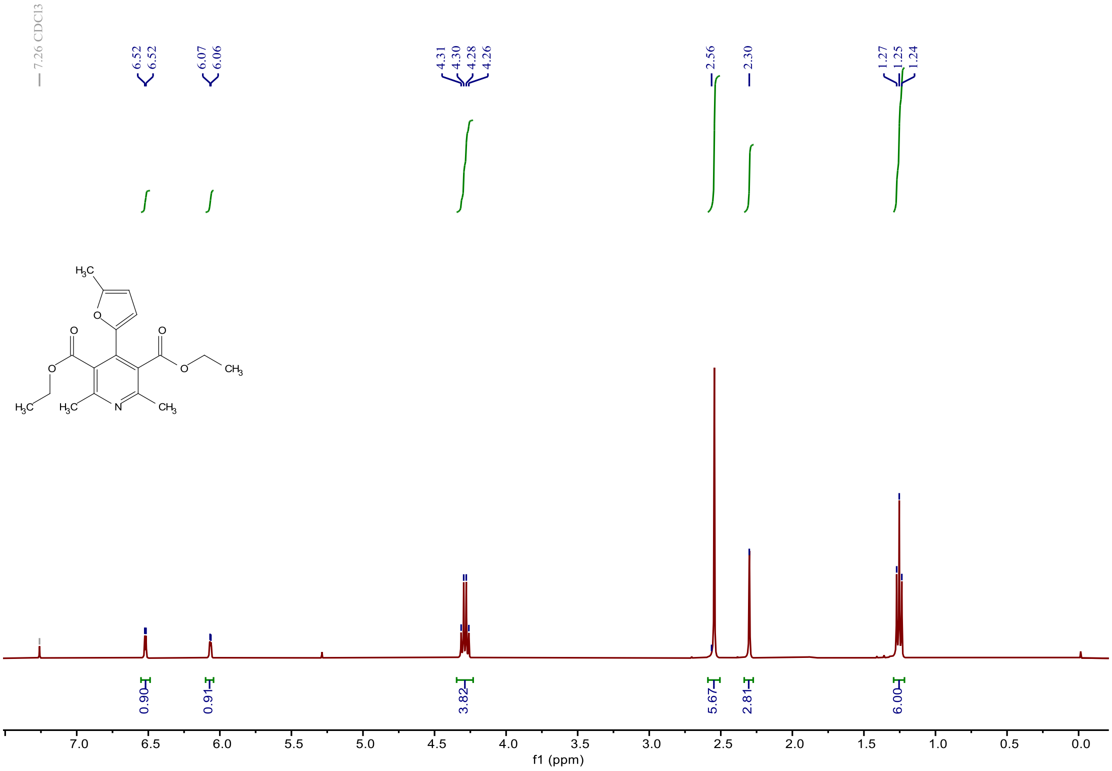

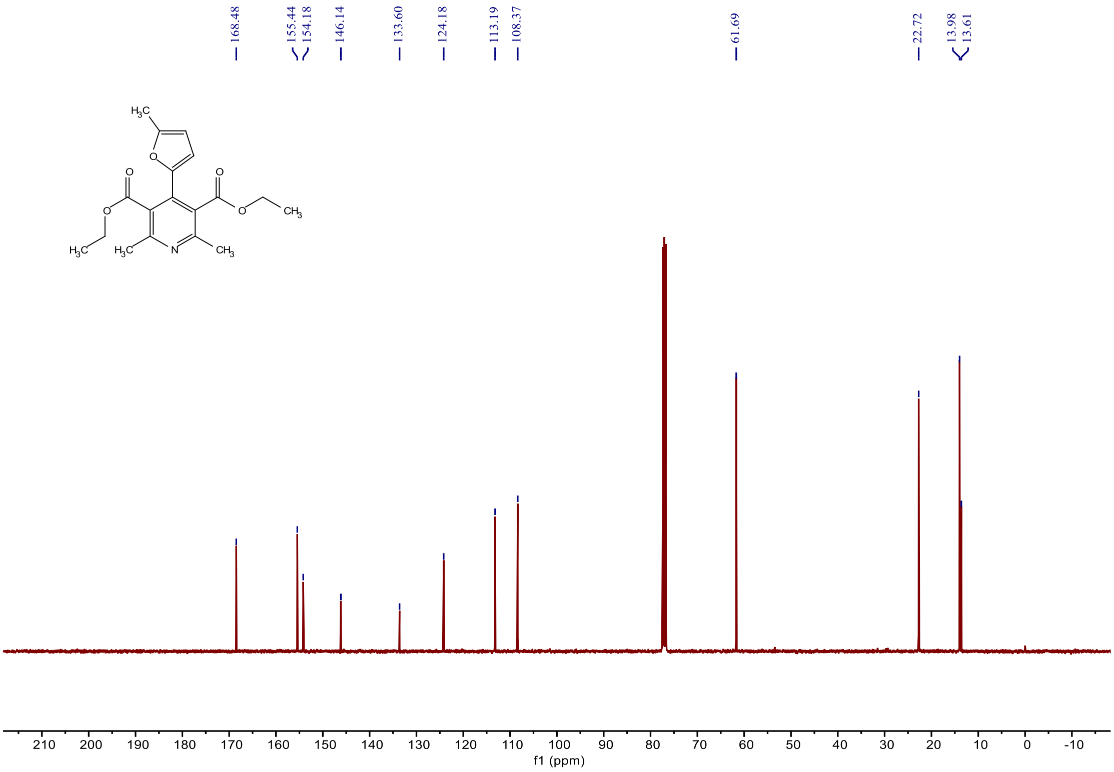

Compound **2v**

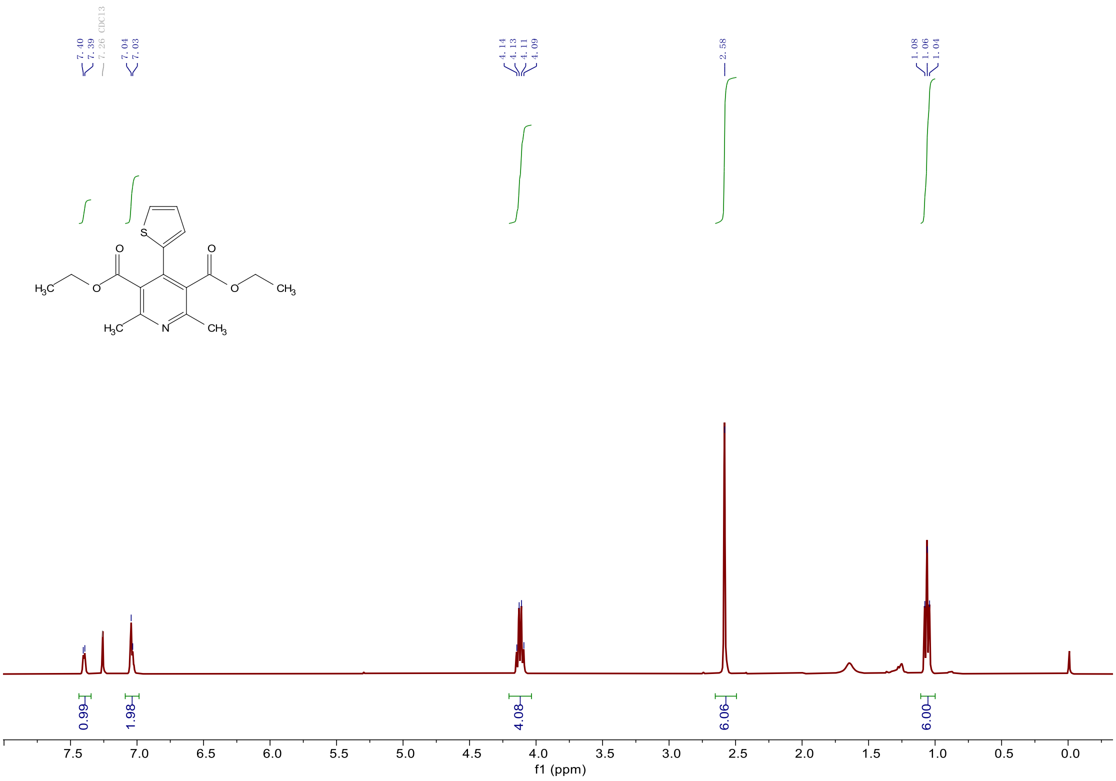

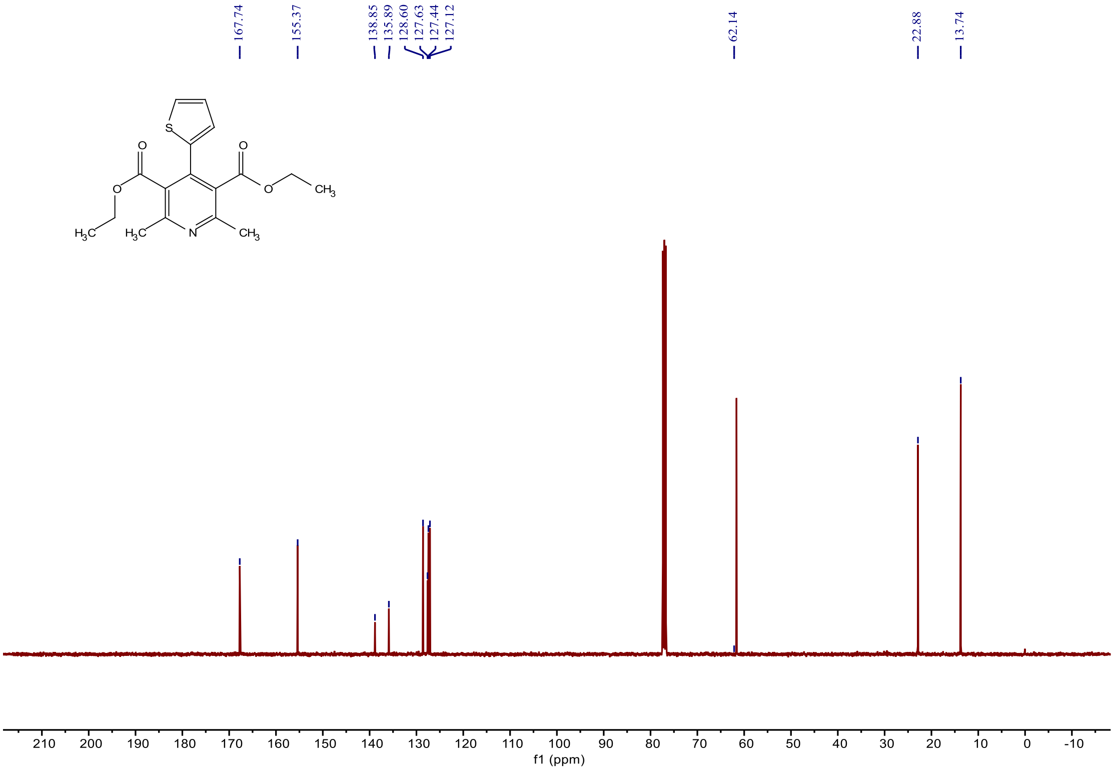

Compound **2w**

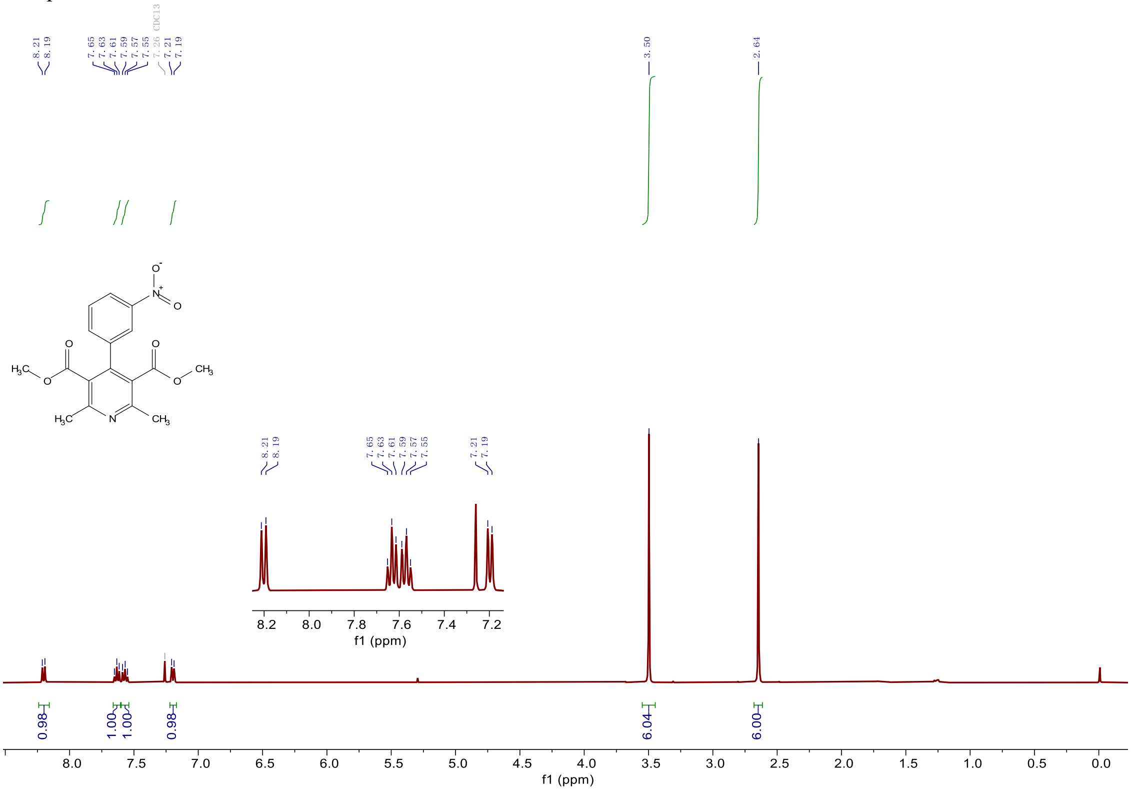
8.21
8.19
7.65
7.63
7.61
7.59
7.57
7.55
7.26 CDCl3
7.21
7.19
3.50
2.64
0.98
1.00
1.00
0.98
6.04
6.00
f1 (ppm)

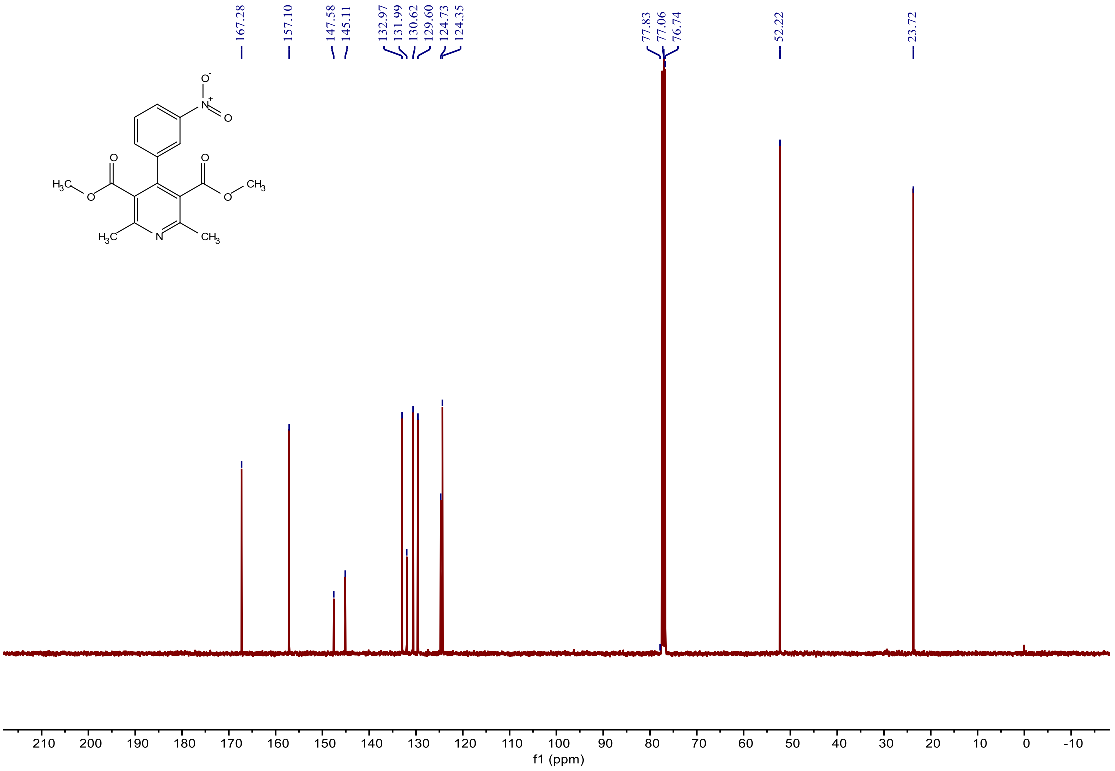
167.28
157.10
147.58
145.11
132.97
131.99
130.62
129.60
124.73
124.35
77.83
77.06
76.74
52.22
23.72
f1 (ppm)

Compound **2x**

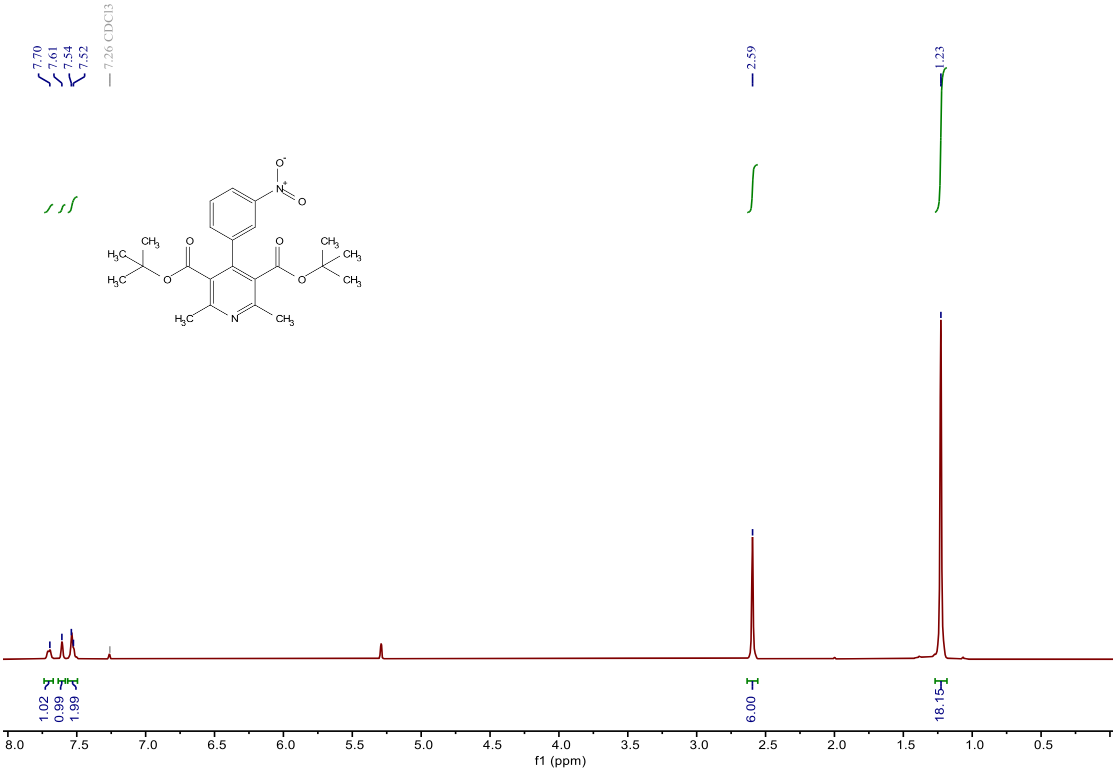

7.70
7.61
7.54
7.52
7.26 CDCl3
2.59
1.23
1.02
0.99
1.99
6.00
18.15
8.0 7.5 7.0 6.5 6.0 5.5 5.0 4.5 4.0 3.5 3.0 2.5 2.0 1.5 1.0 0.5
f1 (ppm)

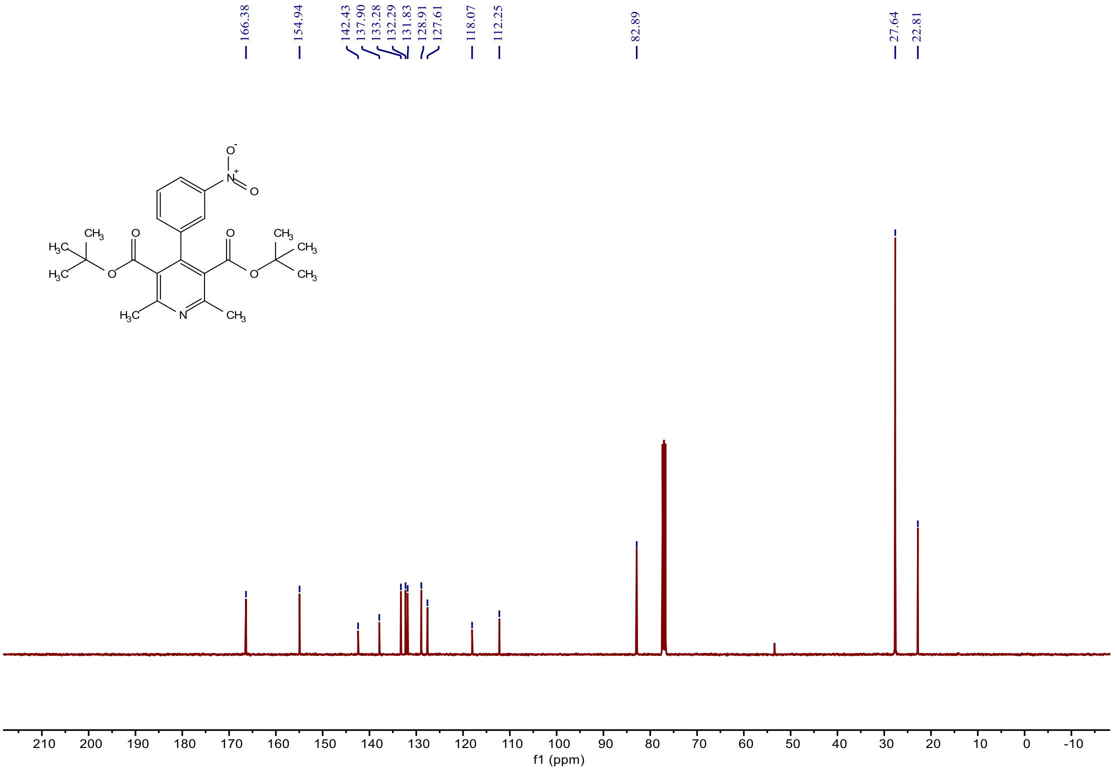

166.38
154.94
142.43
137.90
133.28
132.29
131.83
128.91
127.61
118.07
112.25
82.89
27.64
22.81
210 200 190 180 170 160 150 140 130 120 110 100 90 80 70 60 50 40 30 20 10 0 -10
f1 (ppm)

Compound **2y**

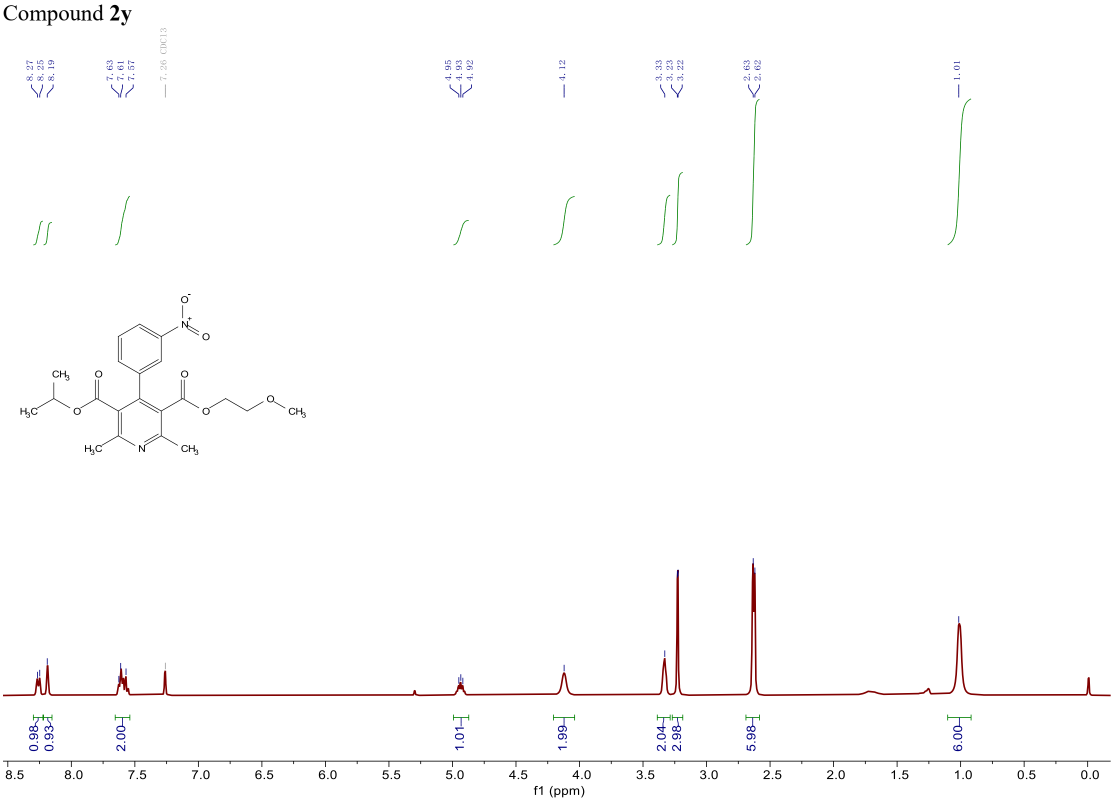

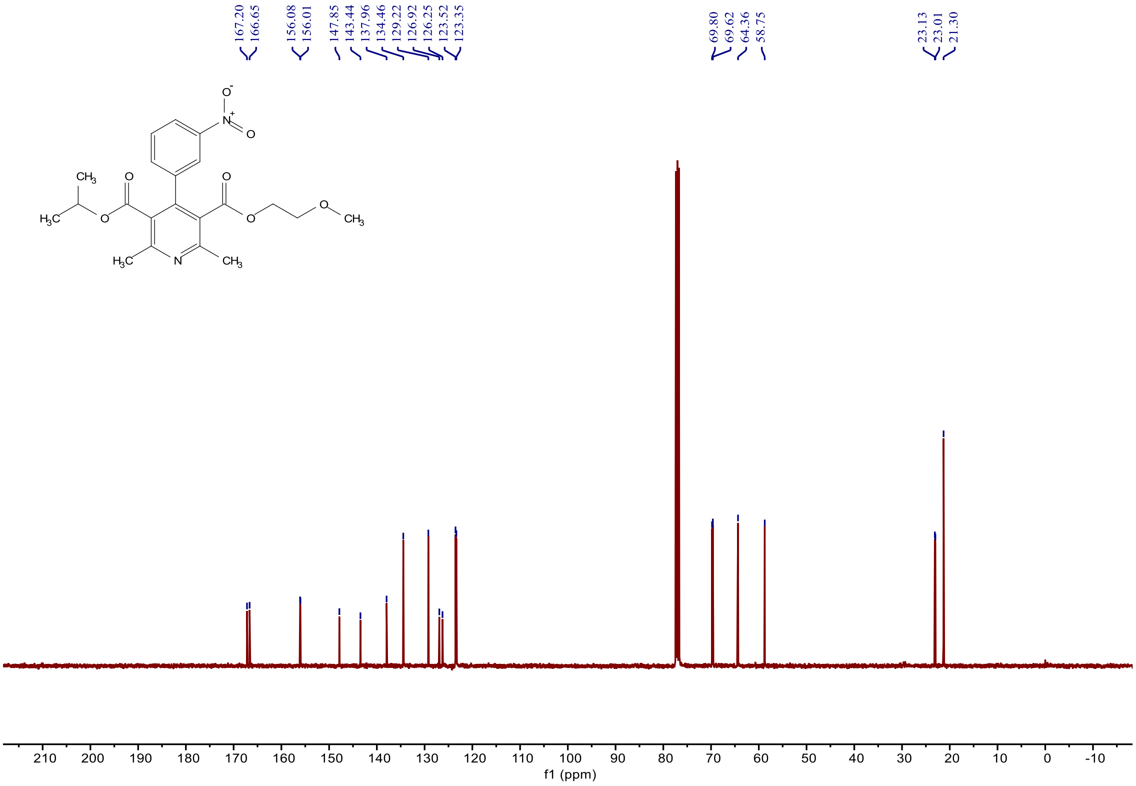

Compound **2z**

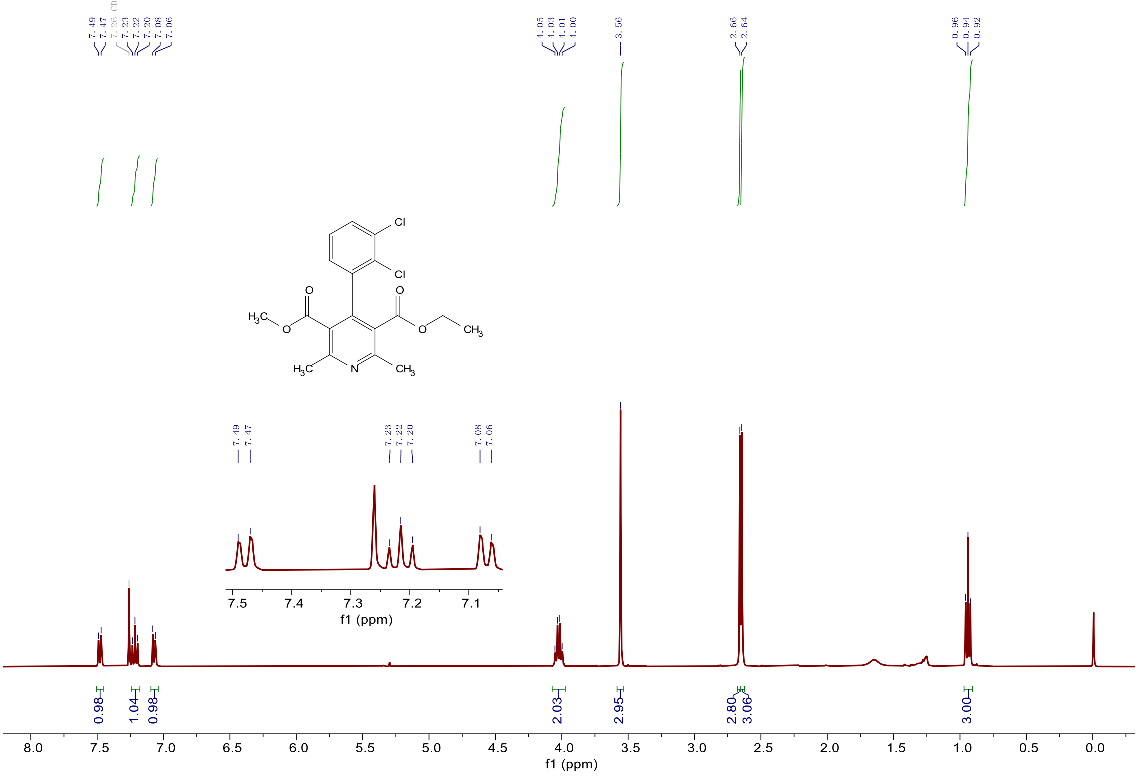

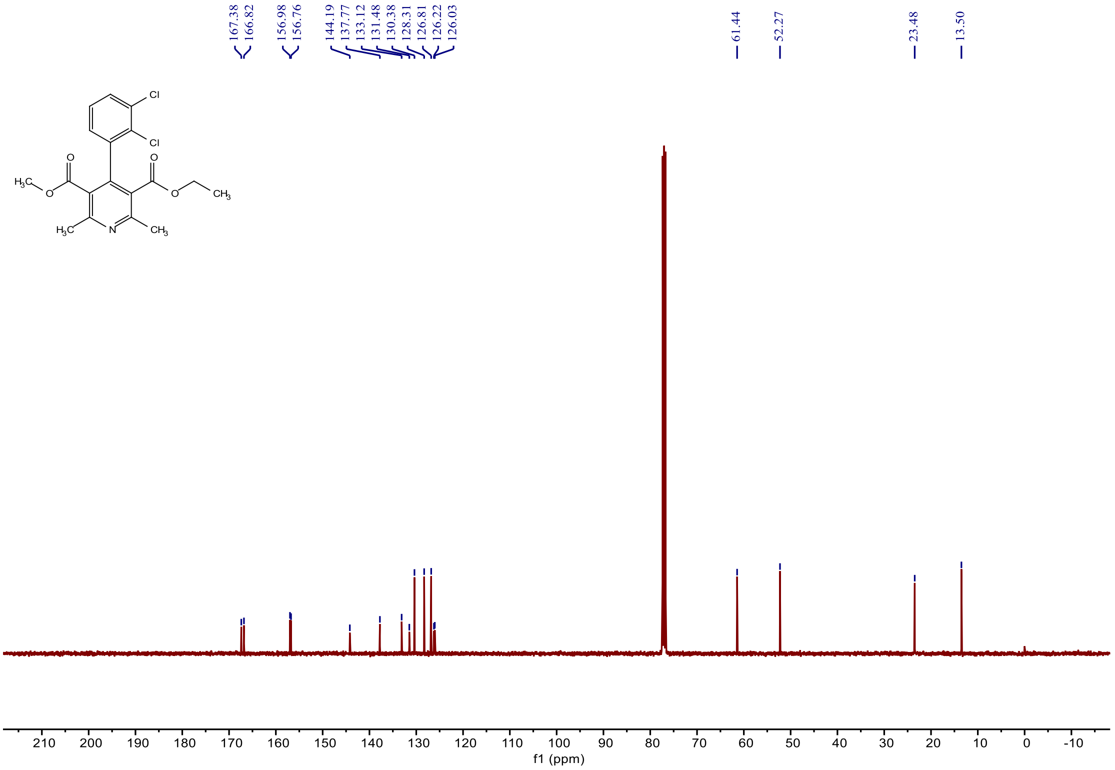

Compound **2aa**

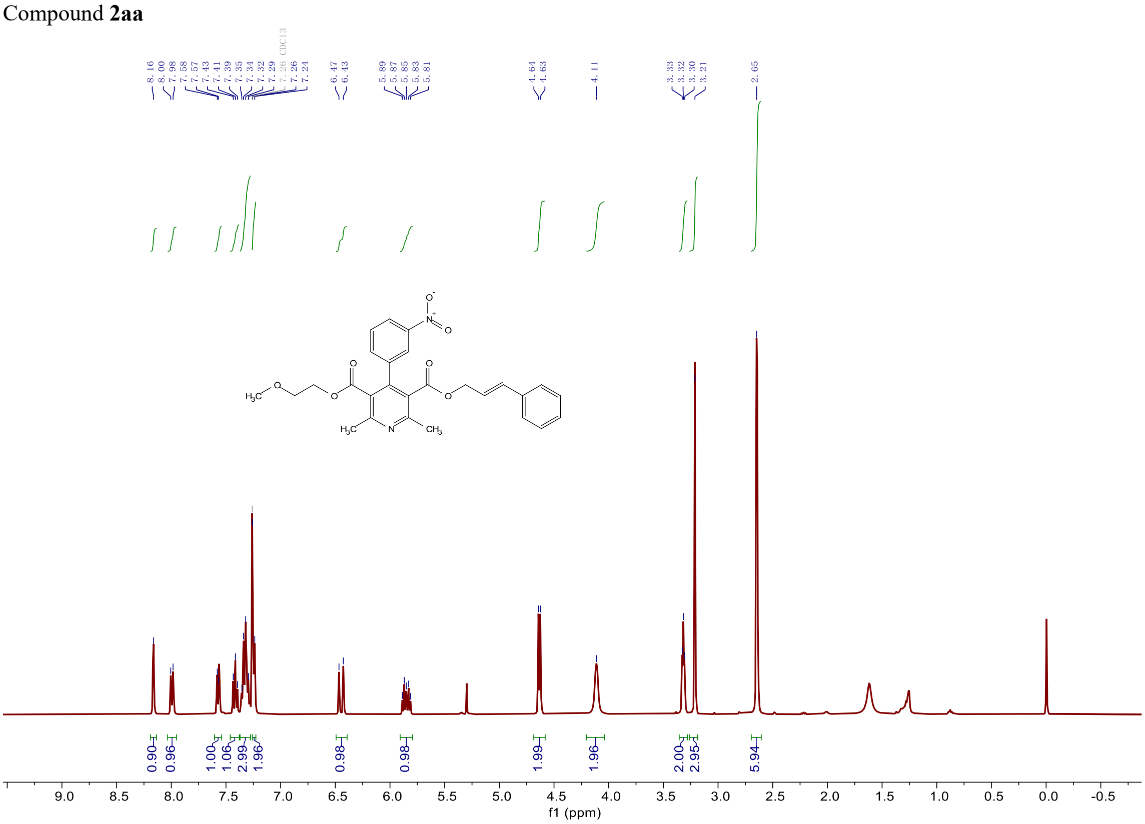

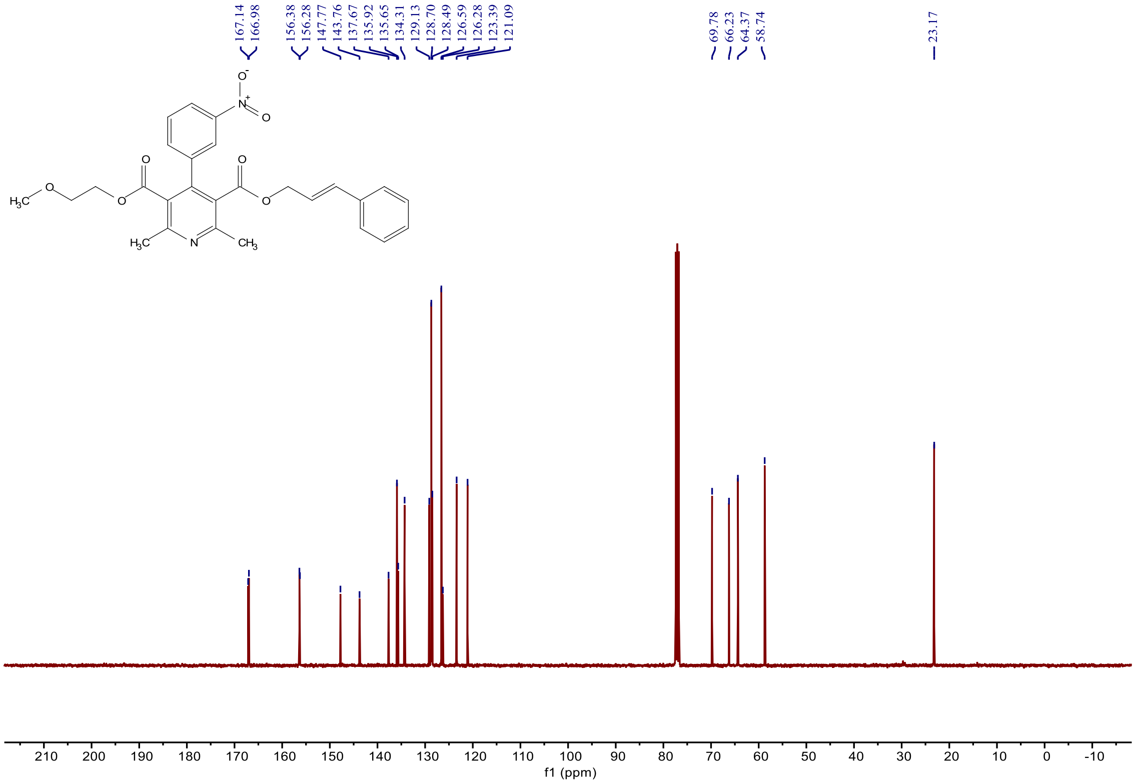

S6. References

[1] Hankovszky O H, Sár C P, Hideg K, et al. Synthesis of Spin-Labelled 1, 4-Dihydropyridines and Pyridines[J]. *Synthesis*, **1991**, 1991(01), 91-97.

[2] Vanden Eynde, J. J.; Delfosse, F.; Mayence, A.; Van Haverbeke, Y. Old Reagents, New Results: Aromatization of Hantzsch 1,4-Dihydropyridines with Manganese Dioxide and 2,3-dichloro-5,6-dicyano-1,4-benzoquinone. *Tetrahedron* **1995**, 51 (23), 6511-6516.

[3] Guangxun Li, Rong Chen, Lei Wu, Qingquan Fu, Xiaomei Zhang, Zhuo Tang, Alkyl Transfer from C-C Cleavage. *Angewandte Chemie International Edition* **2013**, 52, 8432-8436.

[4] Khaled, A., Stiti, M.Z., Habila, T. et al. Synthesis and Characterization of $La_{1-x}Sr_xMn_{1-y}Zn_yO_3$ Perovskites as an Efficient and Recoverable Catalyst for the Hantzsch Reaction. *Journal of Chemical Sciences* **2022**, 134, 86.

[5] Soltanmoradi, R.; Khodaei, M. M. Stabilization of Copper(II) Species on Highly Nitrogen‐Rich Modified UiO-66-NH_2: An Efficient Heterogeneous Catalyst for Synthesis of 1,4-Dihydropyridine via Hantzsch Reaction. *Journal of Organometallic Chemistry* **2025**, 1042, 123883.

[6] Baraldi, P. T.; Noël, T.; Wang, Q.; Hessel, V. The Accelerated Preparation of 1,4-Dihydropyridines Using Microflow Reactors. *Tetrahedron Letters* **2014**, 55 (13), 2090-2092.

[7] Sabitha, G., Arundhathi, K., Sudhakar, K., Sastry, B. S., & Yadav, J. S., $CeCl_3\cdot7H_2O$-Catalyzed One-Pot Synthesis of Hantzsch 1,4-Dihydropyridines at Room Temperature. *Synthetic Communications* **2009**, 39(16), 2843–2851.

[8] Elmira Hemmati, Somayeh Soleimani-Amiri, Mehran Kurdtabar; A CMC-g-poly(AA-co-AMPS)/Fe_3O_4 Hydrogel Nanocomposite as a Novel Biopolymer-based Catalyst in the Synthesis of 1,4-Dihydropyridines. *RSC Advances* **2023**, 13 (24), 16567–16583.

[9] Yadav J S, Reddy B V S, Basak A K, et al. Iodoxybenzoic Acid (IBX): An Efficient and Novel Oxidizing agent for the Aromatization of 1, 4-Dihydropyridines. *Synthesis* **2006** (03), 451-454.

[10] De Paolis O, Baffoe J, Landge S M, et al. Multicomponent Domino Cyclization-oxidative Aromatization on a Bifunctional Pd/C/K-10 Catalyst: An Environmentally benign Approach toward the Synthesis of Pyridines. *Synthesis*, **2008,** 21 3423-3428.

[11] Saad, M. H., El-Moselhy, T. F., S, El-Din, N., Mehany, A. B. M., Belal, A., Abourehab, M. A. S., El-Hamamsy, M. H., Discovery of New Symmetrical and Asymmetrical Nitrile-containing 1,4-Dihydropyridine Derivatives as Dual Kinases and P-Glycoprotein Inhibitors: Synthesis, in vitro Assays, and in Silico Studies. *Journal of Enzyme Inhibition and Medicinal Chemistry* **2022**, 37(1), 2489–2511.

[12] A. P. Vijayan, J. V. Oommen, P. M. Vijay, and K. Ramakrishnan, A Triazine Based Sulfonic Acid Anchored Porous Organic Polymer as an Efficient Heterogeneous Acid Catalyst for the Hantzsch Pyridine Synthesis, *Journal of Heterocyclic Chemistry* **2026**, 63(2), 237–246.

[13] Priede, E. and Zicmanis, A., One-Pot Three-Component Synthesis of Hantzsch 1,4-Dihydropyridines Promoted by Dimethyl Phosphate Ionic Liquids. *Helvetica* **2015**, 98, 1095-1103.

[14] Chang, C.-C.; Cao, S.; Kang, S.; Kai, L.; Tian, X.; Pandey, P.; Dunne, S. F.; Luan, C.-H.; Surmeier, D. J.; Silverman, R. B. Antagonism of 4-substituted 1,4-Dihydropyridine-3,5-dicarboxylates toward Voltage-Dependent L-type Ca^{2+} Channels Cav1.3 and Cav1.2. *Bioorganic & Medicinal Chemistry* **2010**, 18 (9), 3147-3158.

[15] Katy L. Bridgwood, Gemma E. Veitch, Steven V. Ley; Magnesium Nitride as a Convenient Source of Ammonia: Preparation of Dihydropyridines. *Organic Letters* **2008**, 10 (16), 3627–3629.

[16] Ravikumar Naik, T. R.; Shivashankar, S. A. Heterogeneous Bimetallic $ZnFe_2O_4$ Nanopowder Catalyzed Synthesis of Hantzsch 1,4-Dihydropyridines in Water. *Tetrahedron Letters* **2016**, 57 (36), 4046-4049.

[17] Castro, G.A.D., Leles, L.C.d.A., Varejão, E.V.V., Martins, F.T., Macruz, P.D., Pilau, E.J. and Fernandes, S.A., Green Synthesis of Hantzsch Dihydropyridines from Biorenewable Furans Derived from Lignocellulosic Biomass. *European Journal of Organic Chemistry* **2025**, 28, e202500278.

[18] Niaz, H.; Kashtoh, H.; Khan, J. A. J.; Khan, A.; Wahab, A.-t.; Alam, M. T.; Khan, K. M.; Perveen, S.; Choudhary, M. I. Synthesis of diethyl 4-substituted-2,6-dimethyl-1,4-dihydropyridine-3,5-dicarboxylates as a New Series of Inhibitors against Yeast α-glucosidase. *European Journal of Medicinal Chemistry* **2015**, 95, 199-209.

[19] Dos Santos, J. R. N.; Sciota, V. B. A.; Vargas, G. F.; Pinto, C. B.; Amarante, G. W.; Ellena, J.; Corrêa, A. G. Catalyst-Free Visible-Light Mediated α-alkylation of Quinazolines and Pyrrolo[2,1-f][1,2,4]triazine. *Tetrahedron* **2026**, 195, 135195.

[20] M. W. Roomi; Porphyria-Inducing Activity of a Series of Pyridine and Dihydropyridine Compounds. Investigation in a Cell Culture System. *Journal of Medicinal Chemistry* **1975**, 18 (5), 457–460.

[21] E. E. Ayling; 193. The Influences of Alkyl Groups in Carbonyl Compounds. *Journal of the Chemical Society* **1938**, 141, 1014–1023.

[22] Jing-Jiang Liu, Fu-Hu Guo, Fu-Jun Cui, Ji-Hua Zhu, Xiao-Yu Liu, Arif Ullah, Xi-Cun Wang, Zheng-Jun Quan; A Biomass-Derived N-doped Porous Carbon Catalyst for the Aerobic Dehydrogenation of Nitrogen Heterocycles. *New Journal of Chemistry* **2022**; 46 (4), 1791–1799.

[23] Shaikh, A. C.; Chen, C. Facile and Efficient Aromatization of 1,4-Dihydropyridines with $M(NO_3)_2 \cdot XH_2O$, TNCB, TBAP and HMTAI and Preparation of Deuterium Labeled Dehydronifedipine from Nifedipine-d_3. *Bioorganic & Medicinal Chemistry Letters* **2010**, 20 (12), 3664-3668.

[24] M. Janani, M. A. Senejani, T. M. Isfahani, Superparamagnetic Core-shell Metal–organic Framework Fe_3O_4@Ni-MOF as Efficient Catalyst for Oxidation of 1,4-Dihydropyridines Using Hydrogen Peroxide, *Applied Organometallic Chemistry* **2021**, 35(10), e6350.

[25] Hosseininasab, F.S., Memarian, H.R. & Asgari, M. Effect of the Heterocyclic Ring-Fusion on the Photochemical Reactivity of 1,4-Dihydropyridines, Polyhydroquinolines, and Decahydroacridine-1,8-Diones. *Research on Chemical Intermediates* **2025**, 51, 2705–2754.

[26] Su, J., Zhang, C., Lin, D., Duan, Y., Fu, X., & Mu, R.. Oxidative Aromatization of Hantzsch 1,4-Dihydropyridines by H_2O_2/V_2O_5 at Room Temperature. *Synthetic Communications* **2010**, 40(4), 595–600.

[27] Azad Bani, Abdollah Salimi, Farzad Nikpour and Amin Rostami, Electrocatalytic Dehydrogenation of 1,4-Dihydropyridines and 2,3-Dihydroquinazolinones Using 4-Phenylurazole as a Novel Redox Mediator Under Mild Conditions. *Journal of The Electrochemical Society* **2025**, 172, 065502.

[28] Shahabi, D., Amrollahi, M.A. & Jafari, A.A. NaI Readily Mediated Oxidative Aromatization of Hantzsch 1,4-Dihydropyridines with Hydrogen Peroxide at Room Temperature: A Green Procedure. *Journal of the Iranian Chemical Society* **2011**, 8, 1052–1057.

[29] Yadav J S, Reddy B V S, Basak A K, et al. Iodoxybenzoic Acid (IBX): An Efficient and Novel Oxidizing agent for the Aromatization of 1, 4-Dihydropyridines. *Synthesis* **2006**(03), 451-454.

[30] Ahmet Hacımüftüoğlu, Nurullah Saraçoğlu, Sana Saffour, Nadeem Abad, Yunus Kesgun, Nadjiba Zegheb, Ersin Gundeger, Fatma Yeşilyurt, Merve Nur Ataş, Gizem Bati-Ayaz, Öznur Altunlu, Burak Çınar, Mehmet Ali Yörük, Ufuk Okkay, Mustafa Özkaraca, Orhan Ateş, Ali Taghizadehghalehjoughi, Ferruh Lafzi, Hasan Türkez; Multi-Target Neuroprotective Compound Exhibits EAAT2-Modulating and Alzheimer's Pathology–Attenuating Effects in *in Vitro* and *in Vivo* Models. *ACS Chemical Neuroscience* **2026**, 17 (10), 1919–1944.
